# Ecological restoration at pilot-scale employing site-specific rationales for small-patch degraded mangroves in Indian Sundarbans

**DOI:** 10.1038/s41598-024-63281-8

**Published:** 2024-06-05

**Authors:** Krishna Ray, Sandip Kumar Basak, Chayan Kumar Giri, Hemendra Nath Kotal, Anup Mandal, Kiranmoy Chatterjee, Subhajit Saha, Biswajit Biswas, Sumana Mondal, Ipsita Das, Anwesha Ghosh, Punyasloke Bhadury, Rahul Joshi

**Affiliations:** 1 Environmental Biotechnology Group, Department of Botany, West Bengal State University, Berunanpukuria, Malikapur, Barasat, Kolkata, 700126 India; 2 Sarat Centenary College, Dhaniakhali, Hooghly, West Bengal 712302 India; 3 Department of Statistics, Bidhannagar College, Salt Lake City, Sector 1, Block EB, Kolkata, 700064 India; 4 Centre for Climate and Environmental Studies, Indian Institute of Science Education and Research Kolkata, Mohanpur, Nadia, West Bengal 741246 India; 5 Integrative Taxonomy and Microbial Ecology Research Group, Department of Biological Sciences, Indian Institute of Science Education and Research Kolkata, Mohanpur, Nadia, West Bengal 741246 India; 6 Zoological Survey of India (ZSI), Prani Vigyan Bhawan, Block M, New Alipore, Kolkata, 700053 India

**Keywords:** Ecological restoration, Degraded mangroves, Indian Sundarbans, Site-specific strategies, Mono- & multi-species assemblage, Indicators of restoration & self-sustenance, Ecology, Ecology, Environmental sciences

## Abstract

To date, degraded mangrove ecosystem restoration accomplished worldwide primarily aligns towards rehabilitation with monotypic plantations, while ecological restoration principles are rarely followed in these interventions. However, researchers admit that most of these initiatives' success rate is not appreciable often. An integrative framework of ecological restoration for degraded mangroves where site-specific observations could be scientifically rationalized, with co-located reference pristine mangroves as the target ecosystem to achieve is currently distinctively lacking. Through this experimental scale study, we studied the suitability of site-specific strategies to ecologically restore degraded mangrove patches vis-à-vis the conventional mono-species plantations in a highly vulnerable mangrove ecosystem in Indian Sundarbans. This comprehensive restoration framework was trialed in small discrete degraded mangrove patches spanning ~ 65 ha. Site-specific key restoration components applied are statistically validated through RDA analyses and Bayesian t-tests. 25 quantifiable metrics evaluate the restoration success of a ~ 3 ha degraded mangrove patch with Ridgeline distribution, Kolmogorov–Smirnov (K-S) tests, and Mahalanobis Distance (D^2^) measure to prove the site’s near-equivalence to pristine reference in multiple ecosystem attributes. This restoration intervention irrevocably establishes the greater potential of this framework in the recovery of ecosystem functions and self-sustenance compared to that of predominant monoculture practices for vulnerable mangroves.

## Introduction

This UN Decade on Ecosystem Restoration (2021–2030) pledges to repair and decelerate the degradation of ecosystems across the world^1^. This challenging promise signifies the necessity of developing effective restoration interventions for varying ecosystems. In the adaptive cycle^2^, the unpredictable ‘back loop’ unveils a phase of reorganization (α phase) allowing the disturbed ecosystem to redevelop on its own into a new r (re-assembling) phase. Human-assisted restoration intervenes at this phase to shorten this back loop and advances the fore loop directly back to the climax state (K phase) for the damaged habitat in a more or less predictable mode^3^. International principles and guidelines of ecological restoration^1,4,5^ place ecological restoration at the extreme right end along the restorative continuum offering a holistic approach relative to the applicable reference model, with rehabilitation, as one of the left allies. Of late, co-benefits of restoring/conserving mangrove ecosystems for climate change mitigation via high blue carbon storage^6–8^ have attracted worldwide interest in restorative activities for degraded mangrove ecosystems. However, for socio-ecological relevance of mangroves^8^, interventions that essentially fit in the left end of the restorative continuum, viz. conventional large-scale afforestation for silviculture comprising monogeneric planting of species like *Rhizophora* spp., *Sonneratia* spp., *Avicennia* spp., *Kandelia obovata* under Ecological Mangrove Rehabilitation (EMR) and Community Based Ecological Mangrove Rehabilitation (CBEMR)^9–24^, are predominant to re-establish degraded mangrove ecosystems, rather than applying the principles of ecological restoration. Mangrove ecosystem-design, the latest innovative mangrove rehabilitation alternative^7,16,17^was proposed for planting site-specific target species of local/regional needs with high CO_2_-sequestration potential with higher wood density, maximum canopy height, and litter nitrogen. Purely passive restoration may be the most cost-effective alternative^25^, allowing natural secondary succession to occur; however, anthropogenic forces, uncertainty in climate conditions, slow pace of recovery, non-availability of propagules, and too intense ecosystem damages changing the biophysical substratum beyond natural repair, often limit this natural process^4,26,27^.

Indian Sundarbans is contiguous with Bangladesh Sundarbans in the east, was declared a Ramsar site in 2019 (https://rsis.ramsar.org/ris/2370), and a UNESCO-proclaimed World Heritage Site since 1997^28^. Here, the mangrove habitats in settlement areas, are threatened with unrestricted loss and degradation due to primarily anthropogenic stressors (coastal development, aquaculture establishment, agriculture, timber, and fuel extortion) compounded with natural threats as well (recent cyclones Aila in 2009, Bulbul in 2019, Amphan in 2020, Yaas in 2021), and hold the remote possibility for secondary succession to set in voluntarily^29–32^. A unique geo-morphological feature caused the overall higher rate of erosion at the west-central part of the Indian Sundarbans between the Saptamukhi and the Gosaba estuaries^33^. The “Swatch of No Ground” submarine canyon located in this region acts as a primary barrier in sediment transportation and replenishment^33^, making mangrove restoration/plantations more challenging at these shorelines. The leading mangrove restoration interventions followed to date, worldwide and in Indian Sundarbans also, are often criticized to be less successful^11,16–18,22,24,34–36^. Provisions of ecosystem services viz. timber yielding, carbon storage for earning carbon credits, could be reinstated by afforested monoculture stands or species-poor rehabilitated mangroves^23^, nevertheless, it is difficult to accept that it substitutes biological functions of a natural mangrove niche or compensates for losses of ecological multi-functions of a native mangrove ecosystem at its integrity^17,18,22,34^.

Conventional mangrove rebuilding ventures often ignore essential site-specific secondary succession prerequisites (strategies to be based on co-located reference mangroves and related baseline data), for survival and sustainability of re-planted initiatives in an all-together standalone niche with multi-dimensional stress factors, like vulnerable mangrove ecosystem of Indian Sundarbans. A distinct knowledge gap still exists globally, on scientific designing and implementation of successful ecological restoration projects for degraded mangroves^22^. The key question addressed in this study is whether pre-analyzed site-specific ecological features associated with mangrove restoration and implemented site-specific strategies based on those attributes could lead to the successful experimental designing of a scientific restoration framework for highly stressed mangrove ecosystems similar to Indian Sundarbans. The following objectives were aimed to answer the key question: (1) on-site application of an experimental restoration framework at smaller scales that integrates site-specific scientific rationales, both conventional and non-conventional components & (2) use of a set of quantifiable indicators (conventional as well as innovative, some typical of local mangroves) to evaluate restoration success at the early phase of re-organization (within 2–6 years) with time, in comparison to co-located reference pristine mangroves and old monoculture mangrove sites.

This ecological restoration initiative for degraded mangroves has been undertaken at settlement fringe areas of the western part of Indian Sundarbans, since 2014, with collaborative support from the Department of Biotechnology, Govt. of India and the Forest Department, Govt. of West Bengal. This experimental biorestoration project was earlier cited^37–43^ and the technology applied under each objective was described explicitly in the supplementary information and methods section of the manuscript. Under the first objective, the key features of the nature-based restoration framework developed are based on the lessons learned by observing closely the natural mangrove establishment process in Indian Sundarbans, in the secondary or primary successional stage^44,45^.

We applied three key less-explored conventional components in unison to see its collective response (Supplementary Data 1, henceforth referred to as S1) at field-level restoration: (1) Grass-assisted stabilization: initial stabilization of the degraded patch with four local halophytic grasses (established by our group^46^); (2) Multi-species assemblage: multi-species composition (native true mangrove and mangrove associate species assemblage) as close to hinterland pristine (pristine refers to comparatively least disturbed natural mangroves under both protected and non-protected areas of Indian Sundarbans) reference mangroves as target species composition and human-assisted large scale-plantation guided by on-site salinity gradient, differential salinity tolerance levels of planted species and their inherent osmotic acclimation response; (3) Facilitative interaction: dense spacing plantation style capitalizing on density-dependent positive facilitation instead of even-distance-spaced afforestation pattern followed in terrestrial afforestation ventures. Other two non-conventional approaches tried with comparatively smaller datasets (S1) were (1) Growth promotion by onsite PGPR (plant growth promoting rhizobacteria) consortia addition, facilitating rhizosphere enrichment with native PGP root endophytic bacteria isolated from mangrove species by our group (Table S4, Figs. S10, S19–S21) and (2) Seed ball use^47^ to economize both nursery maintenance cost and low-saline soil usage for three threatened species *Heritiera fomes*, *Phoenix paludosa*, *Brownlowia tersa* (Fig. S7, Table S7). These threatened species are freshwater-loving mangroves and mangrove associates, those at the initial stage of establishment in nurseries require low-saline soil (EC ~  < 1 dSm^−1^), which is truly of limited availability in these river shores of Indian Sundarbans except during monsoons.

The key conceptions exploited in this restoration intervention are (1) maximally diverse community with species-specific independent ecosystem functions could only lead to ecosystem-scale restoration; (2) native halophytic grasses can act as primary foundation species, pioneer colonizer, ecosystem stabilizer and propagule trap in erosion-intensive sites^46^; (3) gradient of salt tolerance & differential positive/negative interactions among species control obvious species assemblages^48–52^; (4) osmotic acclimation is indicative of developed in-species resilience and cryptic habitat degradation^30^; (5) native epibiota^53,54^ and microbiota^55–57^ are equally imperative and crucial in mangrove ecosystem restoration; (6) large-scale human-assisted plantations with multiple native species should follow the site-preferred assemblages and successional trends to compensate for obliterated natural secondary succession.

A comprehensive site-specific baseline data was generated from ~ 40 hinterland degraded fringe mangrove patches in non-protected areas, co-located reference pristine mangrove stands, and some local conventional mono-species mangrove afforestation sites. This included site-specific information on foundation species, nurse species, their possible facilitatory roles, positive or negative effect on the spatial aggregation of species (conspecific or interspecific)^48–52,58,59^, timings of ‘windows of opportunity’ for seedling establishment^60–62^, site-specific hydrology, freshwater availability, species diversity, composition, density, distribution, site-specific abiotic factors^44,45^ like edaphic criteria of sediments viz. nutrients, texture, pH and salinity profiles, vulnerability to erosion, the threshold of sediment salinity hindering propagule germination/sapling growth, unfavorable sediment texture for initial colonization of seedlings, all in-tandem helped to identify the site-specific stressors to overcome. These robust onsite ecological/environmental baseline records developed are outcomes of extensive field-based surveys and lab-based analyses (2014–2022) (S1), unalike to the machine learning algorithm-based habitat suitability assessment of mangrove species for prioritizing restoration in protected Sundarban Biosphere Reserve (SBR)^31^.

Under the second objective, the quantifiable metrics utilized to evaluate the success of restoration are (1) species composition, richness, function and structure of mangroves^44,45^; (2) edaphic factors, physical and biochemical^44,45^; (3) genomic abundance and density profile of nutrient-cycler microbiota in sediment^55–57^; (4) osmotic acclimation of mangrove species^30^; (5) reproductive/pollination success, pollinator diversity, frequency of visits^63–65^; (6) epifaunal diversity and abundance^53,54^; (7) post-planting onsite natural colonization/recruitment of seedlings^66^.

**Figure 1:**
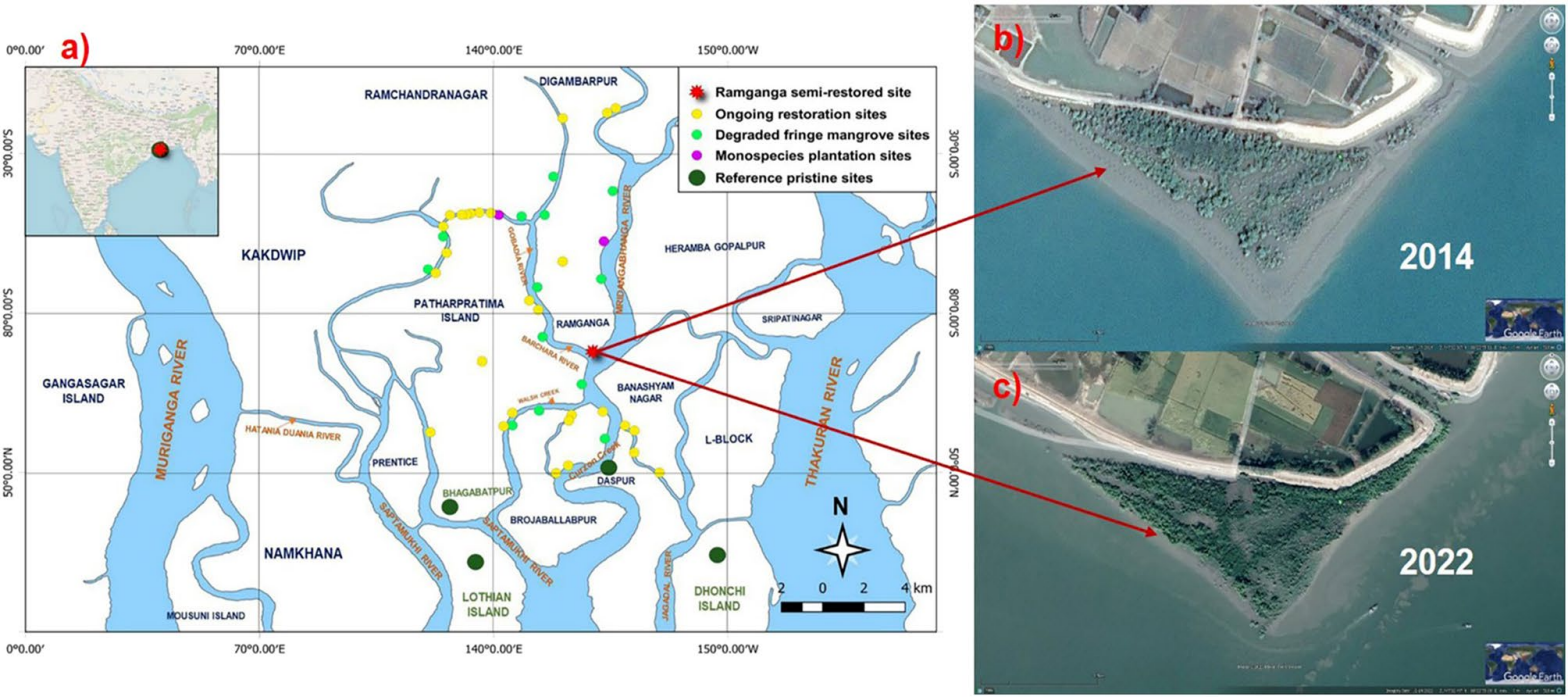
(**a**) Map representing the locations of Ramganga (the experimental semi-restored site), other ongoing restoration sites, degraded fringe mangrove sites, monospecies plantation sites, and reference pristine sites. Circles with red color denotes Ramganga semi-restored site, yellow color denotes other ongoing restoration sites, light green color denotes degraded fringe mangrove sites, violet color denotes monospecies plantation sites, and dark green color denotes reference pristine mangroves at their actual locations. (**b**) Google Earth image of Ramganga (the experimental semi-restored site) in the year 2014 when restoration was just initiated. (**c**) Google Earth image of Ramganga (the experimental semi-restored site) in the year 2022. Map-based illustrations for denoting geographical locations in (**a**) were executed through open access QGIS (version 3.28.3) (https://qgis.org/en/site/forusers/download.html) and Google Earth images, (**b**,**c**) were exported from open access Google Earth Pro (version 7.3.6.9750, 64-bit) (https://www.google.com/intl/en_in/earth/about/versions/#download-pro).

The success of key components of the biorestoration technology applied for ~ 65 ha degraded mangroves (31 discrete sites) (Fig. 1) was validated by statistical methods like RDA biplots^55,67^ and Bayesian t-tests^68^ where all variables characterize sub-components of the followed technology and its restorative outcomes. To highlight the success in a holistic approach, the evident closeness of a 6–7-year-old ~ 3 ha semi-restored site (developed during 2014–2022), to its pristine reference was demonstrated all through 2014–2022, via Ridgeline distribution^69^, Kolmogorov–Smirnov (K-S) tests^70,71^, and Mahalanobis Distance (D^2^)^72^ measure which irrevocably substantiates the applicability of this site-specific bio-restoration technology and its components.

## Results

### Redundancy analyses of the components of restoration framework vis-à-vis success outcomes

**Figure 2:**
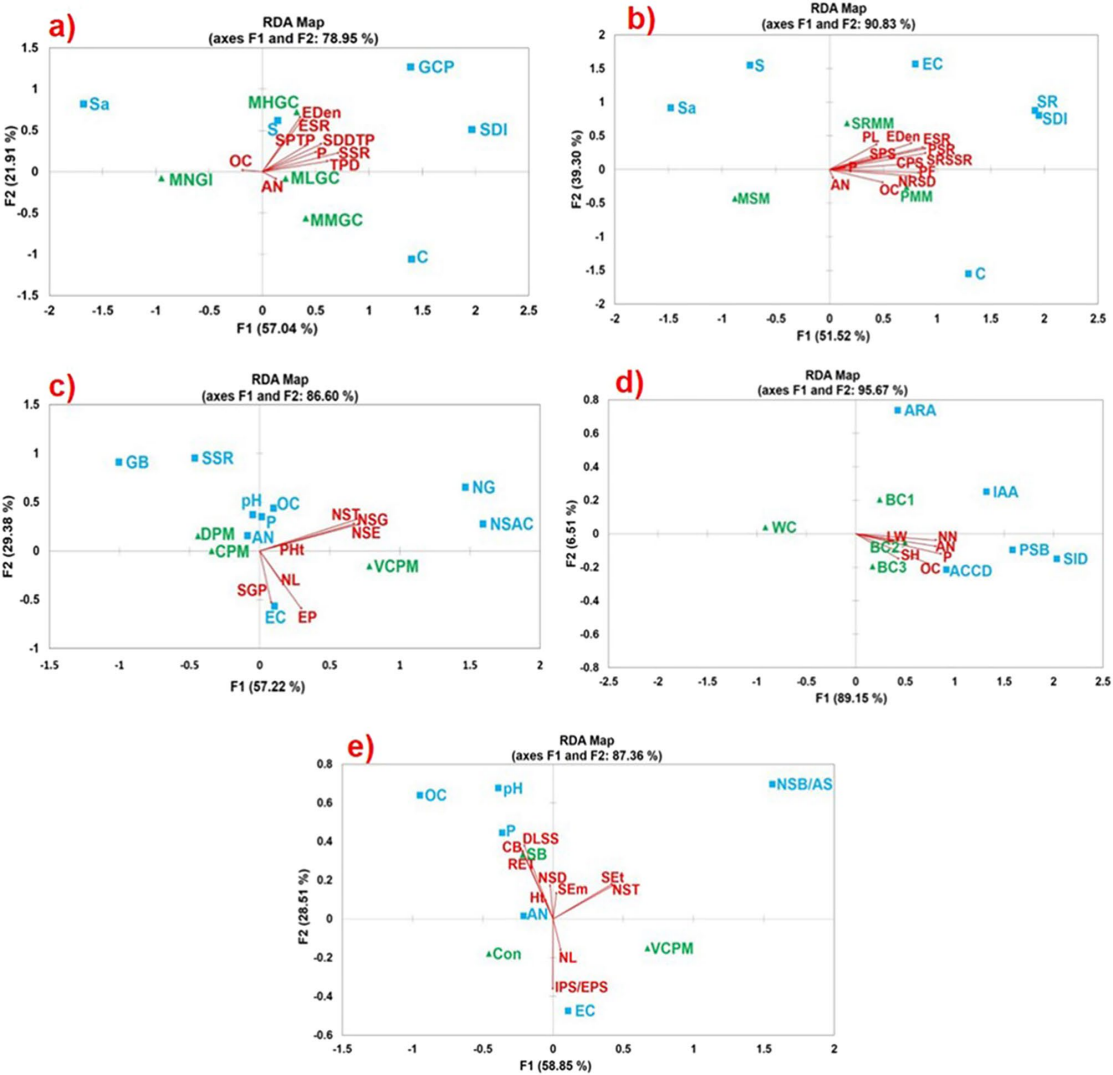
Bi-plots generated by canonical redundancy analysis (RDA) illustrating the application of different components of restoration framework and their success towards ecological restoration measured with different environmental and eco-physiological quantifiable variable outcomes. (**a**) RDA for grass-assisted stabilization, (**b**) RDA for multispecies assemblage, (**c**) RDA for facilitative interaction, (**d**) RDA for growth promotion by PGPR consortia addition, (**e**) RDA for seed ball use. *TPD* trapped propagules density, *SDDTP* seedling density developed from trapped propagule, *SPTP* survival% of trapped propagules, *SSR* seedlings species richness, *Eden* epifaunal density, *ESR* epifaunal species richness, *OC* organic carbon%, *AN* ammonia nitrogen, *P* phosphorus, *MNGI* monospecies with no grass at initial stage, *MLGC* multispecies with low grass coverage, *MHGC* multispecies with high grass coverage, *MMGC* multispecies with medium grass coverage, *SDI* Shannon diversity index, *GCP* grass cover%, *Sa* Sand%, *S* Silt%, *C* Clay%. *SPS* self-pollination success, *CPS* cross-pollination success, *PL* pollen load, *PF* pollinator frequency, *PSR* pollinator species richness, *Eden* epifaunal density, *ESR* epifaunal species richness, *OC* organic carbon%, *NRSD* naturally regenerated seedling density, *SRSSR* naturally regenerated seedling species richness, *MSM* monospecies mangroves, *SRMM* semi-restored multispecies mangroves, *PMM* pristine multispecies mangroves, *SR* species richness, *EC* electrical conductivity, *NST* number of seeds/seedlings transplanted, *NSE* number of seeds/seedlings established, *NSG* number of seeds germinated, *SGP* seeds germination%, *SPES* Survival% of established seedlings, *NL* number of leaves, *PHt* present height, *VCPM* very closely planted mangrove seedlings (≤ 5 cm), *CPM* Closely planted mangrove seedlings (≤ 40 > 5 cm), *DPM* distantly planted mangrove seedlings (≥ 100 cm), *NC* Number of clumps, *NSAC* no. of seeds/seedlings aggregated in a clump, *GB* glycine betaine, *SSR* soluble sugar-starch ratio, *PGPR *Plant growth promoting rhizobacteria, *LW* final leaf width, *SH* final shoot height, *NN* nitrate nitrogen, *WC* without consortium, *BC1* BC1 consortium, *BC2* BC2 consortium, *BC3* BC3 consortium, *SID* Siderophore%, *IAA* indole acetic acid produced, *PSB* P-solubilization, *ACCD* ACC deaminase units, *ARA* acetylene reduction assay. (**e**) *NSD* number of seed ball dispersed, *NST* number of seedlings transplanted, *SEm* number of seedlings emerged from seed balls, *SEt* number of seedlings established from transplanted seedlings, *CB* cost–benefit, *RET* reduction in establishment time, *Ht* height of seedlings, *NL* number of leaves, *DLSS* decrease in use of less saline soil, *IPS/EPS* emergence% of seedlings from seed balls/ establishment% of transplanted seedlings, *SB* seed ball technology, *Con* conventional technology, *VCPM* very closely planted mangrove seedlings (≤ 5 cm.), *NSB* number of seeds in a ball.

In redundancy analysis of grass-assisted stabilization (Fig. 2a) where grass cover% (GCP), Shannon diversity index (SDI) of restored species, onsite sand% (Sa), silt% (S), and clay% (C) were used as known variables, except ammonia-N (AN) and organic carbon% (OC), all other quantifiable outcome variables viz. phosphorus (P), epifaunal density (Eden), epifaunal species richness (ESR), trapped propagule density (TPD), seedling density developed from trapped propagules (SDDTP), survival% of trapped propagules (SPTP), species richness of regenerated seedlings (SSR), are found to have significant correlation (denoted by longer arrow lengths) being clustered at the same quadrant along with multispecies with high grass coverage (MHGC). Interestingly, the other three co-variables monospecies with no grass at the initial stage (MNGI), multispecies with low grass coverage (MLGC), and multispecies with medium grass coverage (MMGC), all were found to be located at different quadrants, with none of the outcome variables aligning with them excepting two with insignificant correlation viz. ammonia-N (AN) and organic carbon% (OC).

Similarly, in multi-species assemblage RDA biplot (Fig. 2b), when electrical conductivity (EC), species richness (SR), Shannon diversity index (SDI), sand% (Sa), silt% (S) and clay% (C) were used as known variables, except ammonia-N (AN), all quantifiable outcome responses like organic carbon% (OC), epifaunal density (Eden), epifaunal species richness (ESR), phosphorus (P), cross-pollination success (CPS), self-pollination success (SPS), pollinator frequency (PF), pollen load (PL), naturally regenerated seedling density (NRSD), naturally regenerated seedlings’ species richness (SRSSR), pollinator species richness (PSR), showed significant correlation (longer vectors) and was positioned in cluster being intermediately spaced between semi-restored multispecies mangroves (SRMM) and pristine multispecies mangroves (PMM), relating these two and representing closeness of SRMM with PMM despite their location at separate quadrants. Here again, monospecies mangrove (MSM) was found to be located in opposite separate quadrants, with none of the outcome vectors placed there.

Both the RDAs (Fig. 2a,b) explained 78.83% and 90.83% of total variations respectively. Also in these two RDAs, respective constrained variances, 83.43%, and 90.75% are much higher than the unconstrained variances (Table S9.1 & S9.2) that advocating maximum variance of the response variables is “redundant” with the variation of explanatory variables (S1).

On the other hand, in facilitative interaction RDA (Fig. 2c), environmental criteria to assess prevailing stress in the niche such as organic carbon% (OC), ammonia-N (AN), phosphorus (P), electrical conductivity (EC), osmotic resilience of planted mangroves based on accumulated glycine-betaine (GB) and soluble sugar-starch ratio (SSR) and typical attributes of planting patterns like the distance between individual seeds/seedlings planted viz. Very closely planted mangrove seedlings (VCPM, gap ≤ 5 cm), Closely planted mangrove seedlings (CPM, gap ≤ 40 > 5 cm) and Distantly planted mangrove seedlings (DPM, gap ≥ 100 cm), no. of seeds/seedlings aggregated in a clump (NSAC), no. of clumps/clusters (NC), were used as known criteria. The resultant outcomes viz. no. of seeds germinated (NSG), seed germination% (SGP), no. of seeds/seedlings transplanted (NST), no. of seeds/seedlings established (NSE), survival% of established seedlings (SPES), present height (PHt), no. of leaves (NL), showed significant correlations with known criteria used, depicted by longer vectors that are displayed across two quadrants. However, a closer spatial association of the outcome vectors with very closely planted mangrove seedlings (VCPM, gap ≤ 5 cm) was found, while other co-variables like closely planted mangrove seedlings (CPM, gap ≤ 40 > 5 cm) and distantly planted mangrove seedlings (DPM, gap ≥ 100 cm) were clustered in the same quadrant in opposite orientation to VCPM. Here although 86.6% of total variations are explained, unconstrained variance is much higher (62.71%) (Table S9.3), suggesting higher variance within individual co-variables (S1). In this RDA biplot (Fig. 2c), we have used dataset (S1) involving only the species that are usually difficult to establish onsite following conventional even-distance-spaced planting method, viz. *Heritiera fomes*, *Phoenix paludosa*, *Brownlowia tersa*, *Lumnitzera racemosa*, *Nypa fruticans* and *Xylocarpus* spp, as experienced by us (Fig. S6).

RDA biplots for onsite PGPR consortia addition (Fig. 2d) and seed ball use (Fig. 2e) explained 95.67% and 86.37% of total variations, however, here also unconstrained values are higher, 52.70% and 73.037% respectively (Table S9.4,5), because of existing higher variance within single co-variable than that across its datasets (S1). All the outcome variables in onsite PGPR consortia addition RDA (Fig. 2d) like shoot height (SH), rhizospheric ammonia-N (AN), nitrate–N (NN), phosphorus (P), organic carbon% (OC), except leaf width (LW), are significantly correlated (longer arrows) with the two PGPR consortia BC2 and BC3 in same quadrant over separately spaced BC1.

In seed ball use RDA (Fig. 2e), cost–benefit of transplantation (CB), reduction in establishment time (RET), and decrease in the use of less-saline soil (DLSS), are three major outcome variables having significant correlations (longest arrows) with known criteria of co-variables used and closely grouped with seed ball use (SB) in the same quadrant in contrast to the conventional technology (Con) and very closely planted mangrove seedlings (VCPM, ≤ 5 cm) located quite apart. These RDA biplots represented the success of the key components of the applied restoration method that were stated in the first objective and are validated with quantifiable metrics as stated in the second objective.

### Bayesian t-tests for variables used in RDA for further statistical validation

The unequal sample sizes for most of the variables, used in our RDA biplots (Fig. 2, Fig. S1) under the five key technology components applied (first objective), are not adequate to assume the normality of the variables under study. Therefore, we adopted the Bayesian t-test considering Jeffrey’s non-informative prior on both the means and gamma prior for the unknown population standard deviations to test for equality of population means from two independent samples with unequal variances, tested against the two-way alternative that the means are unequal. Here Bayes factor is used as an alternative to the conventional t-test that gives a natural and straightforward interpretation. This extensive hypothesis testing was performed for all the known and unknown variables used in RDA biplots for all possible paired combinations of the co-variables (qualitative) and resulting *p*-values (Fig. S22A,B) represent the null hypotheses of equality of population means between independent groups.

The pair monospecies with no grass at the initial stage (MNGI) vs. multispecies with high grass coverage (MHGC) (Fig. S22Aa,b; Table S10.1,2) showed significant *p*-values (< 0.05) for all known and unknown variables except silt% (known) and ammonia-N and organic carbon% in the result outcomes, that validates the location of the referred pair in diagonally opposite quadrants in RDA biplot (Fig. 2a). The placement of multispecies with low grass coverage (MLGC) & multispecies with medium grass coverage (MMGC) in the same quadrant is also explained by many a non-significant outcome (unknown) variables (*p* > 0.05) for MLGC-MMGC pair (Table S10.2).

In all possible pairwise combinations among monospecies mangroves (MSM), semi-restored multispecies mangroves (SRMM), pristine multispecies mangroves (PMM), most of the known variables are significant (*p* < 0.05), with one or two exceptions, that establish their distinct characteristic feature difference and placement at separate quadrants in respective RDA biplot (Fig. S22Ac, Fig. 2b, Table S10.3). In contrast, while for PMM vs. MSM and SRMM vs. MSM pairs, the same *p*-value trend continued for result variables (unknown) (Fig S22Ad, Table S10.4), PMM-SRMM pair showed several non-significant result variables (*p* > 0.05) (Fig. S22Ad), establishing near-approaching attributes of SRMM towards PMM and justifies the alignment of significant correlation vectors in between SRMM and PMM in the respective RDA biplot (Fig. 2b).

Similarly, the pair Very closely planted mangroves (VCPM) vs. Distantly planted mangroves (DPM) in facilitation interaction RDA (Fig. 2c), displayed most of the outcome variables having significant *p*-values (< 0.05) (Fig. S22Ae,f, Table S10.5,6), establishing the unambiguous difference in results for this pair, that justifies their locations at diagonally opposite quadrants in respective RDA biplot (Fig. 2c). In contrast, clustering of closely planted mangrove seedlings (CPM) vs. distantly planted mangrove seedlings (DPM) pair in the same quadrant is validated for all non-significant variables (*p* > 0.05) (Fig. 2c, Table S10.5,6). Most of the variables for many paired combinations in Fig. S22Ae,f being insignificant (*p* > 0.05), resulted in higher unconstrained variance in RDA (62.71%, Table S9.3).

In onsite PGPR consortia addition, the result (explanatory) variables for all possible combinations are significant in *p*-value (< 0.05) (Fig S22Bb, Table S10.8). In contrast, all known criteria except for BC2-BC3 pair seemed insignificant (> 0.05) (Fig S22Ba, Table S10.7). This might have caused BC2 and BC3 to cluster in the same quadrant in association with all significant correlation vectors in the respective RDA biplot (Fig. 2d).

In seed ball use component, most of the known variables are non-significant (*p* > 0.05) for all possible pairwise combinations (Fig S22Bc, Table S10.9), while outcome (unknown) variables for the pairs, seed ball technology (SB) vs. conventional technology (Con), seed ball technology (SB) vs. very closely planted mangroves (VCPM), are highly significant (*p* < 0.05) (Fig S22Bd, Table S10.10), that explains their locations in three separate quadrants in respective RDA biplot (Fig. 2e). Thus these Baysian t-tests results justified the RDA biplots appropriately.

### Ridgeline plots and Kolmogorov–Smirnov (K-S) tests of variable distribution across the timeline for the semi-restored mangrove patch of Ramganga

**Figure 3:**
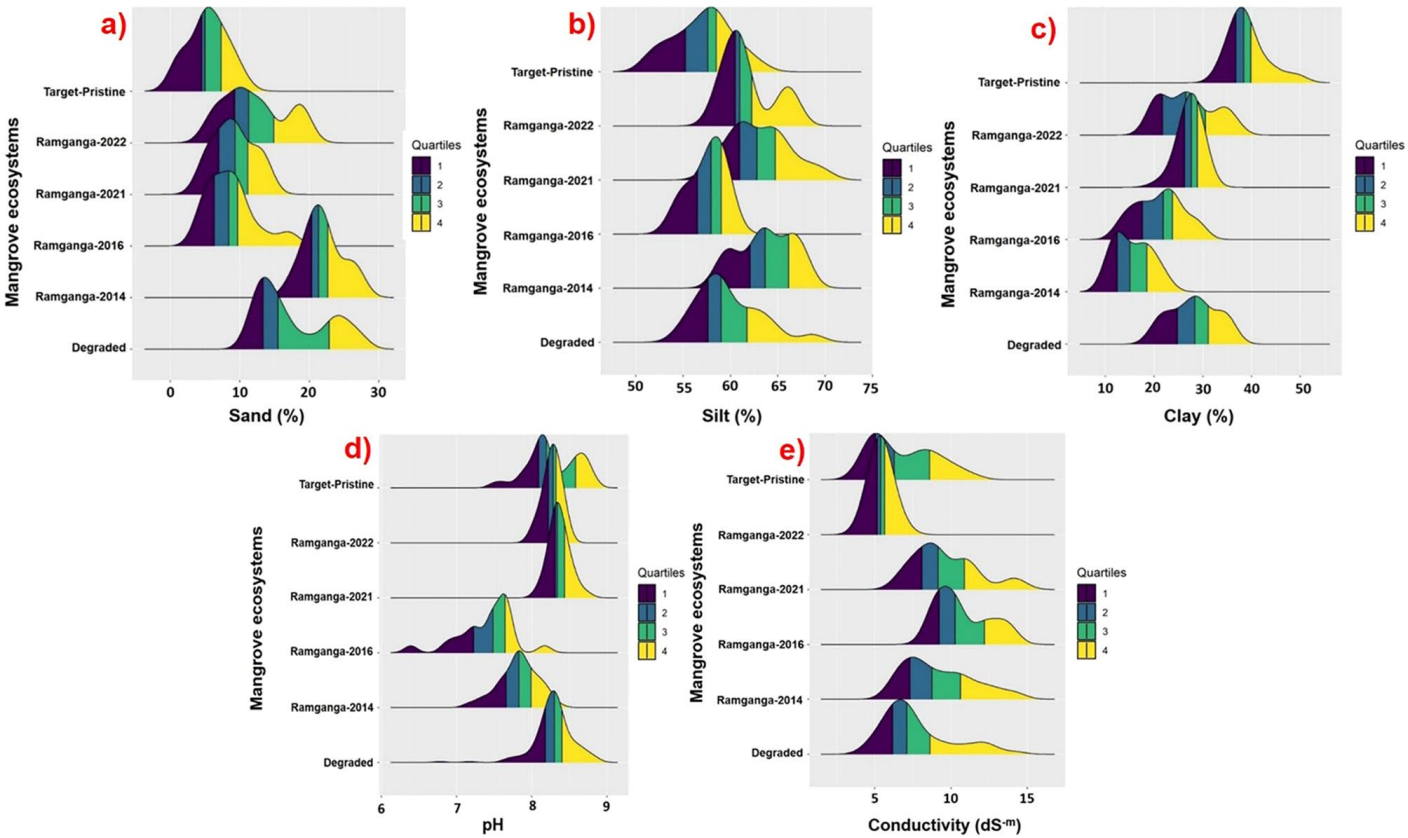
Ridgeline plots depicting the distributional changes of 5 different physical criteria of mangrove sediments across different states of mangroves viz. Degraded, Ramganga 2014, Ramganga 2016, Ramganga 2021, Ramganga 2022 and Target pristine reference mangrove forest (Y-axis) (**a**) Sand%, (**b**) Silt%, (**c**) Clay%, (**d**) pH, (**e**) Conductivity (dS^−m^). Here Ramganga site is the semi-restored site under restoration efforts since 2014.

Ridgeline plots (Fig. 3, Fig. S23A–D) are portrayed deciphering the comparative distribution of 25 quantifiable variables (S1) among six groups representing different states of mangroves, viz. Degraded, Ramganga 2014, Ramganga 2016, Ramganga 2021, Ramganga 2022, and Target pristine reference mangrove. Ramganga site referred to here at different time points, represents the site at Indian Sundarbans where we have applied the described restoration framework since its initiation in 2014 and evaluated all these 25 metrics at intervals till 2022. Most of these metrics of Ramganga 2022 depicted qualitatively their distribution very similar to the destination i.e. Target pristine reference mangroves, while each composite Ridgeline plot represented the respective distributions of values of quantified metrics gradually achieving closer to Target pristine reference mangroves, concurrently shifting away from the Degraded state (Fig. 3, Fig S23A–D).

However, Ridgeline plot depictions are again validated by running Kolmogorov–Smirnov (K-S) test statistic (Table 1) for comparing goodness-of-fit between distributions of each individual variable, out of 25 metrics (S1) across Target pristine reference versus each of the other 5 groups, representing different mangrove ecosystem status. Out of 25, 11 metrics displayed their distribution for Ramganga 2022, to be similar to that of the pristine state having non-significant *p*-values (*p* > 0.005–0.05), while only the same was observed for 5 and 2 metrics out of 25, respectively for Ramganga 2021 and Ramganga 2016. For Degraded and Ramganga 2014, the distribution of all 25 variables differs from that of the pristine state at a higher level of significance (*p* < 0.005–0.0005) with only one or two exceptions. The concept of lower significance with higher *p*-value (*p* > 0.005–0.05) and higher significance with lower *p*-value (*p* < 0.005–0.0005), applied in the K-S test for comparative evaluation of the distribution of 25 quantifiable variables across 5 different states of mangrove w.r.t. that of Target pristine mangrove, that endorsed conclusively their similarity (resemblance) and dissimilarity (difference) respectively with Target pristine mangrove (Table 1).

**Table 1:** Kolmogorov–Smirnov test analysis of 25 quantitative variables’ distribution with respect to that of the pristine state.

Sl. no	Name of variables	Degraded vs Pristine	Ramganga 2014 vs Pristine	Ramganga 2016 vs Pristine	Ramganga 2021 vs Pristine	Ramganga 2022 vs Pristine
1	Ammonia-N (mg kg^−1^)	1.709e−11(***)	1.058e−08(***)	0.0003839(***)	0.3491(^###^)	0.06297(^###^)
2	Organic carbon (%)	0.2447(^###^)	0.000138(***)	0.000996(**)	0.06113(^###^)	0.09463(^###^)
3	Aryl sulfatase activity (Units)	1.796e−11(***)	0.01228(^##^)	0.0006262(**)	0.0004807(***)	0.005857(^#^)
4	Sulfide-S (mg kg^−1^)	4.29e−08(***)	3.997e−15(***)	8.308e−08(***)	4.162e−10(***)	0.0002817(***)
5	Plant available-P (mg kg^−1^)	6.431e−12(***)	2.86e−06(***)	0.0001367(***)	0.4879(^###^)	0.8623(^###^)
6	Sand (%)	7.105e−14(***)	1.584e−06(***)	0.07081(^###^)	0.001424(**)	0.0005225(**)
7	Silt (%)	0.08152(^###^)	0.001332(*)	0.8674(^###^)	7.111e−06(***)	0.02806(^##^)
8	Clay (%)	0.000139(***)	4.417e−06(***)	1.715e−05(***)	8.603e−13(***)	3.687e−06(***)
9	Conductivity (dS^−m^)	4.427e−10(***)	6.147e−09(***)	2.139e−06(***)	1.658e−05(***)	0.02155(^##^)
10	pH	2.778e−11(***)	2.778e−11(***)	2.778e−11(***)	1.554e−14(***)	1.554e−14(***)
11	AB CFU g^−1^ dry sediment	2.778e−11(***)	2.778e−11(***)	2.778e−11(***)	2.778e−11(***)	2.778e−11(***)
12	CDB CFU g^−1^ dry sediment	2.778e−11(***)	1.554e−14(***)	1.972e−10(***)	1.554e−14(***)	1.972e−10(***)
13	FLNF CFU g^−1^ dry sediment	2.778e−11(***)	2.778e−11(***)	2.778e−11(***)	2.778e−11(***)	0.002318(*)
14	IOB CFU g^−1^ dry sediment	2.778e−11(***)	2.778e−11(***)	2.778e−11(***)	2.778e−11(***)	2.778e−11(***)
15	PSB CFU g^−1^ dry sediment	2.778e−11(***)	2.778e−11(***)	1.554e−14(***)	3.101e−10(***)	1.58e−06(***)
16	NB (AOB + NOB) CFU g^−1^ dry sediment	2.778e−11(***)	2.778e−11(***)	2.778e−11(***)	2.778e−11(***)	2.778e−11(***)
17	DB CFU g^−1^ dry sediment	1.972e−10(***)	1.972e−10(***)	0.002318(*)	1.292e−09(***)	0.281(^###^)
18	SOB CFU g^−1^ dry sediment	2.778e−11(***)	2.778e−11(***)	2.778e−11(***)	2.778e−11(***)	2.778e−11(***)
19	NOB CFU g^−1^ dry sediment	2.778e−11(***)	2.778e−11(***)	2.778e−11(***)	1.554e−14(***)	1.554e−14(***)
20	Fruit set in cross pollination (%)	2.778e−11(***)	2.778e−11(***)	2.778e−11(***)	0.0002468(***)	0.9062(^###^)
21	Fruit set in self pollination (%)	2.778e−11(***)	2.778e−11(***)	2.778e−11(***)	1.972e−10(***)	4.366e−08(***)
22	Frequency of pollinator visit (No. of visits m^−2^ h^−1^)	< 2.2e−16(***)	< 2.2e−16(***)	< 2.2e−16(***)	2.2e−16(***)	1.439e−05(***)
23	No. of species of pollinators	2.778e−11(***)	2.278e−11(***)	1.978e−11(***)	1.378e−11(***)	0.9062(^###^)
24	Accumulated soluble sugar: stored starch ratio	2.2e−16(***)	< 2.2e−16(***)	< 2.2e−16(***)	0.6971(^###^)	0.1081(^###^)
25	Accumulated glycine betaine (mg g^−1^ DW)	2.493e−12(***)	4.441e−16(***)	1.139e−05(***)	0.06805(^###^)	0.06124(^###^)

*AB* ammonifying bacteria, *CDB* cellulose degrading bacteria, *FLNF* free living nitrogen fixing bacteria, *IOB* iron oxidizing bacteria, *PSB* phosphorous solubilizing bacteria, *NB* nitrifying bacteria, *DB* denitrifying bacteria, *SOB* sulfur oxidizing bacteria, *NOB* nitrite oxidizing bacteria.

*p < 0.005, **p < 0.001, ***p < 0.0005, ^#^p > 0.005, ^##^p > 0.01, ^###^p > 0.05. Here Ramganga site is the semi-restored site under restoration efforts since 2014.

### Mahalanobis Distance (D^2^) measure to determine the holistic closeness of the semi-restored mangrove patch of Ramganga towards Target pristine reference

**Figure 4:**
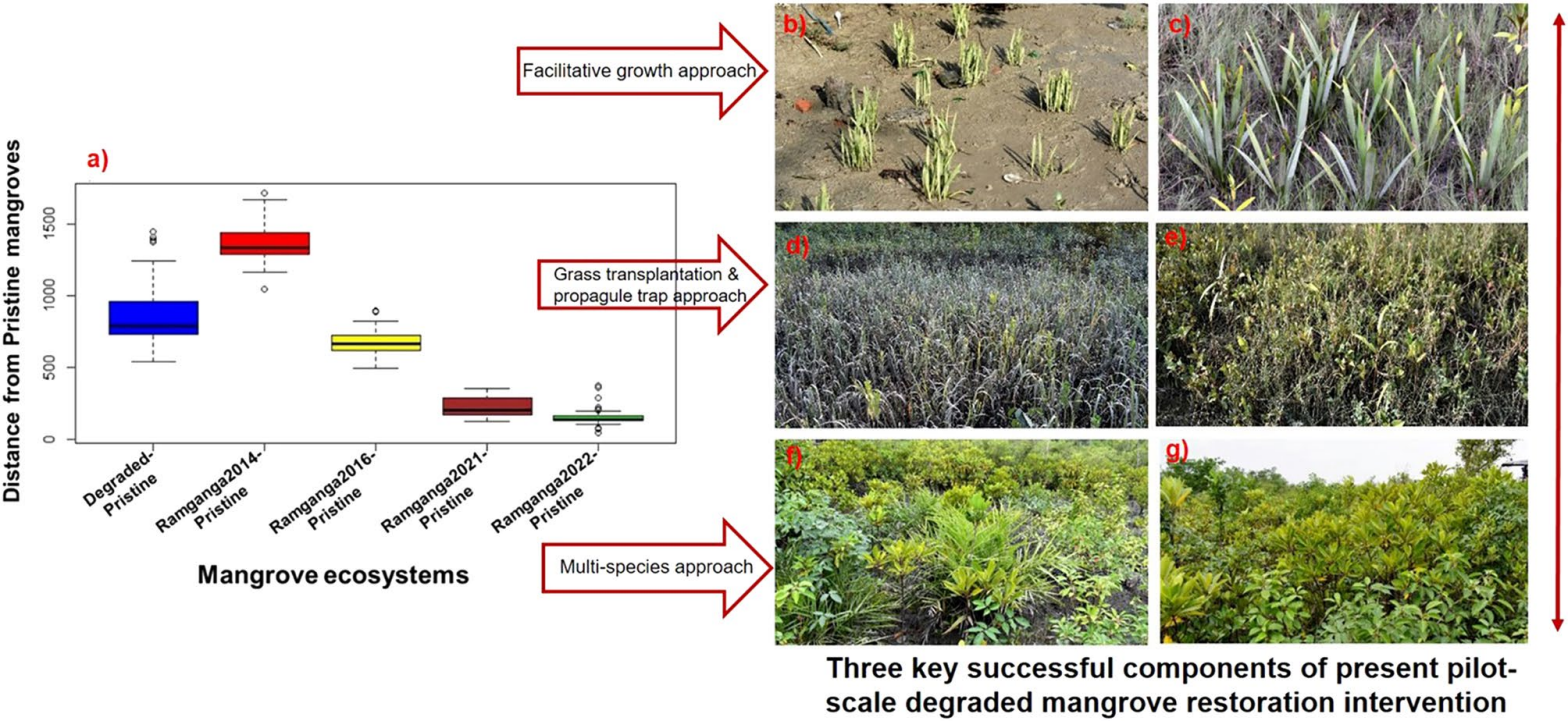
(**a**) Boxplot representing the sample distribution of Mahalanobis (D^2^) distance showing the semi-restored Ramganga site approaching ecologically towards Target pristine mangrove with the help of three key successful components of described restoration framework (**b**,**c**) Facilitative interaction demonstrated in *Phoenix paludosa* (Near threatened line)*,* (**d**,**e**) Grass assisted stabilization encouraging propagule trapping (**f**,**g**) Multispecies assemblage approach successfully applied at semi-restored Ramganga site.

In an aim to develop a measurable holistic approach to determine closeness towards Target pristine reference mangrove, we selected a sub-set of 13 quantifiable indicator variables out of the 25 variables (S1) viz. ammonia-N, sand%, clay%, conductivity, pH, organic carbon%, phosphorus (P), osmolyte glycine-betaine, osmolyte sugar starch ratio, pollinator frequency, fruit-set by cross-pollination, fruit-set by selfing and no. of pollinator species, based on their higher evaluation capability for restoration success, as considered by us. Out of these 13, 5 (clay%, sand%, pH, fruit-set by selfing, pollinator frequency) seemed to be still significantly different in distribution (p < 0.005–0.0005) from the Target pristine reference mangroves for Ramganga 2022, while the rest 8 showed to be similar to Pristine reference for Ramganga 2022 (p > 0.005–0.05) (Table 1). We considered these 13 variables in a combined manner to represent each of these 6 mangrove ecosystem categories, like Degraded, Ramganga 2014, Ramganga 2016, Ramganga 2021, Ramganga 2022, and Targeted pristine reference mangrove and subsequently computed Mahalanobis Distance (D^2^) measures for each of them to evaluate the distance from Target pristine reference mangroves (Fig. 4).

The plotted distance (D^2^) represented in boxplots (Fig. 4a), proved that Ramganga 2022, is having the shortest distance from the Pristine reference, among all the other mangrove ecosystem categories. It is interesting to note that Ramganga 2014 demonstrated an altogether severely degraded mangrove state, being farthest from Pristine reference mangrove in comparison to that of standard degraded mangrove states in Indian Sundarbans, based on our collected dataset (S1). As the distance from Pristine reference mangroves is gradually reducing with time for Ramganga site, after application of our site-specific nature-based restorative strategies (Fig. 4b–g), Ramganga 2022 (Fig. 1c) appears to be equivalent to pristine state statistically, although we acclaim this site as in “semi-restored” state of mangroves in Indian Sundarbans with regards to its yet-to-be-fully-recovered ecological functional status. This concluding statistical measure endorsed the success of the developed restoration framework and it undoubtedly answered the key question of this study.

### The differential genomic abundance of nutrient-cycling bacterial groups as an indicator for microbial functionality evaluation in ecologically restored mangroves of Ramganga

**Figure 5:**
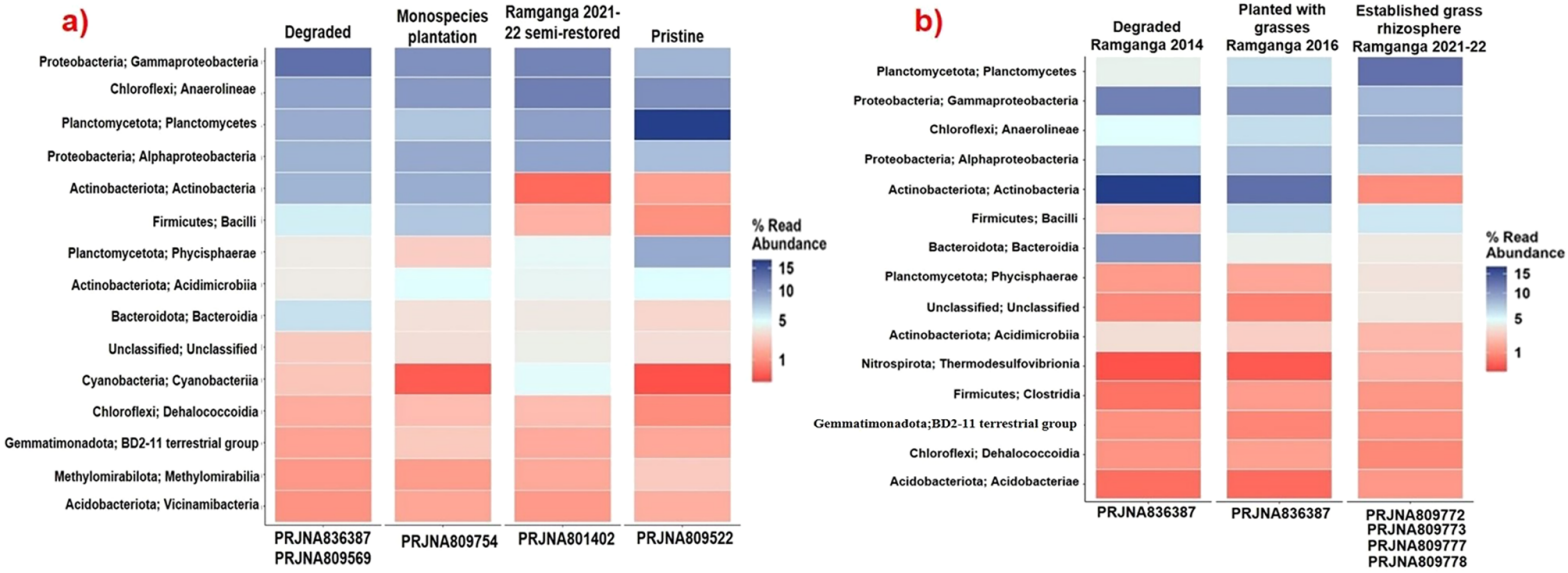
Heatmap displaying top 15 bacterial classes (Y-axis) defined from (**a**) sites at 4 distinct ecological status of mangroves and from (**b**) established mangrove grass rhizospheres from the semi-restored site (all in columns). The relative 16S gene abundance is shown in a 1–15% scale with three color variants. 16S gene read abundances with more than 5% are in the blue gradient and below 5% are in the red gradient. The heatmap was built on different biosamples’ reads from each ecological state which are included in the NCBI Bioprojects (X-axis).

It is already evident from earlier research that transitions in above-ground species composition during mangrove succession/restoration, are linked with concurrent differential soil decomposer/nutrient cycler community abundances^55–57^, expected to be following some distinctive array depending on the nature of species-specific root exudates attracting microbial community at the rhizospheric level of mangrove species^55^. Averaged abundance of reads of bacterial 16S gene (within 1%-15% scale) from composite sediment samples from degraded mangroves (PRJNA836387 & PRJNA809569, total 5 Biosamples), monospecies mangroves (PRJNA809754 with 4 Biosamples), semi-restored mangrove site at Ramganga (PRJNA801402 with 4 Biosamples) and pristine reference mangrove (PRJNA809522 with 2 Biosamples), are demonstrated to be accompanied by some interesting trends to be treated as indicators of return of microbial functionality as close to Pristine reference mangrove habitat (Fig. 5, Table S12). A distinct decrease in Actinobacteria class abundance is observed in semi-restored (~ 0.4%) and pristine sediments (~ 1.4%) whereas degraded and monospecies mangroves displayed ~ 8% and ~ 9% abundance respectively (Fig. 5a). A similar declining trend is observed for the Bacilli class under phylum Firmicutes, ~ 5.6% at degraded and ~ 7.5% at monospecies, falling down to ~ 1.8% and ~ 1% in semi-restored and pristine habitat of mangroves respectively (Fig. 5a). Conversely, much higher abundance (~ 17%) was observed to be associated with pristine mangroves for Planctomycetes, a class under phylum Planctomycetota, against that of ~ 9%, ~ 7.5% and ~ 9.6% for degraded, monospecies and semi-restored sediments respectively (Fig. 5a). Phycisphaerae, another class under phylum Planctomycetota, showed a similar trend with ~ 9% abundance for pristine as well as ~ 4%, ~ 2.7%, and ~ 4.5% for degraded, monospecies, and semi-restored sediments respectively (Fig. 5a).

In addition, if average abundance of reads of bacterial 16S gene (within 1–15% scale) was considered across composite sediment samples from established halophytic grass rhizospheres only from our restoration sites (Fig. 5b), initial stage of Ramganga site at 2014, before it was stabilized with grasses (PRJNA836387 with 1 Biosample), Ramganga site midway at 2016 when grass community started to establish (PRJNA836387 with 1 Biosample) and during 2021–2022 from semi-restored Ramganga site with established four halophytic native grass rhizospheres, *Myriostachya wightiana*, *Paspalum vaginatum*, *Porteresia coarctata* and *Sporobolus virginicus* (PRJNA809777, PRJNA809772, PRJNA809778 and PRJNA809773 respectively, each with 1 Biosample), a steep rise in abundance for Planctomycetes (from ~ 5.4% to ~ 16.7%) and Phycisphaerae (~ 1.5% to 4.6%), two classes under phylum Planctomycetota was evident, while a sharp fall in abundance of Actinobacteria (from ~ 22.2% to ~ 1.12%) can be observed (Fig. 5b), that strengthened further the trend observed in Fig. 5a. An increase in abundance (from ~ 6.3% to ~ 11.8%) of Anaerolineae community, a class under Chloroflexi phylum is also found to be associated especially at the grass rhizosphere (Fig. 5b), that finally reaches an overall abundance of ~ 11.85% at Ramganga site during 2021–22 with ~ 10.8% as composite abundance at nearby pristine mangroves. These preliminary analyses of NGS metadata (Table S12.1,S12.2) under these NCBI Bioprojects are presented as suggestive microbial signatures in an attempt to evaluate restoration success demonstrating the genomic abundance of microbiota in semi-restored site sediments approaching a similar profile to that of pristine reference sites to some extent in contrast to monospecies mangrove sites. Nonetheless, more Biosamples across seasons at different time intervals are needed to substantiate these observed trends conclusively.

### Natural seedling recruitment as a self-sustenance indicator for ecologically restored Ramganga site vis-à-vis monoculture mangroves of Indian Sundarbans

**Figure 6:**
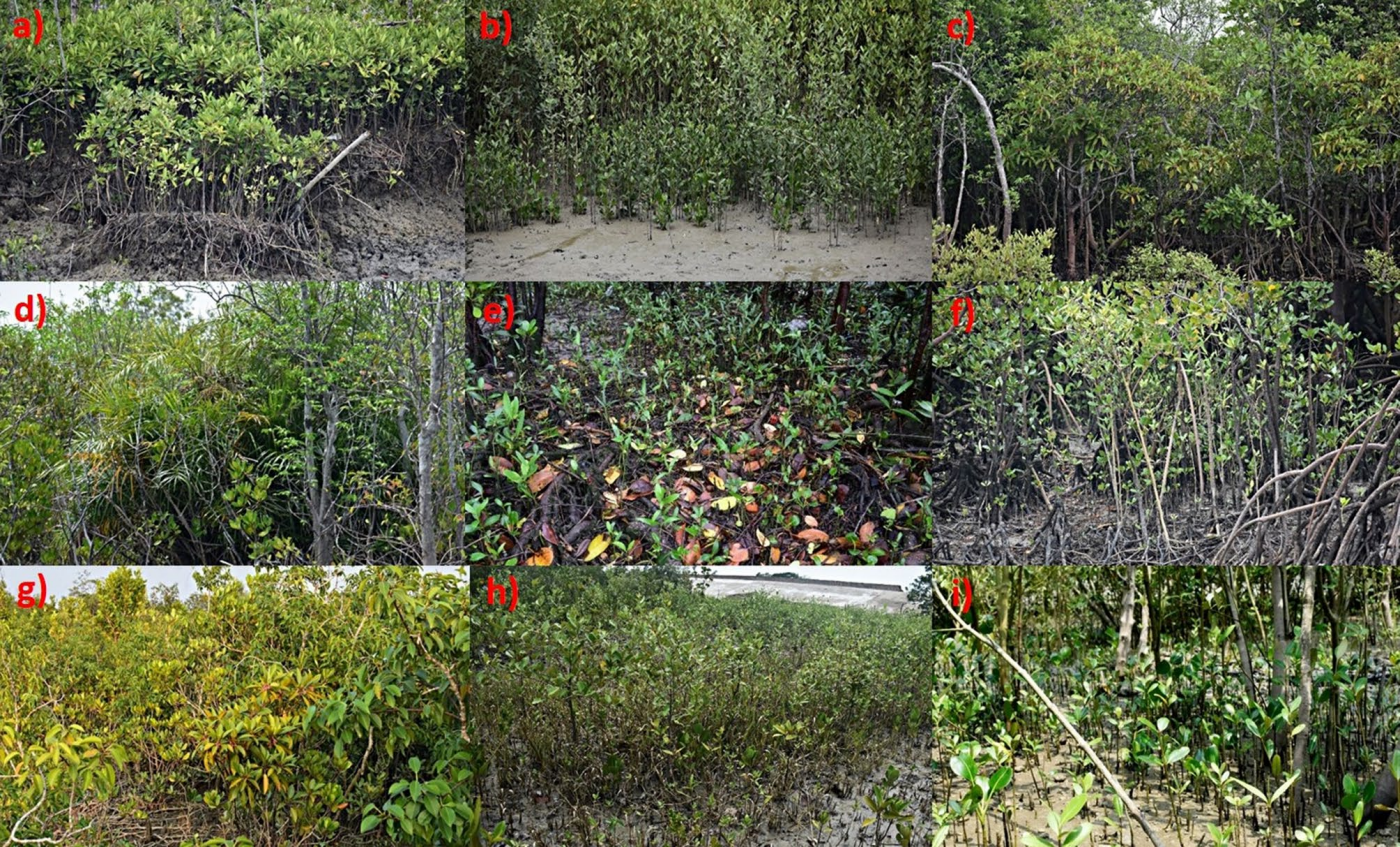
Glimpses of natural pristine mangroves of Indian Sundarbans vis-à-vis our restored site at Ramganga, (**a**,**b**) Natural *Avicennia alba* dominated mono-species mangroves with naturally recruited new seedlings in each season at the sites, (**c**,**d**) Natural multispecies pristine mangroves with *Bruguiera gymnorrhiza* also rarely interspersed in the community in **c** (**e**,**f**) Natural multispecies pristine mangroves with rich new recruits of seedlings, (**g**–**i**) Our multispecies semi-restored site at Ramganga (**g**), inviting naturally regenerated seedlings under its canopy each season showing its return to self-sustainability comparable to that of pristine reference mangroves (**h**,**i**).

When a mangrove site starts to facilitate post-planting colonization by naturally regenerated non-planted seedlings, no more human assistance is needed for restoration activities to continue further^66^. This “catalytic effect”^66^on natural recruitment of seedlings is very characteristic of pristine mangrove habitats both for natural monospecies (Fig. 6a & 6b) or multi-species mangroves (Fig. 6e,f) in Indian Sundarbans, where seed dispersal, propagule trapping, and their establishment into newly recruited seedlings occur in regular mode in each season, eventually enriching the mangrove density and forest expansion with time (Fig. 6c,d). Our group attempted to quantify and use this typical attribute of naturally recruited seedlings establishment as a metric to evaluate restoration success in this study (Figs. 2b, 8a,b). Species richness and density of on-site colonized, post-planting naturally recruited seedlings were quantified across the Ramganga restoration site in time series of 2014, 2016, 2021, and 2022 in comparison to co-localized pristine, degraded, and monospecies reference mangrove habitats at Indian Sundarbans (Fig. 8a,b). Species richness of naturally regenerated mangrove seedlings steadily increases at the Ramganga site from 2014 to 2022 (from 4–5 to 12–14) vis-à-vis the same as 15–18 at Pristine and only 2–3 at monospecies afforestations (Fig. 8a, Table S8). Similarly, a steep increase in natural regeneration density at Ramganga site (~ 40 fold) was observed from 2014 to 2022 (Fig. 8b). However, this semi-restored site is yet far behind in achieving natural seedling recruit density at par with pristine mangrove habitat (Fig. 8b). Pristine mangrove understory (Fig. 6e,f) and Ramganga semi-restored site floor (Fig. 6h & i) are found to be discernibly densely crowded with naturally regenerated seedlings.

**Figure 7:**
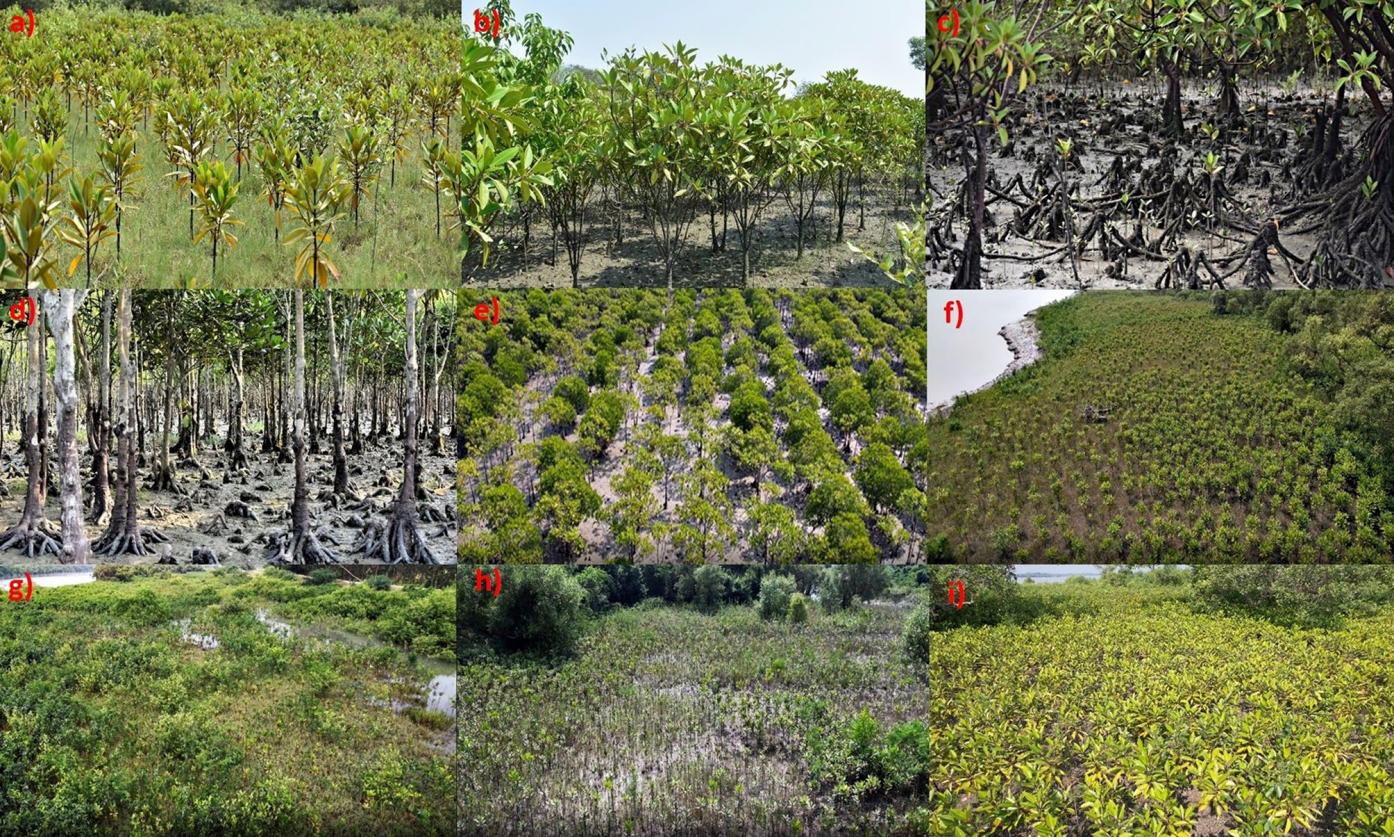
Glimpses of the conventional restoration ventures with monospecies *Bruguiera gymnorrhiza* at Indian Sundarbans (**a**–**e**), monospecies *Bruguiera gymnorrhiza-*planted sites under different stages of developments from Indian Sundarbans with almost barren understories (**d**,**e**), sometimes with sparse grasses *Sporobolus* sp., *Paspalum* sp., or *Suaeda* spp. (**a**,**b**), scanty seedlings of its own (**c**), (**f**–**i**) Contrasting sights from our different multispecies restoration sites, planted with *Bruguiera gymnorrhiza* amid additional species assemblages.

**Figure 8:**
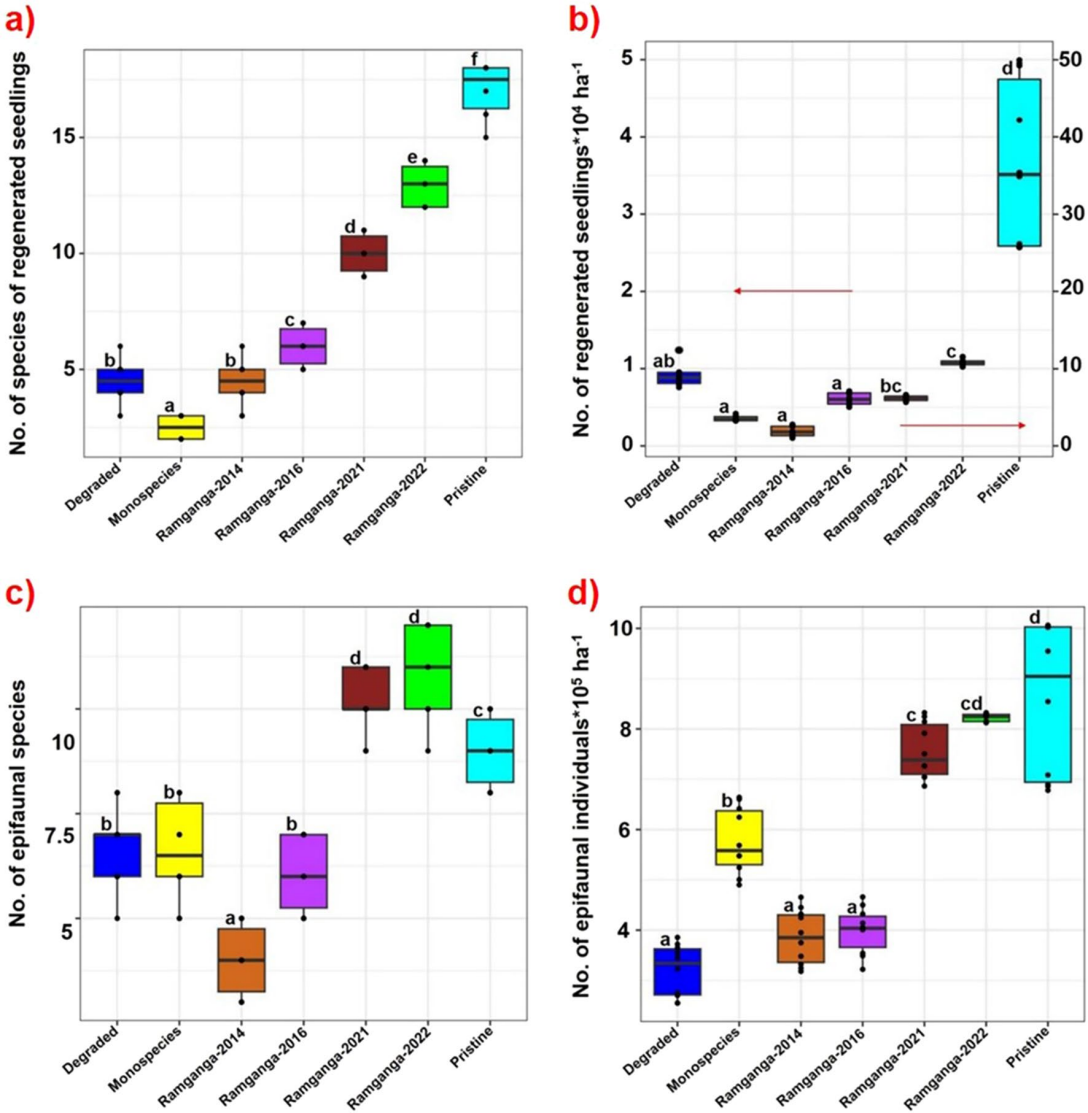
Box-whisker-dot plot displaying the data of regenerated/naturally growing seedlings and the abundance of epi-fauna across different states of mangroves viz. Degraded, Ramganga 2014, Ramganga 2016, Ramganga 2021, Ramganga 2022 and Target pristine reference mangrove forest. Here Ramganga site is the semi-restored site under restoration efforts since 2014. (**a**) No. of species of regenerated seedlings, (**b**) No. of regenerated seedlings ha^−1^, (**c**) No. of epifaunal species, (**d**) No. of epifaunal individuals ha^−1^. Whiskers represent the range of data from highest to lowest with a median value and the dots are the number of data (n) used for analysis. Here n = 10. Values designated with different letters are significantly different at the 5% level.

In contrast, an almost visibly barren understory of local monogeneric plantations with *Bruguiera gymnorrhiza* (Fig. 7b–e) was validated with ~ 25–30 fold lower density of naturally recruited seedlings of the semi-restored Ramganga site in 2022 (Fig. 8b). In Indian Sundarbans, *Bruguiera gymnorrhiza* is never observed to be a dominant constituent species in natural populations (being only 0.41–1.17% of species composition of co-located pristine reference mangroves, highlighted in Table S1) and it is never found to colonize in a naturally aggregated mono-species stands like *Avicennia* spp., *Sonneratia* spp., *Phoenix paludosa* or many other mangrove species, rather is witnessed to grow as isolated members in a natural mixed species population (Fig. 6c).

Interestingly, since long, conventional mangrove restoration practices in Indian Sundarbans have been continued with propagules of this particular species which have now led to establishment of several monospecies *Bruguiera gymnorrhiza* patches across the settlement area shoreline fringes (based on our observations during 2014–2022) and subsequently resulted into highest abundance and availability for this particular propagule throughout the year in Indian Sundarbans, for conducting conventional mangrove afforestation ventures. Neither a pioneer species, nor a natural colonizer, *Bruguiera gymnorrhiza*, intriguingly, has a high survival rate, good establishment efficiency, excellent vegetative growth, and highly successful reproduction leading to high fruit-set percentage at monospecies restoration sites (Fig. 7a–e, Table S1).

Surprisingly, the understory of these species either developed scanty seedlings of its own (Fig. 7c) or remained barren, even after 18–20 years of initial plantation (Fig. 7c–e), or sometimes got colonized sparsely with grasses like *Sporobolus* sp., *Paspalum* sp. or *Suaeda* spp. (Fig. 7a,b) at 3–5 year-old stands. In our sites of restoration (Figs. 6g, 7f–i), we also introduced and established *Bruguiera gymnorrhiza* species, however, interspersed with other mangrove and associate species and contrastingly observing profuse post-plantation natural recruits (Fig. 6h,i), signified as an efficient measurable indicator. This feature could distinguish visibly at preliminary stages, between mono-species mangrove afforestations and multi-species ecologically restored mangroves in approaching self-sustainability/functional independence of the respective ecosystem, comparable to that of pristine reference mangroves.

### Epifaunal diversity, abundance, and sediment physical criteria as conventional indicators for ecologically restored Ramganga mangroves and other ongoing restoration sites

In addition, epifaunal diversity and abundance also was found to be on the rise across Ramganga 2014, Ramganga 2016, Ramganga 2021, Ramganga 2022, with Ramganga 2021, 2022 being comparable to that of pristine reference mangroves (Fig. 8c,d, Figs S15, S16, S17, Table S6). Nevertheless, monospecies and degraded sites failed to show even near-equivalence in epifaunal species richness and density when compared to that of Ramganga 2021, 2022, and pristine reference mangroves (Fig. 8c,d). It is suggested that the increasing density of epifaunal community is positively correlated to bioturbation activities caused by them that aerate mangrove sediments and hinder the reduction process of sulfate to sulfide by sulfate reducers^53,54^, a typical biogenic process occurring in mangrove sediments. Sulfide toxicity prevents seedling colonization^73^. Hence the epifaunal abundance could be a good metric for restoration evaluation in mangrove habitat which could indirectly prepare the habitat to be colonized by new seedling recruits^53,74^. It is noteworthy that among the epifaunal members, the site Ramganga 2021, 2022 is now experiencing the frequent visitation of locally rare-sited mangrove horseshoe crab (*Carcinoscorpius rotundicauda*) (Fig. S16c), referred to as “the living fossil”^75,76^, known to colonize in pristine mangroves.

From our extensive field-based analyses we found that the co-located reference pristine mangroves in the western part of Indian Sundarbans are especially species-rich (~ 30–34 species observed, including 13 as rare and threatened ones, Tables S1,S2). In contrast, hinterland fringe mangroves and degraded mangrove habitats at settlement areas are extremely species-poor inhabited by maximally 6–12 very common (most abundant) species with 0–2 rare and threatened (RET) species with declining population trends (Tables S1,S2). Among the abundant ones, *Avicennia marina* and *Avicennia alba* are very successful in establishing in most of the fringe mangrove habitats of Indian Sundarbans (Tables S1,S2) due to their wide range of acclimability for sediment texture, pH, and salinity criteria, required for initial colonization, contrary to other RET mangroves having a narrow window of acclimation in this regard (Fig. S24, Supplementary Data S1, Tables S1,S2). Our data presented here (Fig. S24, Supplementary Data S1) demonstrate that sediment texture, pH, and salinity of some of our ongoing restoration sites, Semi-restored Ramganga 2021 and Semi-restored Ramganga 2022, after initial grass colonization, are gradually approaching the edaphic physical criteria of natural mangrove populations at the rhizospheres (Fig. 3, Fig. S24). This has resulted in higher survival percentage (up to even above 90%) for several native mangrove species transplanted at these ongoing sites of restoration spanning ~ 65 ha in total (Tables S1, S3).

## Discussion

### Current mangrove restoration scenario across the world

Restoration of lost mangroves through human intervention is the call of the hour worldwide to mitigate the negative environmental impacts leading to climate change^27^. However, while natural regeneration (without human intervention) on favorable substratum also does occur^27,77^, the return of mangrove shields in damaged ecosystems is achieved conventionally via different modes of rehabilitation. Afforestation for silviculture^10^, large-scale monogeneric plantations^11,20,22,24,78^, combining mangrove shield with hard engineering (hybrid nature-based solution/hybrid engineering)^27,79–81^, Ecological Mangrove Rehabilitation (EMR) based on hydrological rehabilitation/major excavation/fill followed by natural regeneration or plantation^11–14,24^, Community Based Ecological Mangrove Rehabilitation (CBEMR), a modified version of EMR with local community involvement in planning, design, implementation and monitoring^24^, rehabilitation of mangroves in highly urbanized coastline by recreating the necessary biophysical conditions to support mangroves and its other ecosystem components^9,15^, nature-based infrastructure development with mangrove ecosystem management^9^ are the prevalent strategies. The latest proposition, yet to be implemented, is designing a novel-functioning mangrove ecosystem, based on locally or regionally preferred ecosystem services, and planting a pool of regionally available native mangrove species^7,16,17^.

### Earlier failures of conventional mangrove restoration approaches

Regardless of innumerable mangrove restoration projects implemented to date globally, most failed to meet their goals and rarely reported their success rate^11,16,18,22,24,27,34^, despite many optimized mangrove restoration guidelines/protocols being published and practiced all over the world^27,44,45^. The practice of afforestation by planting mangrove propagules from the single genus (*Rhizophora*) at lower intertidal elevations, has been criticized by the IUCN Mangrove Specialist Group (IUCN-MSG 2015) in the Philippines because of the very low survival rate of planted mangroves (10–20% or even lower)^24^. Sri Lanka, invested substantially (about 13 million USD), in the planting of mangroves (*Rhizophora* spp., being 97% of the plantations) over the past decade covering approximately 1000–1200 ha of area. However, the total surviving planted area was reported to be only about 200–220 ha^22^.

These monospecific plantations lack the habitat complexity and species diversity that reinforce comprehensive ecosystem function in degraded mangroves^11^. The causes for failures in these restoration ventures are attributed to a lack of scientific knowledge, wrong restoration site selection^22^, inappropriate species selection, ignorance about intertidal habitat conditions, hydrology, species-specific requirements^16,82^, baseline shifting^83^, lack of post-planting care and monitoring^27^, lack of community involvement, non-inclusion of social scientists^9,27^. IUCN mangrove expert group expressed concerns over “media-effective mass planting” with one/two species of easily available propagules or seedlings that create monoculture stands or species-poor restored plots which neither substitute the biological functions of a natural mangrove nor compensate for losses of mangrove ecosystems in the longer run^18^.

A recent quantitative analysis of mangrove restoration/rehabilitation outcomes^23^ (covering 22 countries, primarily from China, Vietnam, and the Philippines) reported that in 96.2% of cases, so-called restoration occurred via conventional single species plantation with *Rhizophora apiculata*/*Rhizophora mucronata*, or *Avicennia marina* or *Kandelia obovata,* sometimes also employing hydrological rehabilitation. However, the monospecific mangrove restoration method proved to be of limited success in achieving niche complementarity and in supporting benthic and terrestrial communities, when compared to a mangrove restoration intervention with diverse species conducted in Hainan, China^84^.

### Some remarkable successes of mangrove restoration across the world

Conversely, some success was achieved also. Since 1996, the Government of Bangladesh has successfully developed nearly 280 km^2^ of afforested mangroves on the Delta of the Ganges, Brahmaputra, and Meghna Rivers, in the east of the Bangladesh Sundarbans, primarily planting *Sonneratia apetala* and *Avicennia officinalis*^20^. These afforested mangroves are still flourishing even after 40 years and proved to be equivalent to natural mangroves in aboveground biomass/ecosystem services, nevertheless lagging in species richness and biodiversity^20^. In India, in the state of Gujarat, similar initiatives are being effectively implemented by the Gujarat Forest Department (GFD) under which approximately 50,000 ha of mangroves in coastal areas and islands of the state were planted with *Avicennia marina*, during 1983–1984 to 2007–2008, leading to very high success rate of survival and establishment^21^.

In El Salvador, 80 ha of dying mangroves could be restored by removing the hydrological barrier and applying the CBEMR methodology successfully^27^. Success via hybrid engineering was reported from a large-scale mangrove reforestation venture of nearly 9000 ha, located in front of 100 km of concrete sea-dyke in Vietnam that proved to be highly cost-effective, with Benefit–Cost Ratios (BCRs) varying from 3:1 to 28:1^85^. The concept of Ecosystem Design was validated using data from the Sundarbans Reserved Forest inventory in Bangladesh, from 150 plots across the Sundarbans Reserved Forest of Bangladesh^7^. These studies^7^ demonstrated successfully through structural equation models that blue carbon storage in mangrove ecosystems has a positive relationship with species richness^84^, functional diversity, functional composition of mangrove community, and diverse leaf litter traits, with a negative impact across sediment salinity gradient.

### The developed restoration framework vis-à-vis biodiversity conservation

**Figure 9:**
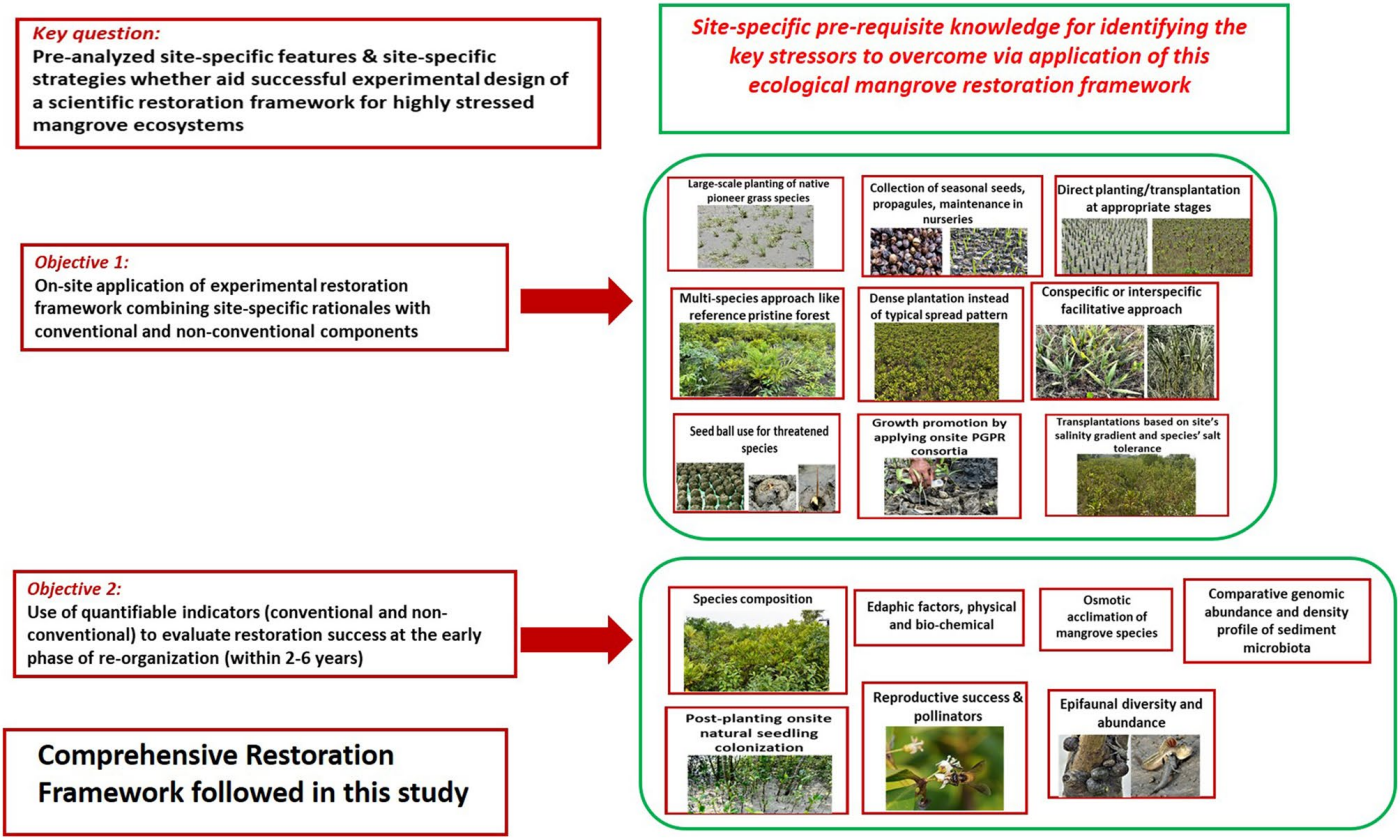
A Comprehensive Restoration Framework followed for the ecological restoration of degraded mangroves is presented in this study with the key question, site-specific prerequisites, and the achieved objectives with their components.

In this context, the pilot-scale comprehensive ecological restoration framework for degraded mangroves presented here (Fig. 9) is especially grounded on site-specific rationales identifying the key stressors responsible for hindering onsite secondary mangrove succession and aimed scientifically to overcome the same for risked shorelines of Indian Sundarbans. Conversion of small degraded mangrove patches to species-rich ecologically restored habitats might seem too trivial, of regional interest only, in the perspective of worldwide large-scale mangrove rehabilitation initiatives. However, if the goals of the United Nations Decade on Ecosystem Restoration 2021–2030 and the Convention on Biological Diversity Post-2020 Biodiversity Targets are to be achieved, these minor restoration projects might contribute towards “disproportionately high value for biodiversity”^86^.

Worldwide mangrove restoration innovations mostly aim for restoring larger coastal areas, following the most economically practicable trend of community-based mangrove rehabilitation which could eventually turn out to be the greatest GHG emission sinks and blue carbon reservoirs of utmost importance^6^ aiding climate change mitigation^87^. Our developed restoration framework is primarily targeted towards re-establishing the diverse species assemblages similar to that of the pristine reference mangroves in these small degraded patches nurturing them gradually into a self-sustainable functional mangrove ecosystem, extending the concept of mangrove restoration beyond just silviculture^88^. Whether this restoration approach would be successful in achieving the co-benefits of biodiversity conservation and carbon storage as well^84^, is to be evaluated in the years to come. Preference of creating high-biodiversity, low-carbon ecosystems over low-biodiverse (monotypic), high-carbon storage-capable plant communities, even for mangrove ecosystems, has begun to gain support from several quarters, a subject of long-standing controversy^7,89,90^.

### Consideration of semi-restored Ramganga mangrove site as potential OECM

**Figure 10:**
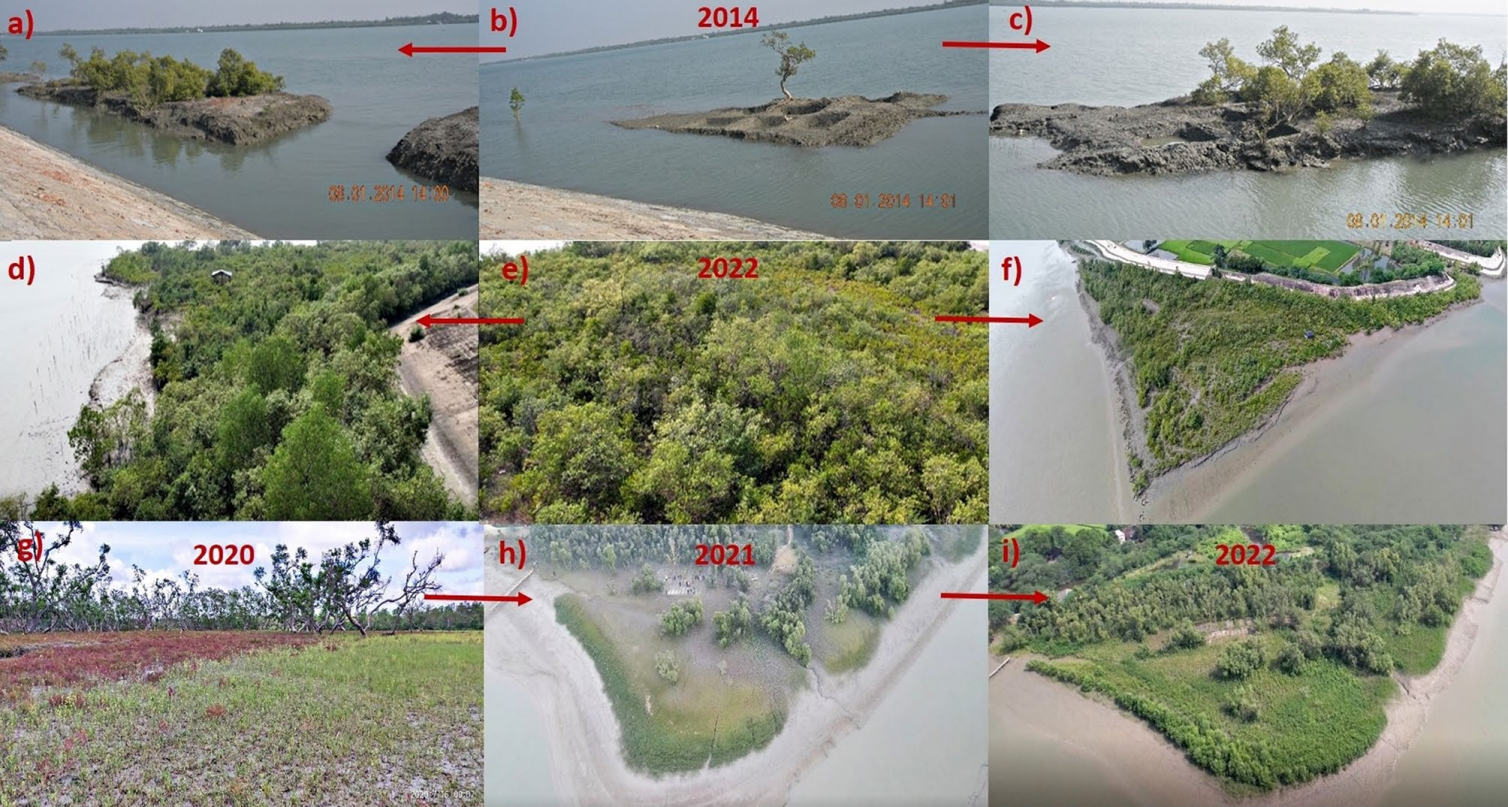
Ramganga semi-restored mangrove site and Durbachoti, one ongoing mangrove restoration site, each at different time points (**a**–**c**) Ramganga in 2014, before the initiation of restoration activities just in front of newly constructed dyke (**d**–**f**) Ramganga in 2022, ecologically semi-restored mangrove site shielding the old dyke (**g**) Durbachoti in 2020, before the initiation of restoration activities, (**h**,**i**) Durbachoti in 2021 & 2022 respectively, approaching ecological restoration.

Our all statistical analyses integrating the available data to date pointed out that this practiced restoration technology can be considered successful, now in 2023, for a small ~ 3 ha patch under restoration since 2014^37–43^ (Fig. 10a–f), while the other 30 small-area sites spanning ~ 62 ha in total is showing good progress for the last 2–3 years where the same framework is being replicated (Fig. 10g–i). The semi-restored ~ 3 ha patch at Ramganga village has been considered for potential OECM (Other effective area-based conservation measures) by OECM India (http://www.india-oecm.in/) in their internal database while other 30 sites at present are being developed to be in the same trail. Silvicultural objectives for generating ecosystem provisions for locals like terrestrial afforestation projects are little pertinent for Indian Sundarban deltaic mangroves, where erosion always outcompetes progradation, and saving mangrove belts from natural as well as anthropogenic stressors has become highly challenging in this region in this era of climate change^91^. Except for *Bruguiera gymnorrhiza* -dominated afforestation ventures, hardly any other restoration approaches were ever executed for re-creating the lost fringe mangrove habitats in Indian Sundarban settlement areas.

### Interaction among species across the stress gradient was utilized as a valuable guide for this restoration framework implementation

Based on our baseline observations during 2014–2022, we concluded that native halotolerant grasses (*Porteresia coarctata*, *Myriostachya wightiana*, *Sporobolus virginicus*, *Paspalum vaginatam*) perform the role of primary foundation species in lower-mid intertidal flats of our onsite mangrove niches^43,46^, with *Avicennia marina* and *Avicennia alba*, the most naturally abundant buffering species along with *Sonneratia caseolaris* as secondary foundation species. This assemblage once introduced onsite, exhibited immense potential towards downstream facilitatory cascades, enhancing accretion, accumulating nutrient-rich allocthonous sediments, trapping propagules, encouraging natural colonization by seedlings and epifauna, ameliorating physical stresses prevailing in the habitat, thus began reinstating multi-functionality in these degraded mangrove ecosystems at the initial successional stages.

The similar experiments by other mangrove biologists worldwide especially with halotolerant smooth cordgrass (*Spartina alterniflora*) and *Avicennia* spp.^24,48,49,51,52,92,93^validated our onsite observations. Similarly, the positive cues obtained from the studies of earlier researchers^50–52,58,94^ on increasing density and species-richness in mangrove niches led us to follow a dense spacing multi-species plantation style instead of the usual evenly spaced monospecies rows. This density-dependent facilitation of growth and survival seems more pronounced in mangrove communities because of the prevailing abiotic stresses in the environment, which corroborates the Stress Gradient Hypothesis (SGH)^95,96^, that the negative effect of stress can be relieved by spatially clustered organisms, finally resulting into their fundamental niche expansion.

At lower intertidal mudflats, halophytic grasses like *Porteresia coarctata* or *Avicennia* spp. seedlings survived the typical anoxic stress by colonizing in groups that could amplify the concentration of passively diffusing oxygen from their aerenchyma tissues in submerged roots and pneumatophores into their surrounding oxygen-deficient aquatic environment^96^. *Phoenix paludosa* (Fig. S6a–e) and the native halotolerant grasses exhibited strong conspecific facilitation^93^ (Figs. S1, S2, S6k) growing effortlessly in clusters at our sites of restoration as observed in natural mangroves of Indian Sundarbans. On the other hand, *Ceriops tagal*, *Ceriops decandra*, *Bruguiera cylindrica*, *Bruguiera gymnorrhiza*, *Excoecaria agallocha*, *Derris* spp., *Dalbergia spinosa*, *Sonneratia apetala*, *Bruguiera parviflora*, when planted in mixed fashion in our sites, yielded very successful establishment rates (Figs. S3.1, S3.2, S4, S5), an obvious outcome of multiple, independent, interspecific facilitation cascades in reassembling on-site mangrove community in degraded sites^51,52,58,92,93^. The introduction of mangrove legumes *Dalbergia spinosa* and *Derris* spp. in restoring species assemblage is expected to enrich onsite rhizospheric nitrogen from symbiotic diazotroph bacterial association in their roots.

The competitive exclusion is also strongly observed in monospecies plantations of *Bruguiera gymnorrhiza* across Sundarbans. Where they were planted alone, these species visibly obliterate the establishment of other species at the same site (Fig. 7a–e). Our observed extremely poor performance of co-located *Bruguiera gymnorrhiza* monospecies plantations in terms of species richness and density of natural recruits (Fig. 8a,b) accorded with the findings from Bangladesh Sundarbans 280 km^2^ *Sonneratia apetala* dominated monospecies plantations that even after 40 years demonstrated only ~ 38% species richness of natural mangroves (only 8 new species colonization after 40 years) via post-plantation natural recruits^20,27^. *Rhizophora* spp. and *Nypa fruticans* although flourish on the same sediment type, were found to be mutually exclusive at the same site, noticed repeatedly in our study. The salinity gradient of the restoration site from the river shore towards the upland in accord with the salinity tolerance potential of mangrove species^29^, was a crucial guide in designing our transplantation pattern (Fig. 9). The varying osmolyte accumulation trait in mangroves that offers osmotic tolerance was utilized by us as a contrivance to identify the ablest species to tolerate a particular onsite salinity level^30^.

### Microbial contribution as a growth promoter and restoration indicator in the present restoration approach

The concept of enriching native plant growth promoting rhizobacterial consortia in mangrove growth improvement and restoration programs was recommended by earlier researchers^97,98^. However, the applicability of native rhizobacterial consortia supplementation is difficult to validate for mangrove growth improvement, due to the highly dynamic diurnal tidal environment, where added microbiota even if added in a nursery environment (control), may lose out before they could enrich their community at onsite transplanted sapling’s rhizosphere. An important pyrosequencing study results established that dominant OTUs detected in the rhizosphere of nursery-grown *Rhizophora mangle* maintained their abundance in their rhizosphere even after 202 days of post-on-site transplantation^99^. This 202-day window is well enough for PGPR bacteria to promote the growth of transplanted seedlings at its very crucial early phase of establishment. We tested this PGPR application strategy with our three PGPR mangrove root endophyte bacterial consortia (Table S4) (out of 78 accessions of mangrove root endophyte pure bacterial isolates, MT421976–MT422053) on *Avicennia* spp. saplings and are presently extending this strategy to other target species.

A small database developed by us indicated a decrease in the abundance of Actinobacteria and Bacilli to be associated with restoration progress while a steady increase occurred in Planctomycetes abundance in restored surface sediments. Actinobacteria and Bacilli, both bacterial groups are ubiquitous among ecosystems. Actinobacteria-specific abundances are emphasized in resource-limited environments like less fertile farmland soils^100^whereas Bacilli can even thrive in mine substrates and therefore are suggested for their use in mine rehabilitation programs^101^. Planctomycetes possessing great glycolytic potential for plant-derived organic matter are well recorded to be signature bacterial communities of wetland habitats^102^ including Sundarban mangroves^103^. However, the abundance of Actinobacteria, Bacilli, or Planctomycetes was never utilized earlier as a metric for evaluation of wetland habitat restoration for achieving higher functional redundancy in native microbial communities as it advances towards reference pristine^101^.

### Mangrove pollination success as an indicator of restoration in the described framework

The use of indicators in this study measuring the diversity of pollinators, frequency of pollinator visits, and pollination success in restored mangroves in comparison to that of pristine references (Figs. S25–S28, Table S5), has not hitherto been exploited in the restoration evaluation of mangroves. This practice could be justified in the light of predominant entomophilous sexual reproduction in mangroves, the success of which is linked with the success of cross-pollination and pollinator activity. However, in the absence of a favorable ecological niche for outcrossing, their reproductive potential gets impaired^63–65^.

### Beginning of ecosystem services cited from the semi-restored site Ramganga

Our semi-restored and other ongoing restoration sites in the early stages at present have just begun to demonstrate traditional ecosystem services earned from restored mangroves in terms of livelihood generation (availability of near-shore native fishes and edible crabs)^23^ (Figs. S14, S15). However, as an instance of Nature Based Hybrid Solution (NBS)^81,85^, the semi-restored mangrove belt at Ramganga, referred to in this study happened to be located in front of hard engineering defense such as a landward dyke built in 2014 (Fig. 10a–d), and after our restoration execution, this hybrid site successfully restrained the obvious negative impact of two successive cyclones “Amphan” (20th May, 2020) and “Yaas” (26th May, 2021) that made landfalls very close to the experimental site^104^. The semi-restored mangrove site remained little damaged showing resilience to super cyclonic adversities; protected the concrete embankments and cultivated fields located in the upland (Fig. 10f), yielding coastal protection benefits to local inhabitants, which is more pertinent for mangrove restoration success from the highly vulnerable Indian Sundarbans’ perspectives.

### The present restoration framework is more useful for vulnerable mangroves

Overall this study emphasized on exploitation of site-specific strategies at an experimental scale with ecological rationales (Fig. 9), that are under-exploited in mangrove restoration to date and are successful in overcoming the constraints associated with mangrove restoration interventions, largely from the perspective of Indian Sundarbans, nonetheless, of some global relevance. All the results indicate the gradual return of functional independence of the experimental sites. We recommend this comprehensive restoration framework (Fig. 9) as a small-scale model for mangrove ecosystem restoration for highly vulnerable mangroves, facing primarily threats from erosion, salinity increase, and related anthropogenic stressors. The principles used in this framework (Fig. 9), are especially suitable for the recovery of stressed ecosystems that could be applicable also for such terrestrial forests/wetlands across the world, irrespective of their nature of degradation. Nevertheless, many-fold upscaling and multi-location trials of this established method across an array of site-specific stressors at similarly stressed mangroves of other parts of India and the world could only appraise the ubiquitousness and possible wider applicability of this present study.

## Methods

### Site description

31 small mangrove patches covering ~ 65 ha area, all located on the banks of Saptamukhi-Gobadia-Barchara-Mridangabhanga rivers at the Western part of the Indian Sundarbans delta, represent the ongoing restoration sites where the present restoration framework was applied including the semi-restored site at Ramganga village, Patharpratima Block, South 24 Parganas, West Bengal (21°47′32.10″ N, 88°22′ 57.30″ E) (Figs. 1, 10, Supplementary Data S1). In addition, another 13 degraded fringe mangrove sites, 2 mono-species plantation sites, and 4 pristine mangrove reference sites, all co-located, have been referred to in the study (Fig. 1, Supplementary Data S1). Location coordinates and areas of all the referred sites are included in Supplementary Data S1. Extensive baseline data from all the referred mangroves, and chronological data at time intervals have been developed from the present semi-restored site at Ramganga in 2014, 2016, 2021, and 2022 for 25 quantifiable variables used in the study (Supplementary Data S1). Chronological Google Earth images of the semi-restored site at Ramganga are provided in Supplementary Information (Fig. S13).

### Prime components of restoration framework

Five major components were used along with some special site-specific strategies (Supplementary Information). These are (a) grass-assisted stabilization with four native halophytic grass species viz. *Porteresia coarctata*, *Myriostachya wightiana*, *Sporobolus virginicus*, *Paspalum vaginatam*, planted at the lower and middle intertidal mudflats spanning high (~ 23–24%), medium (~ 9–11%) and low (~ 4%) cover, executed at the onset of restoration activities (Figs S1, S2, S12); (b) multispecies assemblage (Figs. S3.1,2, S4, S5) with 28–33 native true mangrove and mangrove associate species (Tables S1, S2, S3) was carried out via human-assisted transplantation in large scale, following the species composition, assemblage pattern and successional trend from reference pristine mangroves, after pre-establishment at onsite nurseries or through direct seeding; plantation pattern based on physiological continuum of salinity tolerance of species (indicated by osmolyte accumulation level) and the gradient of salinity prevailing at the site; (c) facilitative interaction where successive transplants were spaced at very close proximity to each other (≤ 5 ≤ 40 cm gap), with high density, in clumps/clusters, rather than conventional spread pattern (≥ 100 cm gap); large scale experimentation implemented in this regard with locally threatened mangrove species like *Phoenix paludosa*, *Heritiera fomes*, *Lumnitzera racemosa*, *Nypa fruticans*, *Xylocarpus mekongensis*, *Brownlowia tersa* (Fig. S6); (d) application of onsite PGPR consortia in 3 combinations (BC1, BC2, BC3) comprising of 19 best native mangrove root endophytic pure bacterial isolates (Table S4, Figs. S10, S19–S21). Two doses of bacterial consortia were applied at 39 days’ interval on 28-day-old nursery-established seedlings of *Avicennia* spp. (Fig. S10); (e) seed ball use strategy (Fig. S7) where seeds of *Phoenix paludosa, Heritiera fomes,* and *Brownlowia tersa* are embedded in the less-saline muddy sediments (EC < 1 dSm^−1^), dried under shade to make small balls containing 2–10 seeds, and later dispersed onsite (Table S7). In addition, other local site-specific strategies followed were channel grooving for improving hydrology (Fig. S8), transplantation of seedlings in wired cages (Fig. S11) for most erosive shorelines, and indigenous method of collecting seeds of locally rare species located at far-off fragmented niches (Fig. S9).

### Field-based studies for baseline data collection

Baseline vegetation data were collected on species composition, pattern of assemblage, species richness, mangrove structure viz. species-specific diameter at breast height (DBH), tree basal area (TBA), relative density, frequency, dominance, importance value index (IVI), cover%, Shannon’s diversity index from all the referred 31 mangrove patches of ongoing restoration sites including the presently semi-restored site at Ramganga at 2014, 2016, 2021 and 2022, 13 additional degraded fringe mangrove sites, 2 mono-species plantation sites, and 4 pristine mangrove reference sites at time intervals (Supplementary Data S1, Fig. S18). Quantitative data on species richness and density of naturally regenerated seedlings (including the regenerated ones from the trapped propagules in the onsite planted native grass community) and identified epifaunal members were estimated at different time intervals with multiple replicates (Supplementary Data S1). Estimation of the reproductive success of mangrove species was obtained by artificial breeding experiments set at different time points during the flowering seasons of 2014–2022 across the referred mangroves. On the day of the anthesis, ~ 400 flowers per mangrove species per season were selected for autogamy, geitenogamy, xenogamy mode of pollination and later monitored for initiation of fruit set. The fruit set% was designated as self and cross-pollination success. To determine the pollinator abundance, 5 m^2^ canopy quadrats having diverse mangrove species were located randomly and the abundance of pollinators per m^2^ canopy areas was counted during the flowering seasons. Data from different observations were pooled for each mangrove species for each type of visiting pollinator insect and averaged for expressing the pollinator abundance per m^2^ per hour. Pollinators are identified to estimate the species richness from the semi-restored site Ramganga during 2014–2022 (Supplementary Data S1, Supplementary Information Table S5).

### Soil sampling

Sediment cores (60 cm long and 4 cm wide) were collected from a depth of 0–15 cm and 45–60 cm from 25–40 points across the different mangrove sites referred to above covering 5–10 random quadrats of 10 m × 10 m and brought to the laboratory keeping in an icebox. A part of the soil was stored at 4 °C for soil microbial enzyme assay and bacterial CFU count (from 0 to 15 cm deep cores only), sulfide estimation (from 45 to 60 cm deep cores only), rest parts were air dried at room temperature (28 °C) for soil physical criteria evaluation, nutrient profiling, and next generation sequencing (from 0–15 cm deep cores only).

### Laboratory-based analyses

Reagents were purchased from companies like Sigma-Aldrich, Merck, and Himedia. Physical analyses of sediment conductivity and pH were measured using the method of saturated extract preparation in the proportion of 1:2 (w/v, air-dried sediment: deionized water)^105,106^. Conductivity was measured with a conductivity meter (Chemiline CL250, Labline Technology Pvt. Ltd. Ahmedabad, India) and was expressed in dSm^−1^. The pH was determined using a pH meter (Chemiline, Labline Technology Pvt. Ltd., Ahmedabad, India). Soil texture determination of sand%, silt%, and clay% using different pore-sized sieves^107^ was carried out separately. The biochemical analyses were conducted for ammonia-N, organic carbon, soluble phosphorus, sulfide-sulfur, and arylsulfatase activity using spectrophotometric methods. Ammonia-N detection was done by extracting 2 g of sample with 2 M KCl, using the phenate method^108^. After 1 h, the developed indophenol blue colour^108–110^ was measured with a double beam spectrophotometer (Shimadzu) at 640 nm. Organic carbon^111^ was determined by reacting 1 g of sediment with 1/6 M potassium dichromate (K_2_Cr_2_O_7_) and concentrated sulfuric acid containing 1.25% silver sulfate (Ag_2_SO_4_) forming a green color when incubated for 30 min., and the color intensity was measured at 660 nm. To detect plant-available phosphorus, 2 g of air-dried soil sample was extracted with modified Morgan extractant^112^ and tested for soluble phosphorus by the molybdenum-blue method^113^ at 660 nm. For sulfide-sulfur assays, 2.5 g of fresh sediment was reacted with phosphoric acid to liberate the sulfide by steam distillation following the addition of DPD sulfuric acid reagent, potassium dichromate solution; the absorbance of the resulting blue color was measured at 670 nm to estimate the sulfide-sulfur content^114^. Arylsulfatase activity of soil microbes was measured using 0.5 g of air-dried soil extracted with 0.5 M acetate buffer and 0.05 M para-nitrophenyl sulfate solution and quantified against para-nitrophenol as standard at 400 nm^115^. Colony-forming units (CFU) of different nutrient-cyclers like cellulose degraders^116^, free-living nitrogen fixers^116^, phosphate solubilizers^117^, iron oxidizers^118^, ammonifiers^119^, nitrifiers^120^, nitrite oxidizers^120^, aerobic denitrifiers^121^, sulfur oxidizer^116^ bacteria were recorded by spreading serially diluted sediment samples in above mentioned nutrient specific differential agar-based media.

For plant growth promotion (PGP) attributes evaluation of rhizospheric bacterial isolates, IAA production assay was performed by Salkowski reagent method^122^ using 0.5 M FeCl_3_ solution and 35% perchloric acid in reaction, followed by pink colored IAA complex and quantified at 530 nm via spectrophotometry. The phosphate solubilization activity was followed by soluble phosphorus estimation via molybdenum-blue method^113,117^. Quantitative estimation of secreted siderophore^123^was followed by universal chrome azurol S (CAS) assay method measuring absorbance at 630 nm. ACC deaminase assay was performed by colorimetric 2,4 -dinitrophenyl hydrazine assay method at 540 nm using spectrophotometry^124–127^. Free-living nitrogen-fixing bacteria were stringently selected for each bacterial isolate on Jensen's Medium^128^ and analyzed for acetylene reduction assay^129^ by gas-chromatography method (6000 series Gas Chromatograph System with Agilent HP-5 Capillary column) against a standard of ethylene gas.

Osmotic acclimation was assessed by spectrophotometric assay for glycine-betaine carried out at 365 nm^130^ using 1, 2 dichloroethane as internal blank based on the fact that at low-temperature betaine makes a betaine-periodite complex with iodide in acidic medium. The soluble sugar and starch^131^ was quantified from air-dried leaf samples where in a hot acidic medium soluble sugars were dehydrated to hydroxymethyl furfural forming a yellow-orange color with phenol that had an absorption maxima at 490 nm.

For Next Generation Sequencing of the V3-V4 region of the 16S ribosomal rRNA gene, the genomic DNA was extracted from soil samples using commercially available kits (Nucleospin soil). The isolated metagenome DNA samples were quantified using Nanodrop at 260 and 280 nm and 2 × 300 bp MiSeq libraries were prepared using Nextera XT Index Kit (Illumina Inc.). The QC-passed libraries were sequenced on the Illumina MiSeq platform to generate FASTQ files for further bioinformatical analyses.

### Bioinformatics

All NGS metadata and 16S gene sequences of pure PGPR bacterial isolates are accessioned in NCBI (Supplementary Information, Tables S4, S12.1,2). The metabarcoding analyses were carried out by using DADA2 pipeline^132^ in R program version 4.4.2. Paired end FASTQ files have been demultiplexed and adapters have been removed. After visualizing the quality profile, sequences were trimmed at position 20 in both the forward and reverse reads. After removing the chimeric sequences the ASV sequence table was created followed by taxonomy assignment using “silva_nr99_v138.1” with a minimum bootstrap value of 80.

### Statistical analyses and software used

Statistical analysis with subsequent graphical interpretations was carried out using R programming language (version 4.4.2), SPSS (version 23), SigmaPlot (version 14.0), and GraphPad Prism (version 8.0.1). Map-based illustrations for denoting geographical locations were executed through open access QGIS (version 3.28.3) (https://qgis.org/en/site/forusers/download.html) and Google Earth images were exported from open access Google Earth Pro (version 7.3.6.9750, 64-bit) (https://www.google.com/intl/en_in/earth/about/versions/#download-pro). Four advanced statistical codes were programmed viz. Ridgeline plots^69^, Kolmogorov–Smirnov (K-S) tests^70,71^, Mahalanobis’s D-square measures^72^, and Bayesian t-tests^68^ to validate the success of our restoration technologies applied, whether, over time, the degraded mangrove niches are gradually approaching towards the pristine condition or not. The distribution of 25 quantifiable variables was checked across 6 different ecological states of mangrove niche, viz. Degraded, Ramganga 2014, 2016, 2021, 2022, Target pristine. Here Ridgeline plots for all the 25 variables over different ecological conditions of mangrove stands displayed their respective distributions. For each variable, 5 Kolmogorov–Smirnov (K-S) tests were performed pairwise in between its distribution in pristine and each of those other 5 mangrove ecosystem categories. In Mahalanobis Distance (D^2^), the distance measure was computed for each of all sampling units under 5 different states based on the following equation:

$$D^{2} = (x - m)\prime \sum^ {-1} (x - m)$$

where x refers to a vector of 13 observations representing each of 13 chosen variables from 5 different mangrove niche states, m denotes the vector of mean values of those 13 variables in Pristine, and Σ^(−1) denotes the inverse of the variance–covariance matrix of the 13 variables in Pristine. Redundancy analysis (RDA)^55,67^ was conducted with sets of response variables that can be explained by a set of explanatory variables performed in XLSTAT 19 software and represented in biplots. To determine the best possible regression model for explanatory and response data sets Bayesian t-test was performed with “Bolstad” package (version 0.2–41) in R program. To analyze and visualize the 16S rRNA amplicon data, deduced from the FASTQ files “ampvis2”^133^was used, which is an R package designed for the analysis of microbial community data focused on metadata integration. For visualization of bacterial abundance (%), the “amp_heatmap” and “amp_boxplot” functions were used.

The mean value ± standard error was determined for all experimental data analyses considering a minimum of 10 biological replicates each with 3 technical replicates, wherever possible. Analysis of variance (ANOVA) (Table S11) along with Tukey’s honest significant difference (HSD) test were used in SPSS 23.0 to determine significant differences at the 5% confidence level across the values measured which were designated with different letters. SigmaPlot 14.0 was used to prepare the bar diagrams.

### Plant seeds and propagules collection guidelines, permissions, and voucher specimens

The use of plant seeds and propagules in the present study complies with international, national (Govt. of India), and Institutional guidelines. All the plant seeds and propagules used in the plantation-based field experiments are native and were collected from different islands of the Indian Sundarbans with due permission from the Directorate of Forests, Govt. of West Bengal. The voucher specimens of these materials have been deposited (No. 057411) in a publicly available herbarium named Central National Herbarium (Herbarium code CAL), Botanical Survey of India. The on-field identification of the plants was carried out by Dr. Sandip Kumar Basak, Plant Taxonomist and co-corresponding author of the manuscript and later authenticated by Scientist-in-Charge, Central National Herbarium, Botanical Survey of India.

### Supplementary Information

Supplementary Information 1.

Supplementary Information 2.

## Data Availability

All data generated in the current study are present in the main text and uploaded to the Electronic Supplementary Information and Supplementary Data 1 files.
