# Supplementary material for: Ecological restoration at pilot-scale employing site-specific rationales for small-patch degraded mangroves in Indian Sundarbans
Source: Sci Rep. 2024 Jun 5;14:12952. doi: 10.1038/s41598-024-63281-8 (PMC11153218; doi:10.1038/s41598-024-63281-8)
Supplement: Supplementary file 2 — Supplementary Information 2. [file 41598_2024_63281_MOESM2_ESM.pdf]

Supplementary Information

Ecological restoration at pilot-scale employing site-specific rationales for small-patch degraded mangroves in Indian Sundarbans

Krishna Ray[1*], Sandip Kumar Basak[2*], Chayan Kumar Giri[1ǂ], Hemendra Nath Kotal[1ǂ], Anup Mandal[1ǂ], Kiranmoy Chatterjee[3ǂ], Subhajit Saha[1ǂ], Biswajit Biswas[1], Sumana Mondal[1], Ipsita Das[1], Anwesha Ghosh[4], Punyasloke Bhadury[5], Rahul Joshi[6]

[1]Environmental Biotechnology Group, Department of Botany, West Bengal State University, Berunanpukuria, Malikapur, Barasat, Kolkata 700126, India

[2]Sarat Centenary College, Dhaniakhali, Hooghly 712302, West Bengal, India

[3]Department of Statistics, Bidhannagar College, Salt Lake City, Sector 1, Block EB, Kolkata 700064, India

[4]Centre for Climate and Environmental Studies, Indian Institute of Science Education and Research Kolkata, Mohanpur-741246, Nadia, West Bengal, India

[5]Integrative Taxonomy and Microbial Ecology Research Group, Department of Biological Sciences, Indian Institute of Science Education and Research Kolkata, Mohanpur-741246, Nadia, West Bengal, India

[6]Zoological Survey of India (ZSI), Prani Vigyan Bhawan, Block M, New Alipore, Kolkata 700053, India

[*]Corresponding authors

[ǂ] Authors contributed equally to this work

[*]Corresponding authors' email addresses:

kray91@gmail.com, sandipbasak9592@gmail.com

ORCID ID: KR, 0000-0002-1893-5993; SKB, 0000-0001-7602-5851

Supplementary Information

The followed key objectives of the ecological mangrove restoration framework described in the study:

Objective 1:

On-site application of an experimental restoration framework at smaller scales that integrates site-specific scientific rationales, both conventional and non-conventional components

Grass-assisted stabilization:
The degraded sites of restoration was initially stabilized by large scale planting of four native salt tolerant grasses that are indigenous to Indian Sundarbans such as *Porteresia coarctata*, *Myriostachya wightiana*, *Paspalum vaginatum* and *Sporobolus virginicus*. *Porteresia coarctata* was utilized exclusively for lower intertidal zone stabilization, whereas *Myriostachya wightiana*, *Paspalum vaginatum* and *Sporobolus virginicus* were exploited for middle and upper intertidal flat stabilization (Figure S1). Grasses have undergone several vegetative cycles through their runners and stolons. Planted native halophytic grasses encouraged on-site trapping of mangrove propagules in large numbers (Figure S2), improved the soil textures, nutrient profile by encouraging enrichment by nutrient cycling bacterial community harboring the grass rhizosphere, thus assisting greatly in restoring the degraded mangrove landscape.

Multispecies plantation strategies/activities/pattern in detail:
Overall, 24-29 species of mangroves and associates viz. *Bruguiera parviflora*, *Bruguiera gymnorrhiza*, *Bruguiera cylindrica*, *Rhizophora mucronata*, *Ceriops decandra* (near threatened), *Ceriops tagal*, *Aegialitis rotundifolia* (near threatened), *Aegiceras corniculatum*, *Heritiera fomes* (endangered), *Xylocarpus mekongensis*, *Excoecaria agallocha*, *Acanthus ilicifolius*, *Acanthus volubilis* (locally threatened), *Merope angulata* (locally threatened), *Derris trifoliata*, *Brownlowia tersa* (near threatened, *Avicennia marina*, *Avicennia officinalis*, *Avicennia alba*, *Dalbergia spinosa*, *Phoenix paludosa* (near threatened), *Sonneratia caseolaris*, *Sonneratia apetala*, *Nypa fruticans* (locally rare), *Intsia bijuga* (vulnerable), *Lumnitzera racemosa* (locally threatened) were multiplied at the onsite-nursery
At our degraded sites, we have transplanted four halophytic grass species: *Porteresia coarctata*, *Myriostachya wightiana*, *Sporobolus virginicus*, *Paspalum vaginatum*. Individual grasses have their own ecological preferences like *Porteresia* inhabits the lower mudflat area. *Myriostachya* and *Sporobolus* prefer the mid and upper intertidal zones, *Paspalum* always grows towards the upland of the mangroves.
Firstly, we transplanted the grasses according to their zone of preference to stabilize the degraded landscape. After their well establishment, the grasses themselves make the soil nutrient-rich. In degraded mangrove areas, erosion is higher than sedimentation/progradation. Sedimentation is a regular process following grass transplantation and its establishment. These nutrient-rich newly deposited soil sediments help transplanted mangrove seedlings to grow better. In a dense population of grasses, the mangrove seeds are trapped and the natural succession of mangroves is facilitated based on the physiological continuum of salinity tolerance. A set of species with high salt tolerance potential (high osmolyte accumulators) and capable of withstanding the high tidal force/high wind speed, were utilized as the front liners in shore-line zones, earlier stabilized with halophytic grass *Portersia coarctata*. A second set of species was utilized for planting next to the shoreline zone that seemed to be fit for growing in the medium saline zone with lesser tidal insurgence (moderate osmolyte

accumulators). This zone was also stabilized with the grasses, *Myrostachya wightiana* and *Paspalum vaginatum* that can flourish at moderate salinity and moderate tidal pressure. The back mangrove area in the zone of low salinity was transplanted with less osmolyte accumulators and grasses like *Paspalum vaginatum* and *Sporobolus virginicus* (see Table S1 for species details). This pattern of transplantation corresponded with the zonal distribution of mangroves in neighborhood pristine (least disturbed) mangrove forests (used as reference sites) in Indian Sundarbans (Figure S3.1, S3.2, S4, S5, Table S1-S4).
Successive transplants were spaced at very close proximity to each other, at high density rather than the conventional spread pattern (Figure S6). In pristine references, naturally regenerated seedlings are usually observed to remain densely crowded at the young phase followed by later thinning out due to competition.

Transplantation via direct seed/propagule sowing:
Phoenix paludosa seedlings when transplanted individually like other mangrove saplings based on the soil salinity gradient of the site, have a very low survival percentage below 10% was observed. However, when 10-15 direct seeds in clumps were planted, *Phoenix paludosa* showed 70% survival with better growth. Similarly, direct seed/propagule sowing for *Heritiera fomes*, *Lumnitzera racemosa*, *Xylocarpus mekongensis*, *Ceriops tagal*, *Ceriops decandra*, *Bruguiera gymnorrhiza*, *Bruguiera cylindrica*, *Bruguiera parviflora*, *Aegiceras corniculatum*, *Aegialitis rotundifolia*, bypassing the need for nursery maintenance during their fruiting seasons accompanied by large availability of propagules, saved the time and cost for restoration and yielded comparable survival percentage with that from transplanted nursery-maintained seedlings. Usually, the seeds or propagules when directly planted, were planted 6-8 cm deep in the mid-upper intertidal zones of the sites, so that the tidal current could not wash them off.

Microbial involvement in the restoration process as the application of plant growth promoting bacterial consortia:
Root and pneumatophore samples of different mangrove species were collected in sterile bags maintaining 4°C from the Western part of Indian Sundarbans. For isolation of the endophytic bacteria, sliced samples were surface sterilized with 0.1% $HgCl_2$ solution followed by inoculation and enrichment into Luria-Bertani broth with subsequent repeated stringent selection on differential nutrient cycling media. Based on the observation of comprehensive plant growth promotion (PGP) profiles of all the isolated 78 mangrove root endophytic bacterial strains, the best 19 bacterial strains were selected for preparing 3 different combinations of large-scale bacterial culture for the application on the rhizosphere of *Avicennia* spp. seedling beds. Individual bacterial member from each of the combinations is initially inoculated in small culture of 100 ml of Nutrient Broth (conductivity ~8-9 dSm^{-1}) and then raised to 600 ml of large volume with final OD 2-3 in 48-72 hrs. Finally, each of the large-volume cultures was mixed to prepare the above-mentioned consortia. For *Avicennia* spp. 4 liters of culture volume was prepared with each bacterial combination to be applied after 3 times dilution with river water. Here 80 *Avicennia* seedlings were used for each consortium. 50 ml of diluted culture was applied to each seedling of *Avicennia* spp. keeping a set of controls (no consortia added). The three different bacterial consortia BC1, BC2, and BC3 were applied after 28 days of sowing *Avicennia* spp. seeds and 2nd dose of consortia addition were executed after 39 days of 1st dose of consortia addition (Figure S10).

Improvement of hydrological conditions-a site-specific strategy followed:
This technique is crucial to the success of site-specific ecological restoration. Different degraded mangrove sites face different levels of hydrological forces/hindrances, based on the

site's proximity to the river or ocean, the disturbance caused by humans, and the artificial wave created by steamer boats. Steamer boat-generated waves in the river caused high-level erosion at one of our degraded ongoing restoration sites. Transplanted seedlings were severely damaged by the artificial waves falling onto the riverbank.

We planted thousands of bunches of grasses and mangrove seedlings repeatedly, however they failed to survive at this high level of disturbance. It was observed that their maximum mortality is due to the erosion of soil from the rhizosphere of the transplanted seedlings. The root system got exposed and it lost the mechanical support and subsequently collapsed. Sedimentation is a regular process here, but erosion outcompeted natural sedimentation for this vulnerable site.

28 channel drains were dug in the lower mudflat of the restoration site horizontally to the river, 0.6 m deep and about 200 m long (Figure S8). The drains were then left for a month to get filled with sediments received from tidal inundation. *Avicennia alba*, a locally dominated mangrove species that prefers to grow in lower mudflats, were transplanted along with halophytic grasses *Porteresia coarctata* and *Myriostachya wightiana* after sediment deposition.

Avicennia spp. are found to dominate the Indian Sundarbans with their natural colonization right at the lower intertidal flat along with *Porteresia coarctata* grass community, and its growth rate is much higher than any other mangrove species, inhabiting this local mangrove ecosystem. In the lower mud-mudflat area, we aimed to create a natural barrier of *Avicennia* spp. to prevent the waves generated by steamers from hitting directly on river banks causing the planted species to uproot even they were planted based on site-specific, species-specific "windows of opportunity". The ongoing restoration sites where the maximum mortality of the newly planted seedlings is due to the massive erosion of soil, we have used this hydrological improvement technology.

A special site-specific indigenous method to avoid the erosional force uprooting the planted seedlings:

A special restoration technique was followed for very erosion-prone mudflat areas of restoration. The vulnerable eroded cut edges of the restoration site facing most of the erosive transportive tidal force were transplanted with seedlings protected by iron-wired cages or inserted in bamboo segments at the root zone (Figure S11) purposely so that the sediments confined within the cage/bamboo cover protect the roots till the establishment of the seedlings and do not let it washed away by tidal current. This technique was applied in the Ramganga semi-restored site where the iron cages or bamboo supports with seedlings inside them were pressed into the mud, the transplanted seedlings were in upright condition, the iron wired cage/bamboo anchored the soil and gave the seedlings mechanical strength during their very young stage of establishment.

Seed-ball use technology applied for rare and threatened (RET) mangrove species:

Seed ball is a technique that accelerates the mangrove rehabilitation process in sites with limited access, human resources, and infrastructure or uninhabited areas using UAV, unmanned aerial vehicle (Arifanti et al., 2022). The earlier report from Indonesia, where most used seeds in seed balls used in the Integrated Mangrove Sowing System (IMSS) were *Avicennia* spp. and *Sonneratia* spp. based on their abundant availability and continuous availability throughout the seasons. It is a cost-effective way to restore mangroves to avoid the expensive affair of seedling maintenance at the nurseries. For estuarine mangrove ecosystems where tidal fluctuations are a regular occurrence, the establishment of mangrove seedlings through seed ball technology is much more challenging. As a result of the tidal current during high tide, seed balls have a high chance of getting washed out after dispersion.

Therefore, after the dispersal of seed balls, they are manually pressed into the mud in deep so that the balls cannot be washed out. A constant vigil at the sites at the locations where seed balls are planted prevented the seed balls from being washed out, and finally, seedlings emerged.
The following steps were followed to prepare the ball: 1) the seeds are embedded within the clayey less saline soil (EC<1dSm^{-1}), 2) the balls are then dried under shade, and 3) the seed balls are dispersed in the restoration sites. The seed balls of rare and near-threatened species were targeted only in this intervention. Seed balls of *Heritiera fomes*, *Phoenix paludosa*, *Brownlowia tersa* were utilized in this restoration work. Due to the availability of a large number of seeds of *Phoenix paludosa*, in one particular season led us to use the excess seeds that could not be utilized for sapling generation in nurseries due to scarcity of less saline soil (EC<1dSm^{-1}), and we used 6-10 seeds per ball for this *Phoenix paludosa*, 2-5 seeds for *Brownlowia tersa* and 1-2 seeds for *Heritiera fomes* per seed ball prepared. The overall survival rate was found to be about 44.44% among which *Phoenix paludosa*, showed the highest survival rate (58.18%) (Figure S7, Table S7).

Objective 2:

Use of a set of quantifiable indicators (conventional as well as innovative, some typical of local mangroves) to evaluate restoration success at the early phase of re-organization (within 2-6 years) with time, in comparison to co-located reference pristine mangroves and old monoculture mangrove sites.

Restored mangrove structure and species composition as an indicator of restoration:
To study the mangrove niche structure, (10×10) m^2 quadrat areas were randomly studied within the forest. The number of quadrats depends on the forest area. A minimum of 10 quadrats or more have been studied depending on the forest area. The circumference was measured at breast height (approximately 1.3 m above the ground) irrespective of tree and shrub species. DBH (Diameter at Breast Height) and TBA (Tree Basal Area) were calculated from the average circumference using the standard formula (Etigale et al., 2014). Within a 100 m^2 area, the number of species and their individuals were counted. The total tree basal area (TBA) for a particular species is obtained by multiplying the number of individuals with TBA for that particular species in a single quadrat. Then the calculation of the total basal area for each quadrat was carried out by summing up all the TBA of the species present in that quadrat. TBA is represented in m^2 ha^{-1}.

Edaphic factors, physical and biochemical, as a metric of restoration:
Sediment samples from 0-15 cm depth and 45-60 cm depth from 20-40 points across the experimental sites were collected and brought to the laboratory keeping in the icebox. A part of the soil was stored at 4 °C for enzyme activity (assayed from 0-15 cm cores) and sulfide estimation (from 45-60 cm cores only) with the rest portion air dried at room temperature (28°C). All estimations were carried out from different undisturbed pristine mangroves as a reference, degraded sites, monospecies sites, and sites of restoration. The biochemical analyses were conducted for NH_4-N, organic carbon, soluble P, sulfide-sulfur, soil enzymes like CMC-cellulase, β-glucosidase, aryl sulfatase, urease, phenol oxidase, alkaline and acid phosphatase and physical analyses like conductivity and pH estimation along with soil texture determination by sand, silt, clay percentages were carried out.

Genomic abundance and density profile of nutrient-cycler microbiota in sediment as the metric of restoration:
Sediment samples were collected from 20-40 points across the experimental sites, pristine and degraded mangroves from 0-15 cm depth and then made dry and mixed into single composite sample. The genomic DNA was extracted from these soil samples and next generation sequencing using Illumina MiSeq platform for determining absolute abundance of 16S rRNA gene sequences for experimental site soil habitats. Sediment samples from 0-15 cm depth from 20-40 points across the experimental sites along with pristine mangroves as reference model and degraded sites were collected and brought to the laboratory maintaining 4^{0}C. Colony forming units (CFU) of different nutrient cyclers like cellulose degraders, free-living nitrogen fixers, phosphate solubilizers, iron oxidizers, ammonifying, nitrite oxidizing, aerobic denitrifiers, sulfur oxidizer bacteria were recorded.

Osmotic acclimation of mangrove species as indicator of restoration:
All osmolytes (compatible solutes) like proline, glycine-betaine, soluble sugar and starch, free inositol, and total free amino acids were estimated using spectrophotometric methods from leaves of existing plant species inhabiting the experimental sites as well as reference pristine and degraded mangroves.

Reproductive/pollination success, pollinator diversity, and frequency of visits as a metric of restoration:
At experimental sites as well as at different reference pristine and degraded mangroves, ~400 flowers per existing mangrove species per season was selected for artificial breeding experiments at different time points of the flowering seasons (2014-2022). Out of these ~400 flowers, some were bagged for selfing (autogamy), rest were emasculated and pollinated with the pollen of flowers on the same plant (geitenogamy), and also pollinated with pollen of a different flower on a different individual plant (xenogamy) and later bagged and followed for initiation of fruit set. The percentage of fruit set was calculated for each pollination mode as success of pollination.
At the experimental site along with pristine reference and degraded sites, 5 m^2 canopy quadrats of different mangrove species were located randomly and the abundance of pollinators per m^2 canopy areas was counted. Visitors to each plant species were counted on different days during the flowering season from 2014-2022 between 6-10 AM because this was found to be the time of maximal visitation of pollinators. Any single pollinator entering every time in the selected area was counted as a new arrival (so it may be possible that in a few cases individual insect was counted more than once). Data from different observations were pooled for each mangrove species for each type of visitor insect and averaged for expressing the pollinator abundance per m^2 per hr.

Epifaunal diversity and abundance as a metric of restoration:
A minimum of 10 quadrats of 1 m^2 were studied from pristine, degraded, and semi-restored mangroves, mainly at lower intertidal habitats to study the epifauna. Crab burrows, mudskipper burrows, mollusks, hermit crabs, and crabs, the major epifaunal species, and their abundance were counted in each quadrat. The number of species and abundance is represented per hectare of area for each habitat.

Quantification of post-plantation naturally recruited seedlings as an indicator of restoration:

Natural regeneration of seedlings from randomly laid (10×10) m^2 quadrats from different mangrove habitats was studied. As seed dispersal of mangroves mainly occurs through the tidal current and lower mudflat is the intertidal zone that favors regeneration the most, hence quadrat analysis was mostly executed in (10×10) m^2 areas randomly throughout the lower intertidal zones. We counted the no. of species and number of seedlings belonging to each species. Number of seedlings and number of species is represented in per hectare scale.

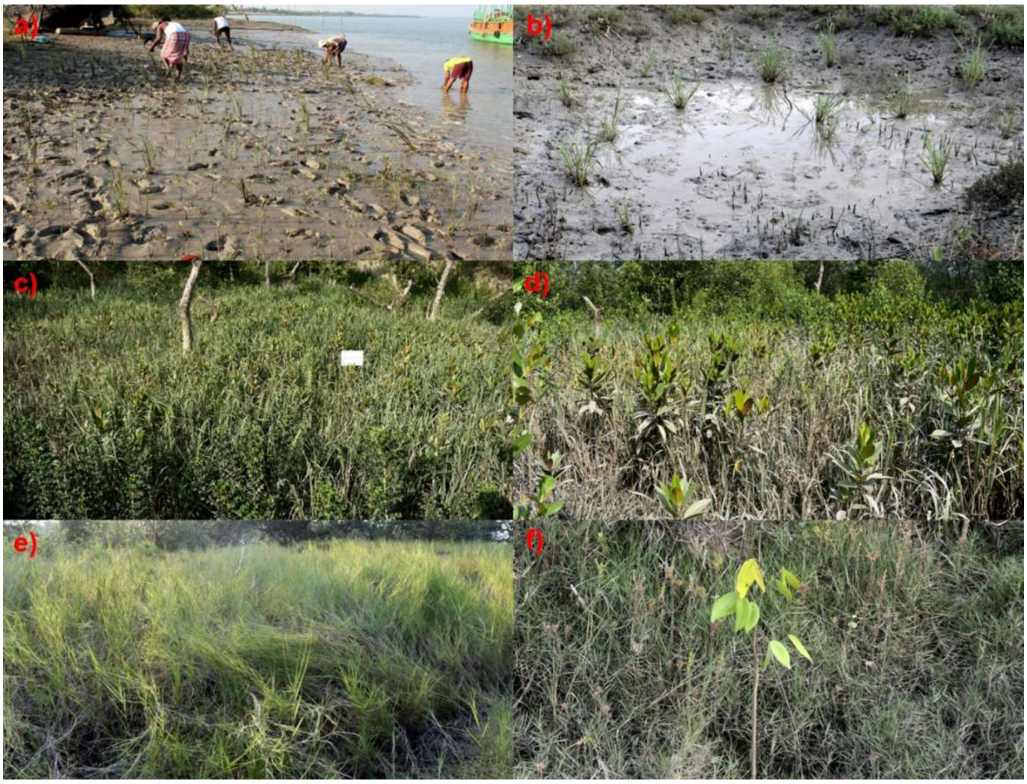

Supplementary Fig. 1 Images representing grass-assisted stabilization of restoration sites and its success a) Grass transplantation at the degraded sites b) Early phase of establishment of *Myriostachya wightiana* c) Very well established grasses at their best d) Well-developed transplanted mangrove seedlings within the dense grass population e) Well-established *Sporobolus virginicus* f) Well-established threatened mangrove seedlings within *Paspalum vaginatum*

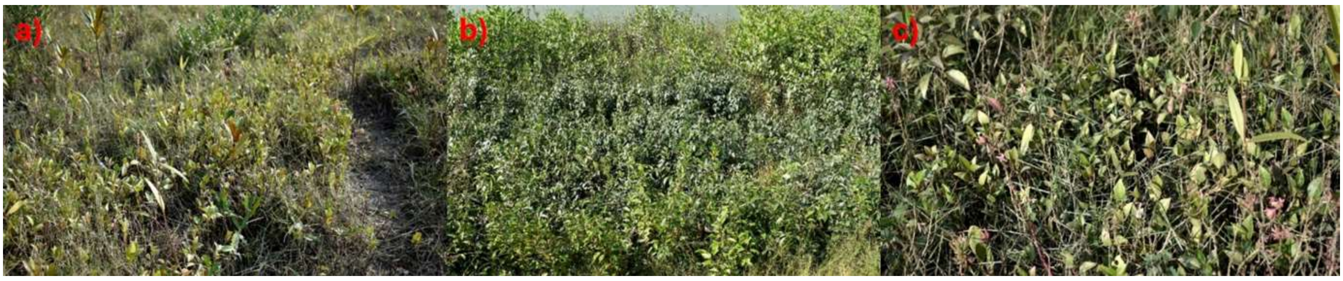

Supplementary Fig. 2 Images representing the benefits of grass transplantation. (a-c) Propagules are trapped by the established dense halophytic grass population at the different degraded ongoing restoration sites.

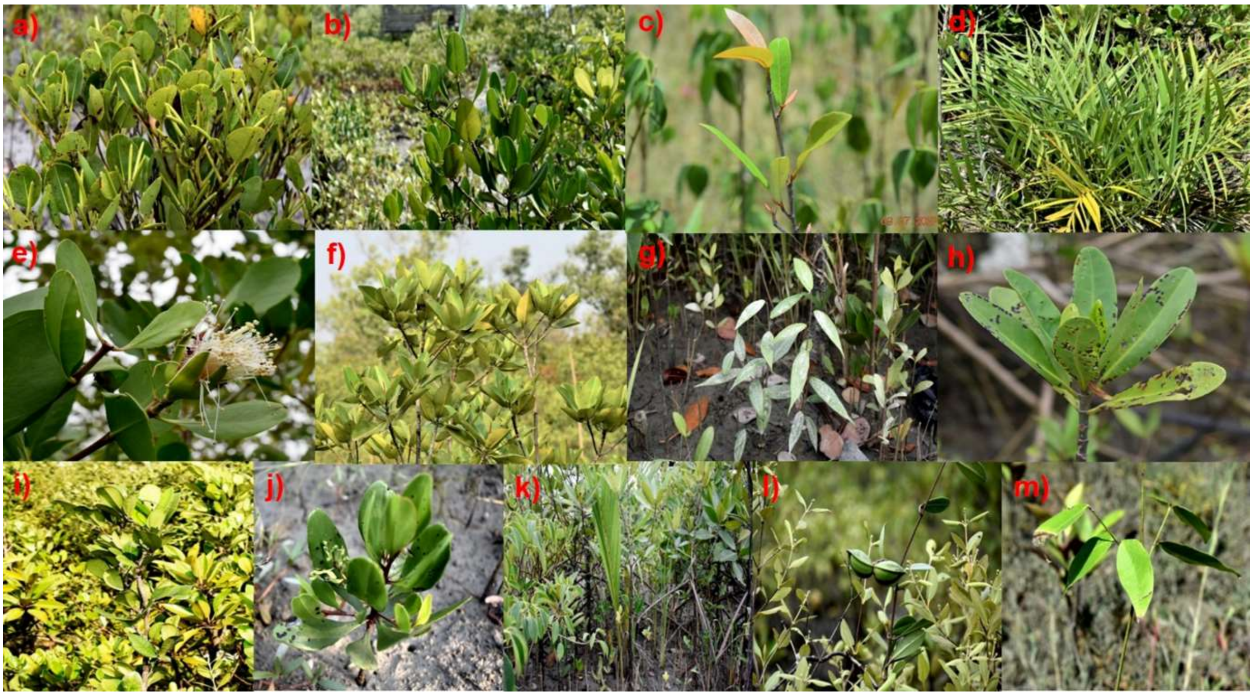

Supplementary Fig. 3.1 Images representing species-richness, some at early and some at mature phase of establishment from experimental sites of ongoing restoration activities along with the semi-restored site at Ramganga *a) Aegialitis rotundifolia, b) Ceriops decandra, c) Heritiera fomes, d) Phoenix paludosa e) Sonneratia caseolaris, f) Rhizophora mucronata, g) Brownlowia tersa h) Kandelia candel i) Xylocarpus mekongensis j) Lumnitzera racemosa k) Nypa fruticans l) Finlaysonia obovata m) Intsia bijuga*

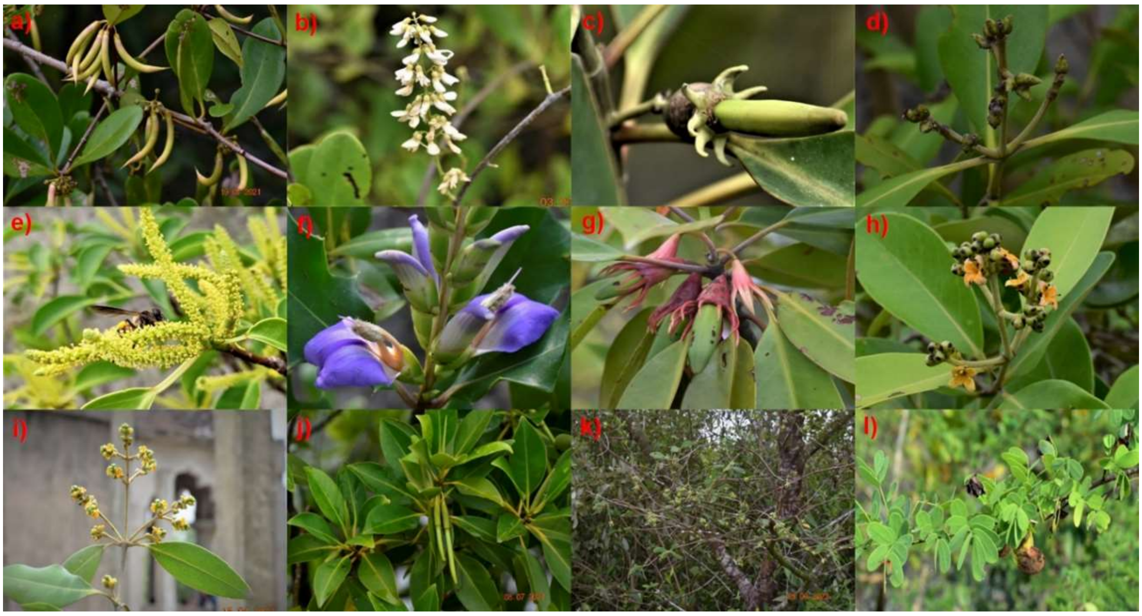

Supplementary Fig. 3.2 Images representing species-richness, some at early and some at mature phase of establishment from experimental sites of ongoing restoration activities along with the semi-restored site at Ramganga a) *Aegiceras corniculatum*, b) *Derris trifoliata*, c) *Bruguiera cylindrica, d) Avicennia marina, e) Excoecaria agallocha, f) Acanthus ilicifolius, g) Bruguiera gymnorrhiza, h) Avicennia officinalis, i) Avicennia alba, j) Bruguiera parviflora, k) Sonneratia apetala, l) Dalbergia spinosa*

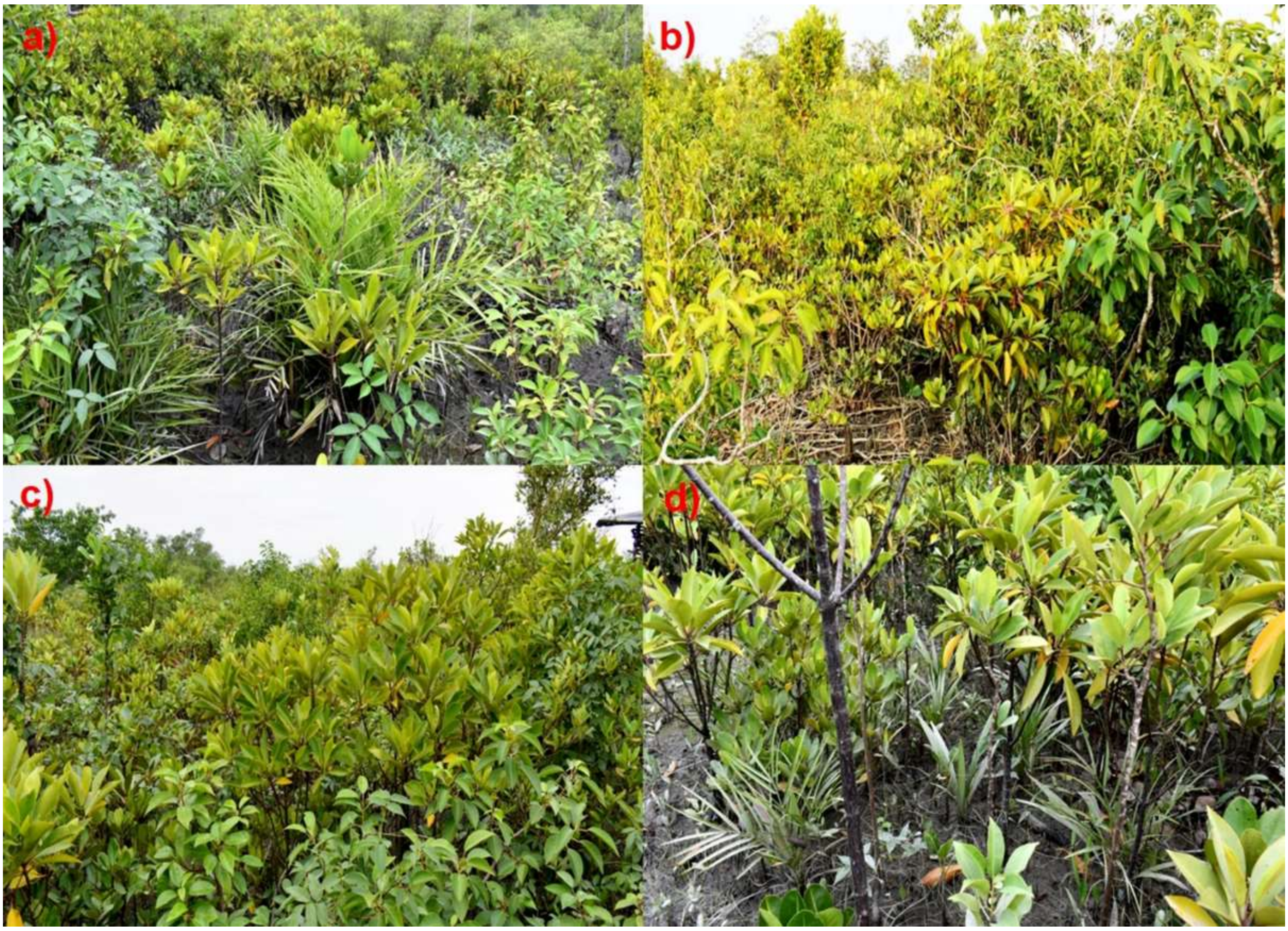

Supplementary Fig. 4 (a-d) Glimpses of multi-species assemblage of mangroves and mangrove associates at experimental semi-restored site at Ramganga at present

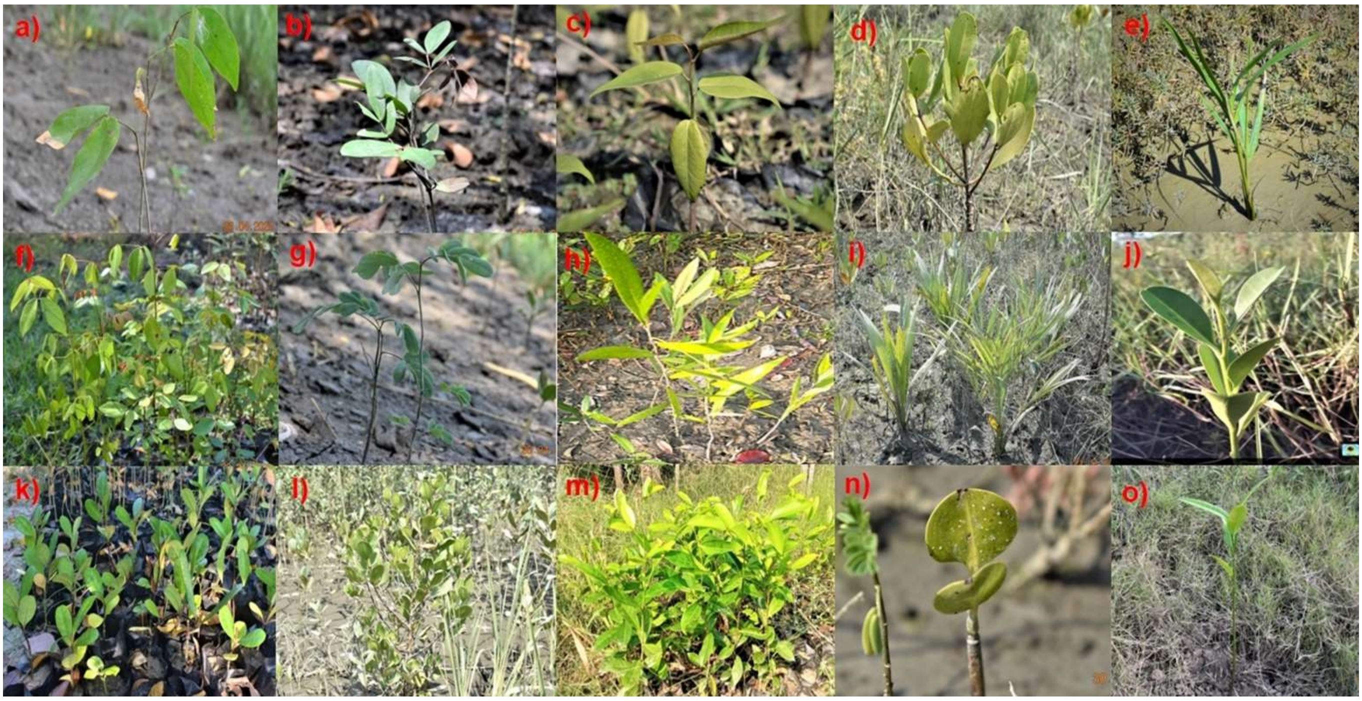

Supplementary Fig. 5 Different endangered, rare and threatened (RET) mangroves and mangrove associate species getting established at semi-restored site at Ramganga in 2023 a) *Intsia bijuga,* b) *Cynometra iripa,* c) *Brownlowia tersa,* d) *Ceriops decandra,* e) *Nypa fruticans,* f) *Caesalpinia crista,* g) *Dalbergia candenatensis,* h) *Heritiera fomes,* i) *Phoenix paludosa,* j) *Merope angulata,* k) *Acanthus volubilis,* l) *Lumnitzera racemosa,* m) *Acronychia pedunculata,* n) *Aegialitis rotundifolia,* o) *Cerbera manghas*

Supplementary Table 1 Total number of mangrove species, number of threatened species, survival%, plantation%, present compositional%/relative density at different mangrove sites

Degradation status	Total area of the sites (ha)	Total No. of species	No. of rare/near-threatened/endangered/locally rare species with decreasing population trend	Name of the species	Survival%	Plantation%	Present composition%<br><br>Relative density = (No. of individuals of the species /No. of individuals of all the species) x 100
Established semi-restored site at Ramganga	3	28	9	*Heritiera fomes*	3.75	3.59	0.24
				Xylocarpus spp	14.28	3.14	0.80
				Rhizophora mucronata	25.60	3.68	1.69
				Ceriops tagal	33.33	2.96	1.77
				Ceriops decandra	47.91	4.31	3.72
				Bruguiera cylindrica	61.66	5.39	5.98
				Bruguiera gymnorrhiza	36.58	18.42	12.13
				Bruguiera parviflora	88.88	4.04	6.47
				Aegialtis rotundifolia	76.66	2.69	3.72
				Aegiceras corniculatum	77.14	3.14	4.36
				Avicennia marina	82.69	4.67	6.95
				Avicennia officinalis	92.30	1.16	1.94
				Avicennia alba	87.17	3.50	5.50
				Sonneratia caseolaris	52.50	0.35	0.33
				Sonneratia apetala	54.00	0.44	0.43
				Phoenix paludosa	60.00	1.34	1.45
				Acanthus ilicifolius	73.33	1.34	1.77
				Excoecaria agallocha	80.00	0.89	1.29
				Derris trifoliata	66.66	0.80	0.97
				Dalbergia spinosa	85.71	0.62	0.97
				Nypa fruticans	2.30	0.11	0.004
				Intsia bijuga	33.33	0.01	0.008
				Brownlowia tersa	60.00	0.01	0.019

				Lumnitzera racemosa	76.92	0.01	0.016
				Paspalum vaginatum	55.00	5.39	5.33
				Porteresia coarctata	66.66	13.47	16.17
				Sporobolus virginicus	57.50	3.59	3.72
				Myriostachya wightiana	62.50	10.78	12.13
**Ongoing restoration sites**	**62**	**33**	**16**	*Acanthus ilicifolius*	46-94	0.01-3.587	0.001-2.431
				Aegialitis rotundifolia	75-96	0.17-4.68	0.003-4.9
				Heritiera fomes	3.75-98	0.03-3.59	0.001-3.59
				Aegiceras corniculatum	66.66-100	0.03-5.61	0.001-4.51
				Rhizophora mucronata	25.6-96	0.036-1.93	0.013-3.68
				Avicennia alba	71.42-100	0.035-8.7	0.001-3.5
				Avicennia marina	76.6-100	0.04-21.52	0.001-4.67
				Avicennia officinalis	75-100	0.11-53.81	0.006-30.6
				Bruguiera gymnorrhiza	36.58-98.62	0.18-64.58	0.05-69.36
				Bruguiera cylindrica	61.66-98.37	0.05-66.37	0.02-20.4
				Bruguiera parviflora	75-99.33	0.04-3.04	0.01-4.04
				Ceriops decandra	47.91-98.5	4.78-65.29	3.37-65.89
				Ceriops tagal	33.33-96.07	0.35-18.29	0.07-15.09
				Derris trifoliata	60-98	0.26-28.7	0.01-30.6
				Dalbergia spinosa	55-96	0.35-35.87	0.009-34.68
				Cynometra iripa	0-100	0.003-0.028	0-0.02
				Phoenix paludosa	25-98.8	0.16-1.86	0.004-2.04
				Intsia bijuga	25-100	0.003-0.086	0.0002-0.04
				Brownlowia tersa	40-100	0.01-0.71	0.002-0.244
				Excoecaria agallocha	75-100	0.01-12.55	0.0004-30.6
				Lumnitzera racemosa	50-100	0.03-0.179	0.004-0.183
				Nypa fruticans	40-93.75	0.03-0.574	0.001-0.612
				Caesalpinia crista	20-100	0.007-0.05	0.0001-0.04
				Merope angulata	100	0.003-0.007	0.004-0.008
				Xylocarpus sp	14.28-96.15	0.17-2.51	0.006-3.14
				Paspalum vaginatum	53.33-86	0.35-25.11	0.009-32.64
				Porteresia coarctata	65-88.88	0.35-71.75	0.01-32.64
				Sporobolus virginicus	57.5-90	0.57-17.93	0.16-18.36
				Myriostachya wightiana	62.5-86	0.35-53.81	0.04-51.08
				Kandelia candel	50-100	0.001-0.006	0.00001-0.007
				Dalbergia candenatensis	100	0.001-0.002	0.001-0.002
				Acronychia pedunculata	20-100	0.001-0.011	0.001-0.005

				Cerbera manghas	16-100	0.001-0.039	0.001-0.006
**Co-located Reference Pristine mangrove sites**		**34**	**13**	*Phoenix paludosa*	-	-	19.72-25.81
				Excoecaria agallocha	-	-	2.61-6.09
				Ceriops decandra	-	-	5.54-20.67
				Avicennia officinalis	-	-	1.57-1.59
				Avicennia marina	-	-	6.44-7.69
				Avicennia alba	-	-	2.07-6.47
				Xylocarpus mecongensis	-	-	0.152-2.60
				Xylocarpus granatum	-	-	0.85-2.60
				Aegiceras corniculatum	-	-	7.34-12.41
				Aegialitis rotundifolia	-	-	3.34-12.38
				Derris trifoliata	-	-	3.77-4.82
				Finlaysonia obovata	-	-	0.021-0.47
				Thespesia populnea	-	-	0.91-2.03
				Acronychia pedunculata	-	-	0.69-7.64
				Heritiera fomes	-	-	0.78-5.60
				Bruguiera cylindrica	-	-	0.63-0.89
				Caesalpinia crista	-	-	0.54-1.46
				Acanthus ilicifolius	-	-	2.19-22.30
				Acanthus volubilis	-	-	0.46
				Brownlowia tersa	-	-	0.02-0.43
				Intsia bijuga	-	-	0.15-0.22
				Ceriops tagal	-	-	0.08-1.33
				Sonneratia caseolaris	-	-	0.06-0.40
				Bruguiera gymnorrhiza	-	-	0.41-1.17
				Bruguiera parviflora	-	-	0.12-0.19
				Cynometra iripa	-	-	0.12-1.20
				Rhizophora mucronata	-	-	0.21-0.69
				Dalbergia candenatensis	-	-	0.061-0.063
				Lumnitzera racemosa	-	-	0.06-0.37
				Dalbergia spinosa	-	-	0.25-0.92
				Sarcolobus carinatus	-	-	4.42
				Hibiscus tiliaceus	-	-	0.92
				Porteresia coarctata	-	-	3.17
				Sonneratia apetala	-	-	0.12
				Acanthus ilicifolius	-	-	21.68
				Aegialitis rotundifolia	-	-	0.259

**Hinterland fringe species-poor mangrove site 1**		**12**	**1**	*Aegiceras corniculatum*	-	-	23.05
				Avicennia alba	-	-	20.51
				Avicennia marina	-	-	5.25
				Avicennia officinalis	-	-	1.88
				Bruguiera gymnorrhiza	-	-	0.519
				Dalbergia spinosa	-	-	2.597
				Derris trifoliata	-	-	6.623
				Excoecaria agallocha	-	-	0.389
				Porteresia coarctata	-	-	13.96
				Sonneratia apetala	-	-	3.24
**Hinterland fringe species-poor mangrove site 2**		**11**	**0**	*Acanthus ilicifolius*	-	-	41.406
				Aegiceras corniculatum	-	-	2.929
				Avicennia alba	-	-	8.593
				Avicennia marina	-	-	12.30
				Avicennia officinalis	-	-	3.320
				Bruguiera cylindrica	-	-	7.812
				Bruguiera gymnorrhiza	-	-	3.515
				Dalbergia spinosa	-	-	6.054
				Derris trifoliata	-	-	6.445
				Excoecaria agallocha	-	-	6.25
				Finlaysonia obovata	-	-	1.367
**Hinterland fringe species-poor mangrove site 3**		**12**	**2**	*Bruguiera cylindrica*	-	-	1.732
				Aegialitis rotundifolia	-	-	46.33
				Avicennia marina	-	-	17.95
				Avicennia officinalis	-	-	1.798
				Acanthus ilicifolius	-		25.347
				Aegiceras corniculatum	-	-	4.630
				Bruguiera gymnorrhiza	-	-	0.133
				Derris trifoliata	-	-	0.233
				Dalbergia spinosa	-	-	0.266
				Ceriops decandra	-	-	1.499
				Excoecaria agallocha	-	-	0.033
				Sonneratia caseolaris	-	-	0.033
**Degraded mangrove site 1**		**6**	**0**	*Avicennia alba*	-	-	1.699
				Avicennia marina	-	-	4.532
				Acanthus ilicifolius	-	-	48.72
				Excoecaria agallocha	-	-	1.133

				Porteresia coarctata	-	-	42.49
				Avicennia officinalis	-		1.416
**Degraded mangrove 2**		**6**	**0**	*Avicennia alba*	-	-	7.823
				Avicennia marina	-	-	7.482
				Acanthus ilicifolius	-	-	78.231
				Excoecaria agallocha	-	-	4.081
				Avicennia officinalis	-	-	1.700
				Derris trifoliata	-	-	0.680
**Monotypic plantation 1**		**2**	**0**	*Bruguiera gymnorrhiza*	-	-	80.76
				Ceriops tagal	-	-	19.23
**Monotypic plantation 2**		**2**	**0**	*Ceriops tagal*	-	-	93.75
				Bruguiera gymnorrhiza	-	-	6.25

Supplementary Table 2

Species present at degraded mangrove sites and pristine mangroves of Bhagabatpur, Daspur, Dhonchi and Ramganga, the restored site at 2022 in the Western part of Indian Sundarbans

Serial no.	Name of the species present at Degraded mangroves	Name of the species present at Bhagabatpur pristine forest	Name of the species present at Daspur pristine forest	Name of the species present at Dhonchi pristine forest	Name of the species present at semi-restored site at Ramganga 2021-22
1	*Acanthus ilicifolius*	*Acanthus ilicifolius*	*Acanthus ilicifolius*	*Acanthus ilicifolious*	*Acanthus ilicifolius*
2	*Avicennia alba*	*Acanthus volubilis*	*Acanthus volubilis*	*Acronychia pedunculata*	*Aegialitis rotundifolia*
3	*Avicennia marina*	*Acronychia pedunculata*	*Avicennia alba*	*Aegialitis rotundifolia*	*Aegiceras corniculatum*
4	*Avicennia officinalis*	*Aegialitis rotundifolia*	*Avicennia marina*	*Aegiceras corniculatum*	*Avicennia alba*
5	*Porteresia coarctata*	*Aegiceras corniculatum*	*Avicennia officinalis*	*Avicennia alba*	*Avicennia marina*
6	*Excoecaria agallocha*	*Avicennia alba*	*Aegiceras corniculatum*	*Avicennia marina*	*Avicennia officinalis*
7	*Derris trifoliata*	*Avicennia marina*	*Aegialitis rotundifolia*	*Avicennia officinalis*	*Brownlowia tersa*
8	*Myriostachya wightiana*	*Avicennia officinalis*	*Acronychia pedunculata*	*Bruguiera cylindrica*	*Bruguiera cylindrica*
9		*Brownlowia tersa*	*Bruguiera gymnorrhiza*	*Bruguiera gymnorrhiza*	*Bruguiera gymnorrhiza*
10		*Bruguiera cylindrica*	*Bruguiera cylindrica*	*Bruguiera parviflora*	*Bruguiera parviflora*
11		*Bruguiera gymnorrhiza*	*Bruguiera parviflora*	*Caesalpinia crista*	*Ceriops decandra*
12		*Bruguiera parviflora*	*Brownlowia tersa*	*Ceriops decandra*	*Ceriops tagal*
13		*Caesalpinia crista*	*Ceriops decandra*	*Ceriops tagal*	*Dalbergia spinosa*
14		*Ceriops decandra*	*Ceriops decandra*	*Cynometra iripa*	*Derris trifoliata*
15		*Ceriops tagal*	*Caesalpinia crista*	*Dalbergia candenatensis*	*Excoecaria agallocha*
16		*Cynometra iripa*	*Dalbergia spinosa*	*Dalbergia spinosa*	*Finlaysonia obovata*
17		*Dalbergia candenatensis*	*Dalbergia candenatensis*	*Derris scandens*	*Heritiera fomes*
18		*Dalbergia spinosa*	*Derris trifoliata*	*Derris trifoliata*	*Intsia bijuga*
19		*Derris trifoliata*	*Excoecaria agallocha*	*Excoecaria agallocha*	*Myriostachya wightiana*
20		*Excoecaria agallocha*	*Finlaysonia obovata*	*Finlaysonia obovata*	*Nypa fruticans*
21		*Finlaysonia obovata*	*Heritiera fomes*	*Heritiera fomes*	*Paspalum vaginatum*
22		*Heritiera fomes*	*Intsia bijuga*	*Hibiscus tiliaceus*	*Phoenix paludosa*
23		*Intsia bijuga*	*Lumnitzera racemosa*	*Intsia bijuga*	*Porteresia coarctata*

24		*Phoenix paludosa*	*Merope angulata*	*Lumnitzera racemosa*	*Rhizophora apiculata*
25		*Rhizophora apiculata*	*Phoenix paludosa*	*Phoenix paludosa*	*Sonneratia apetala*
26		*Sonneratia apetala*	*Rhizophora mucronata*	*Porteresia coarctata*	*Sonneratia caseolaris*
27		*Sonneratia caseolaris*	*Sonneratia apetala*	*Rhizophora mucronata*	*Sporobolus virginicus*
28		*Thespesia populnea*	*Sonneratia caseolaris*	*Sonneratia apetala*	*Xylocarpus mekongensis*
29		*Xylocarpus granatum*	*Sarcolobus carinatus*	*Sonneratia caseolaris*	
30		*Xylocarpus mecongensis*	*Xylocarpus mekongensis*	*Thespesia populnea*	
31			*Porteresia coarctata*	*Xylocarpus spp*	

Supplementary Table 3

Survival rate of transplanted mangrove seedlings covering 31 ongoing mangrove restoration sites

Sl no.	Name of species	No. of saplings transplanted to the site of restoration	No. of saplings survived at present	% of survival at present	Height of the seedlings when transplanted (range in cm)	Height of the seedlings at present (range in cm)
1	*Acanthus ilicifolius*	25350	22446	88.54	8.3-18.2	8.9-88.9
2	*Acronychia pedunculata*	17	7	41.17	5.6-8.1	5.8-17
3	*Aegialitis rotundifolia (Near threatened)*	7270	6000	82.53	8.4-14	6.9-67
4	*Aegiceras corniculatum*	17240	15500	89.9	13-26	10.9-50.2
5	*Avicennia alba*	24500	21920	89.46	18-25	56-338
6	*Avicennia marina*	33650	30710	91.26	11.2-45	15-128
7	*Avicennia officinalis*	41400	37940	91.64	11-54.4	12.3-148
8	*Brownlowia tersa (Near threatened)*	860	580	67.44	10.2-23	13.4-64.5
9	*Bruguiera cylindrica*	57790	52965	91.65	10.5-25	14.4-99.2
10	*Bruguiera gymnorrhiza*	71135	66730	93.8	25-41	21.2-110
11	*Bruguiera parviflora*	6945	6185	89.05	16-22	14.1-72.2
12	*Caesalpinia crista*	92	40	43.47	10-32.2	15.4-72.6
13	*Cerbera manghas*	76	47	61.84	11-23.2	15.4-62.2
14	*Ceriops decandra (Near threatened)*	263170	240600	91.42	10.2-18	9.1-91.3
15	*Ceriops tagal*	32580	29535	90.65	21-37	18.6-80
16	*Cynometra iripa*	52	31	59.61	12-21.2	18.6-41.1
17	*Dalbergia spinosa*	21510	18895	87.84	9-27.5	13.9-141.6
18	*Derris trifoliata*	22245	20775	93.39	15.6-25.4	17-135
19	*Excoecaria agallocha*	20285	18705	92.21	15.2-33	31-86.8
20	*Finlaysonia obovata*	115	78	67.82	12.4-55	14.5-199
21	*Heritiera fomes (Endangered)*	4878	4032	82.65	20-45	26.1-88

22	*Intsia bijuga (Near threatened)*	96	49	51.04	10.3-32	14.9-65.6
23	*Kandelia candel*	35	28	80	10-32.3	14.3-75.8
24	*Lumnitzera racemosa*	313	261	83.38	8.3-23	12.6-72.8
25	*Merope angulata*	3	3	100	7.7-10.5	14.2-20.4
26	*Nypa fruticans*	905	700	77.34	11-27.5	15.2-98.5
27	*Phoenix paludosa (Near threatened)*	2810	2508	89.25	14-23.8	15.1-59.1
28	*Rhizophora mucronata*	10378	8555	82.43	40-70	60.4-153.4
29	*Sonneratia apetala*	7963	7038	88.38	12.6-75	16.2-113
30	*Sonneratia caseolaris*	129	95	73.64	21-45	65-190
31	*Xylocarpus mekongensis*	6633	5610	84.57	15-76	25.4-119
32	*Porteresia coarctata*	63300 (average 7 tillers)	52000 (average 7 tillers)	82.14	12.5-45.7	17.8-85.3
33	*Myriostachya wightiana*	43500 (average 6 tillers)	31000 (average 6 tillers)	71.26	12.3-33.6	27.1-92.8
34	*Paspalum vaginatum*	36123 (average 6 tillers)	25600 (average 6 tillers)	70.86	10.1-17	16.5-78.3
35	*Sporobolus virginicus*	24061 (average 7 tillers)	17000 (average 7 tillers)	70.65	6.3-16.3	12.2-81.2
	**TOTAL**	**847409**	**725168**	**85.57**	**5.6-76**	**5.8-338**

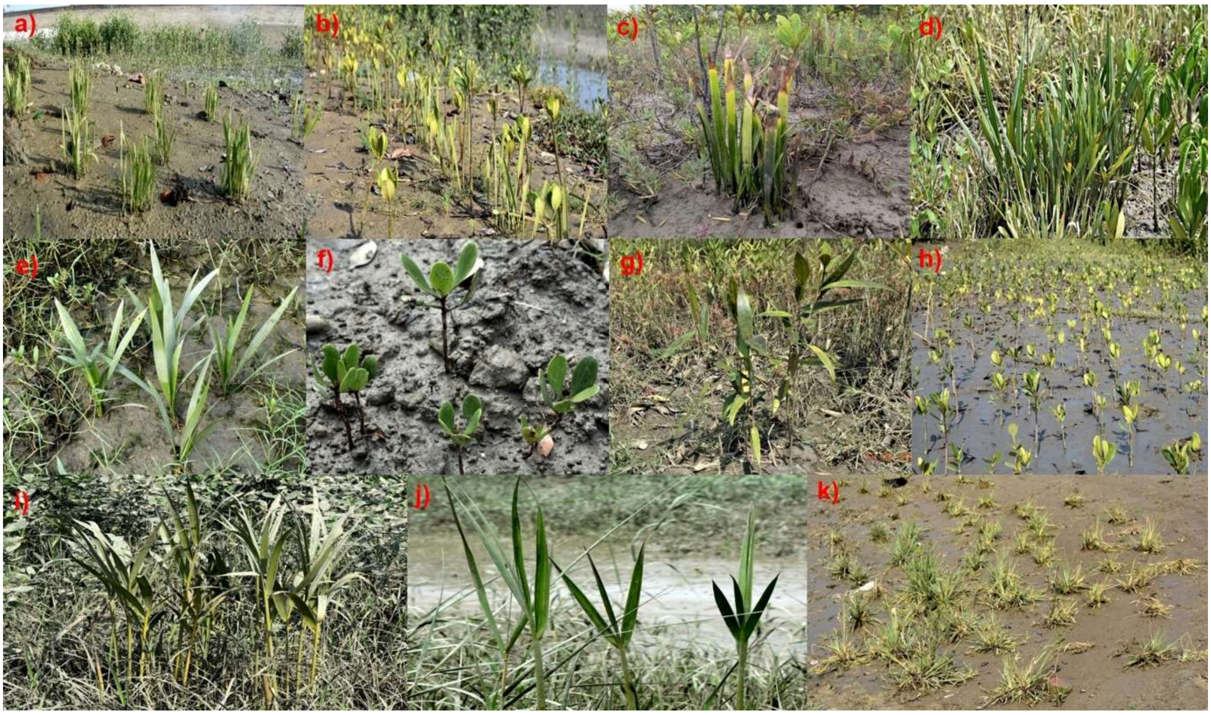

Supplementary Fig. 6 Images representing the facilitative approach in mangrove restoration. a)-e) Very closely planted *Phoenix paludosa* (≤5≤40 cm gap), f) *Lumnitzera racemosa* (≤40 cm gap), g) *Heritiera fomes* (≤40 cm gap) h) *Ceriops decandra* (≤40 cm gap), i)-j) *Nypa fruticans* (≤40 cm gap), k) Closely planted halophytic grasses (≤40 cm gap)

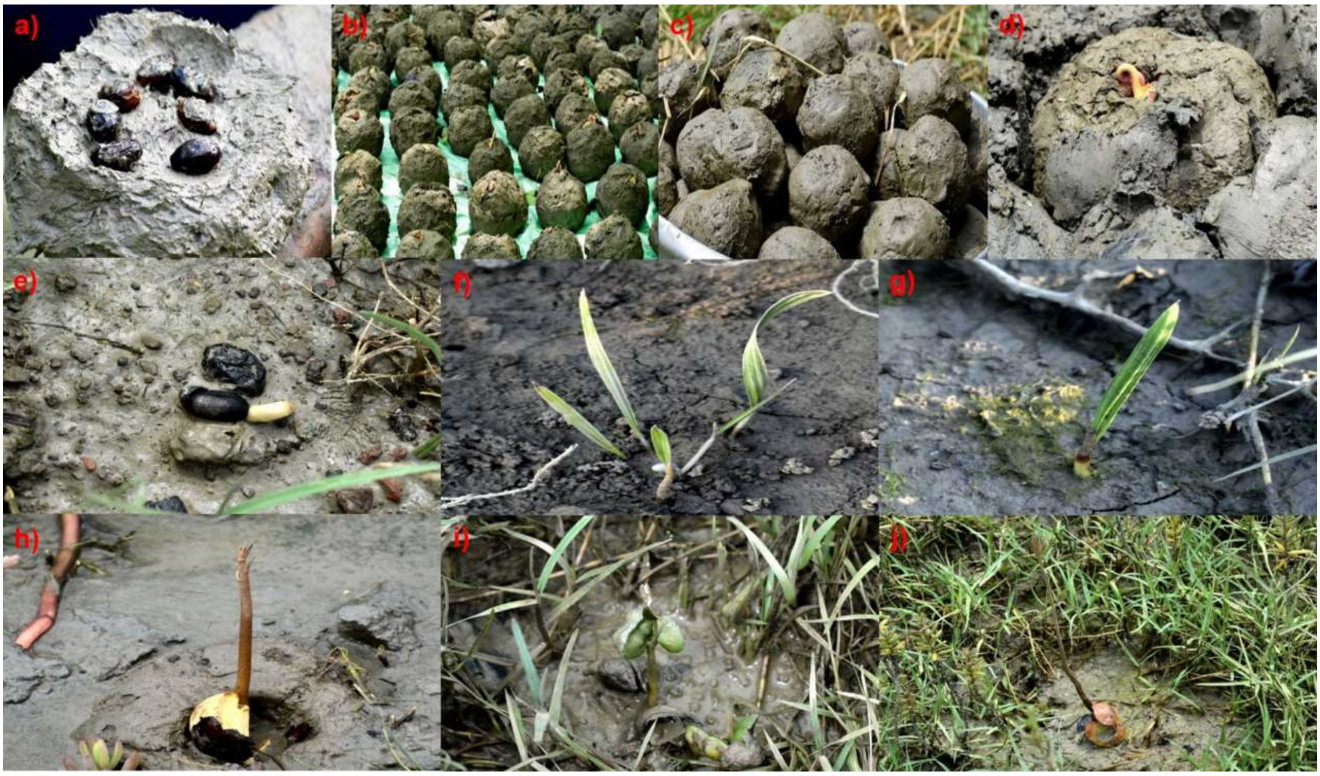

Supplementary Fig. 7 Seed ball use technology (a-c) Different stages of seed ball preparation, (d) Seed ball dispersal at restoration site, (e) Emergence of the seedlings from seed ball of *Phoenix paludosa,* (f-g)

Established seedlings from seed ball of *Phoenix paludosa,* (h) Seedling emergence from *Heritiera fomes,* (i) Seedling emergence from *Brownlowia tersa* seed ball, (j) Seedling emergence from *Heritiera fomes*

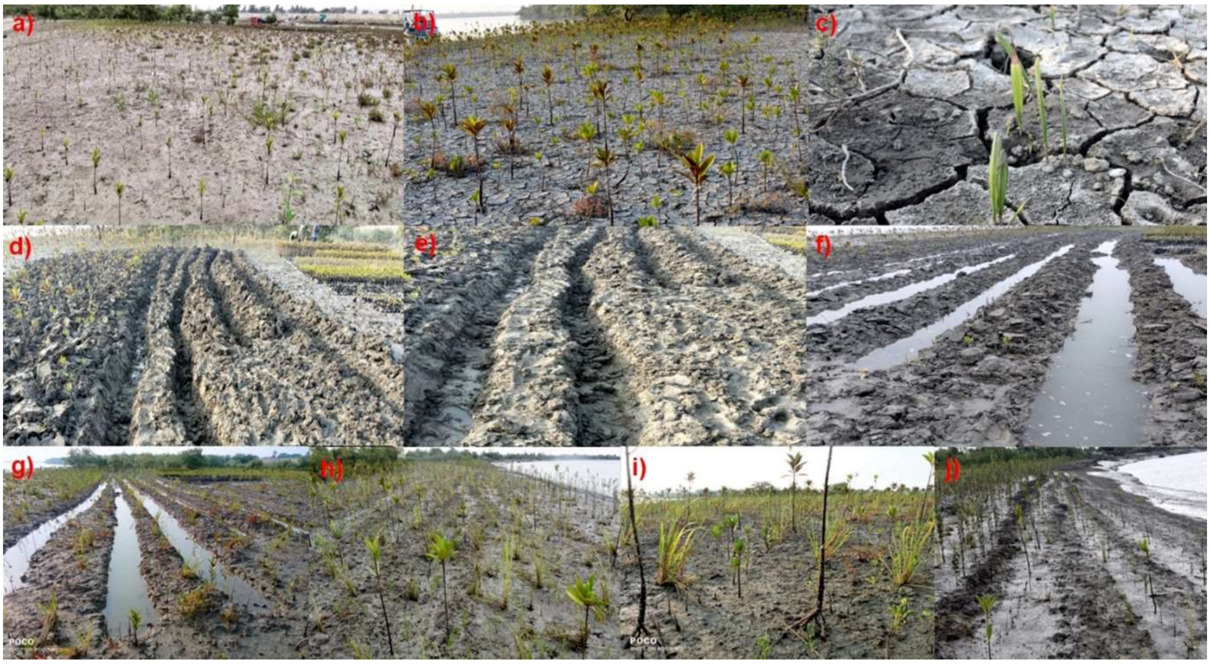

Supplementary Fig. 8 Channel grooving technique to improve hydrology of site **(**a-c) Showing the cracked high-tidal mudflat due to lack of tidal water invasion during high tide, (d-e) Drainages have been dug up, so that tidal water can invade and are accumulated for some time, (f) High tides invade the drainages, deposition of silt occurs (g-j) Transplantation and rejuvenation of mangrove seedlings after water made available to them.

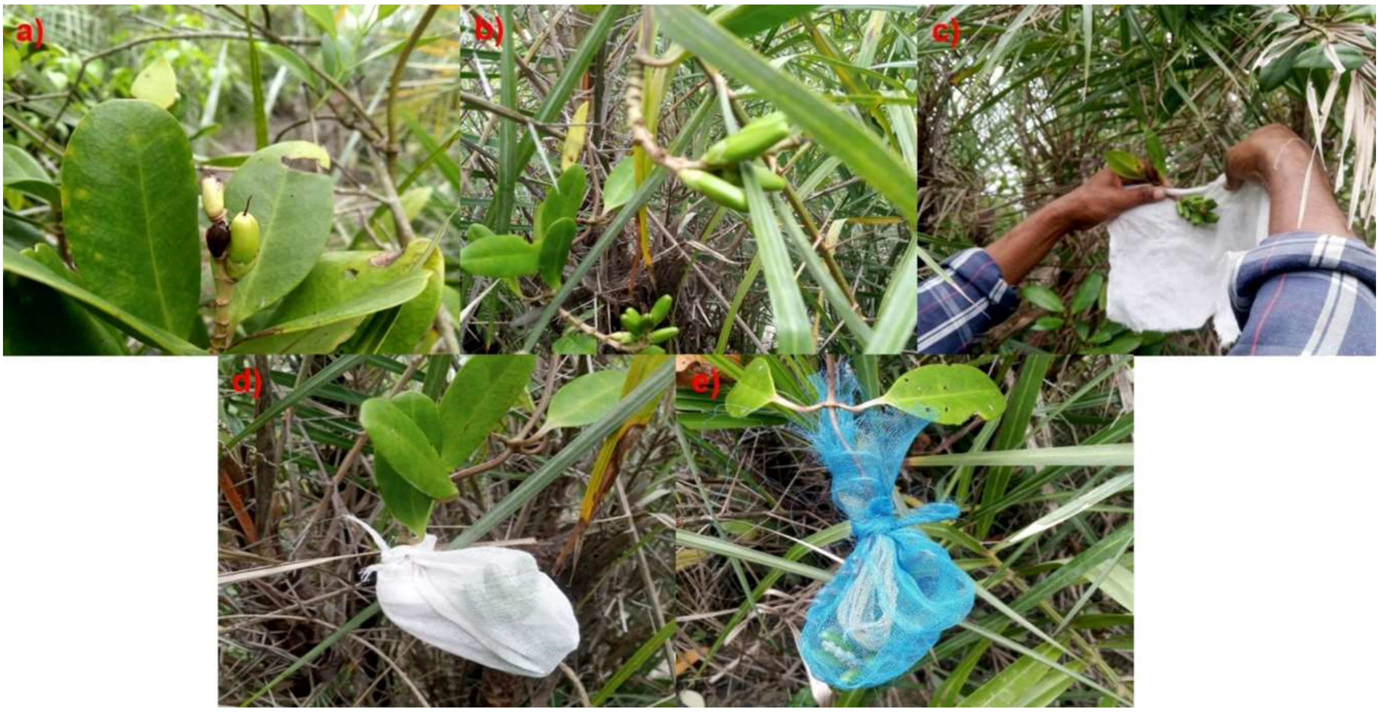

Supplementary Fig. 9 Special bagging technique used for collection of locally rare propagules of *Acanthus volubilis* from their natural habitat. (a-b) The immature fruits of *Acanthus volubilis,* (c-e) the seeds were wrapped with the cotton clothes/fishing net for collection at later stages until their maturity

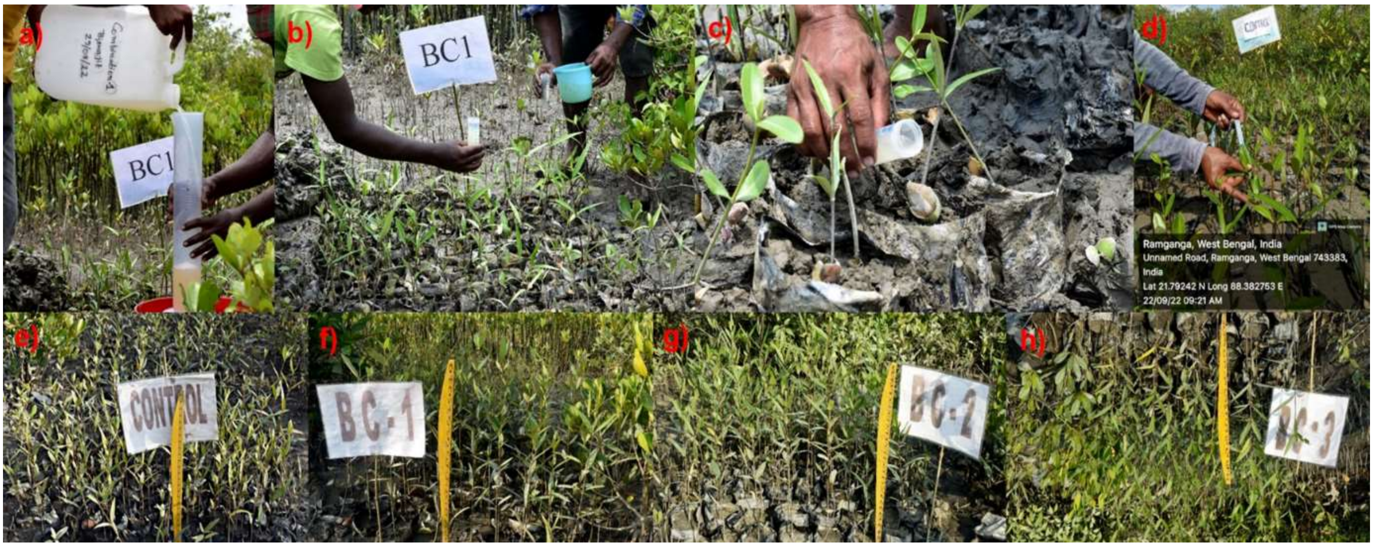

Supplementary Fig. 10 Images representing on-field application of plant growth promoting bacterial consortia. a)–c) Addition of bacterial consortia in appropriate volume and dilution, d) Measuring leaf width of mangrove seedlings, e)-h) Measuring of shoot length of mangrove seedlings from experimental plots with three different bacterial consortia

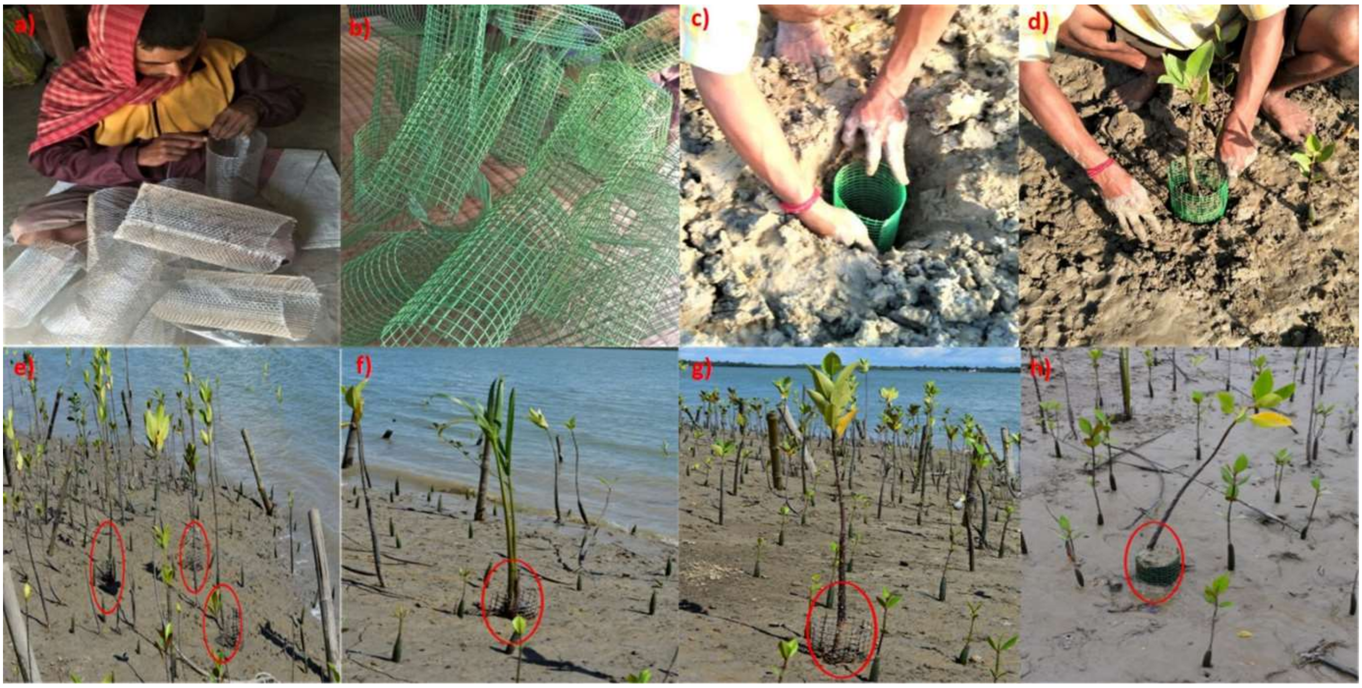

Supplementary Fig. 11 A special restoration technology for vulnerable eroded cut edges of the restoration site at Ramganga (a-b) Images showing the preparation of iron cages, c) The cages inserted into the mud, d) Mangrove seedlings transplanted within the cage, (e-h) The transplanted seedlings come up with their head high, the cage anchors the soil and gives the seedlings a mechanical strength during their very young stage and protect the roots till the establishment of the seedlings and do not let it washed away by tidal current.

Supplementary Table 4: Details of PGPR bacterial consortia used

BC1 consortium

Name of the bacterial stain	Accession no.	Source of mangrove species
Aeromonas dhakensis strain HPR7	MT422007	*Heritiera fomes* pneumatophore
Mangrovibacter plantisponcer strain BCRP5	MT422011	*Bruguiera cylindrica* root
Pseudomonas stutzeri strain BCY5	MT422041	*Bruguiera cylindrica* root
Pseudomonas stutzeri strain BCY7	MT422042	*Bruguiera cylindrica* root
Bacillus subtilis strain AOR5	MT421976	*Avicennia officinalis* root
Serratia marcescens strain AOR4	MT422009	*Avicennia officinalis* root

BC2 consortium

Name of the bacterial stain	Accession no.	Source of mangrove species
Aeromonas allosaccharophila strain DAL2	MT422027	*Dalbergia spinosa* root
Pseudomonas sp. strain DER1	MT422045	*Derris trifoliata* root
Pseudomonas putida strain DER3	MT422020	*Derris trifoliata* root
Pseudomonas fulva strain DER9	MT422023	*Derris trifoliata* root
Aeromonas veronii strain POT3	MT422035	*Portresia coarctata* root
Aeromonas veronii strain POT7	MT422025	*Portresia coarctata* root
Serratia marcescens strain HPR4	MT422006	*Heritiera fomes* pneumatophore

BC3 consortium

Name of the bacterial stain	Accession no.	Source of mangrove species
Bacillus subtilis strain AMR4	MT421979	*Avicennia marina* root
Aeromonas hydrophilia strain HER3	MT422047	*Heritiera fomes* root
Bacillus altitudinis strain XYL1	MT422037	*Xylocarpus* sp. root
Pseudocitrobacter faecalis strain HRR5	MT422004	*Heritiera fomes* root
Serratia marcescens strain HPR4	MT422006	*Heritiera fomes* pneumatophore
Enterobacter kobei strain HRR1	MT421989	*Heritiera fomes* root

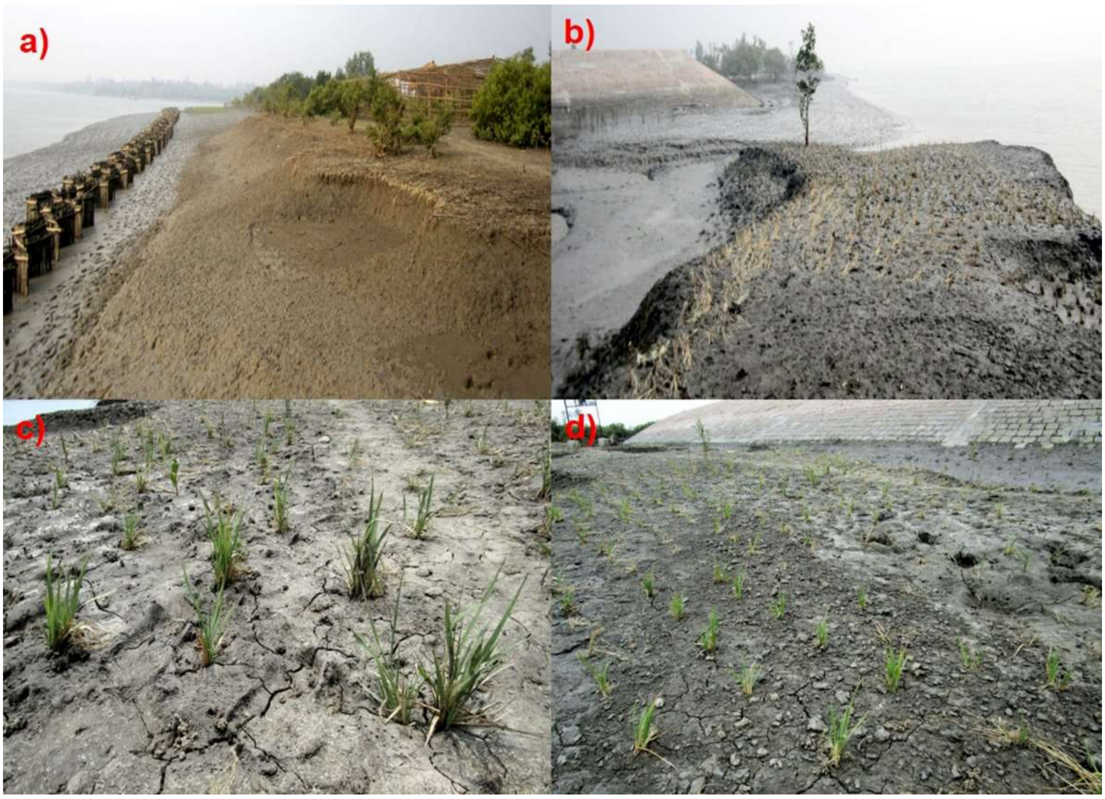

Supplementary Fig. 12 (a-b) the experimental degraded mangrove site at Ramganga at a glance in 2014 harbouring only few scanty stunted discontinuous stands of 8 species of mangroves (c-d) Beginning of establishment of M*yriostachya wightiana* at the eroded river bank in 2014

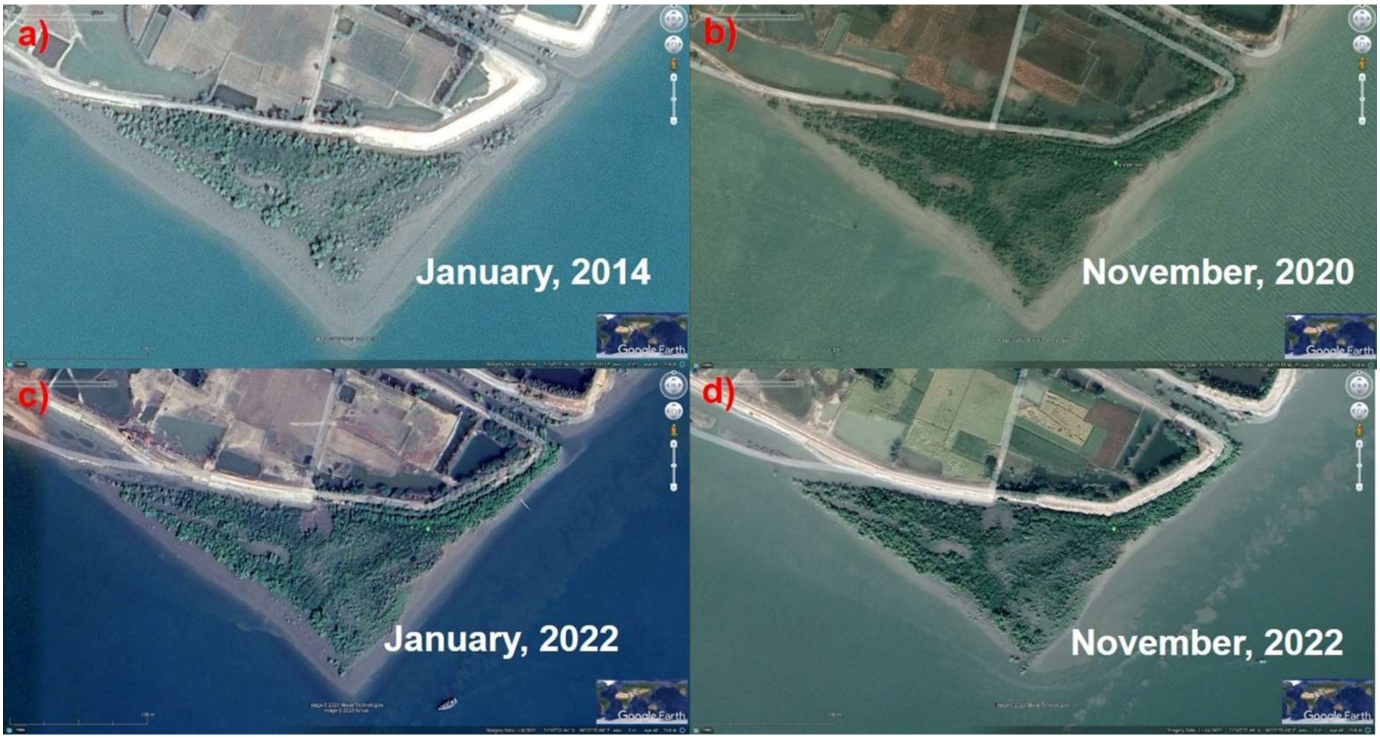

Supplementary Fig. 13 Google Earth images of Ramganga semi-restored site. a) Before restoration January 2014, b) After 6 years of restoration November 2020, c) After 7 years of restoration January 2022, d) After 8 years of restoration November 2022

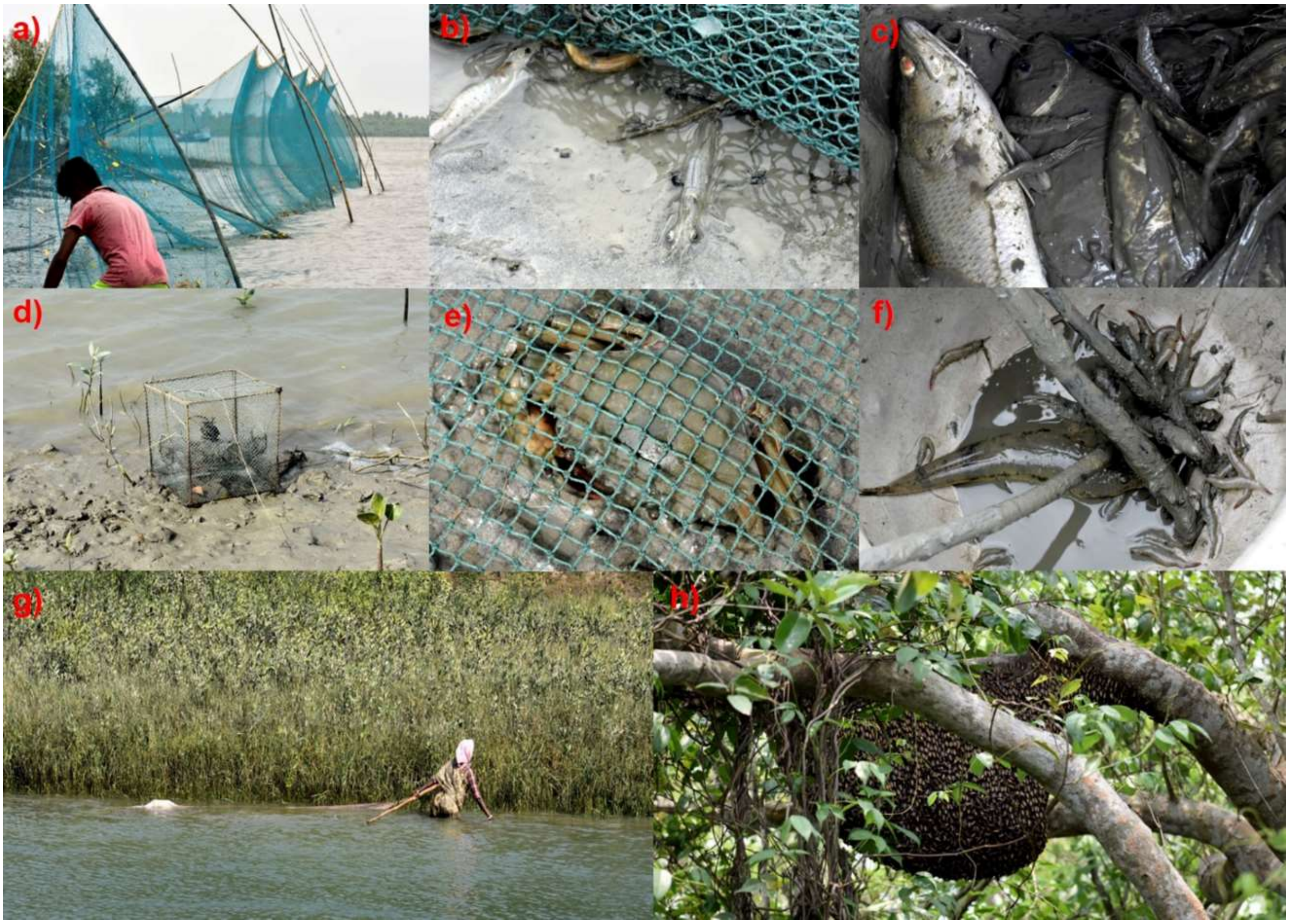

Supplementary Fig. 14 Ecosystem services at the restoration site a) Stake net fishing b-c & e-f) Cluster of fish catch, shrimp and crab catching by stake net fishing d) Crab hunting net at placed at our restoration site g) Catching of prawn seed at restoration site h) Bee-hive formed at our semi-restored Ramganga site

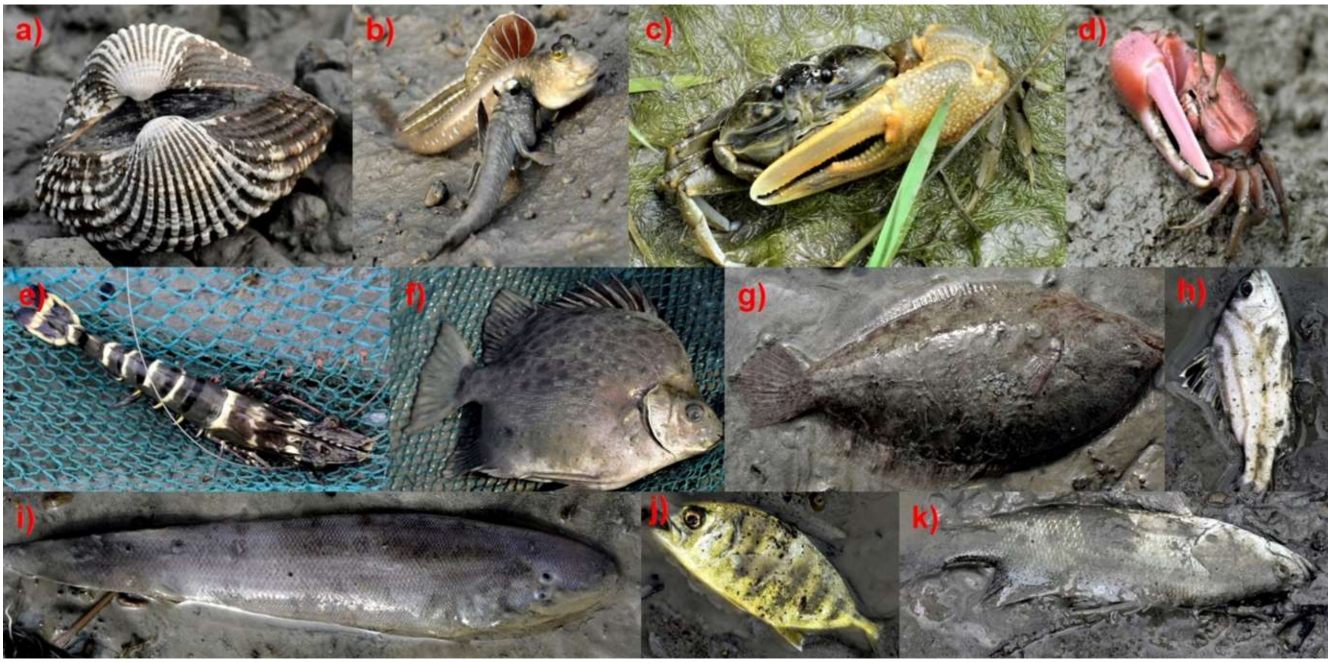

Supplementary Fig. 15 Images representing the epifaunal and fish diversity of established experimental semi-restored site, Ramganga a) *Anadara* spp. , b) *Boleophthalmus boddarti,* c) *Austruca* sp., d) *Tubuca rosea (*red fiddler crab*),* e) *Penaeus monodon,* f) *Scatophagus* sp.*,* g) *Pseudorhombus* sp.*,* h) *Terapon jarbua,* i) *Cynoglosus* sp.*,* j) *Caranx* sp.*,* k) *Lates calcarifer*

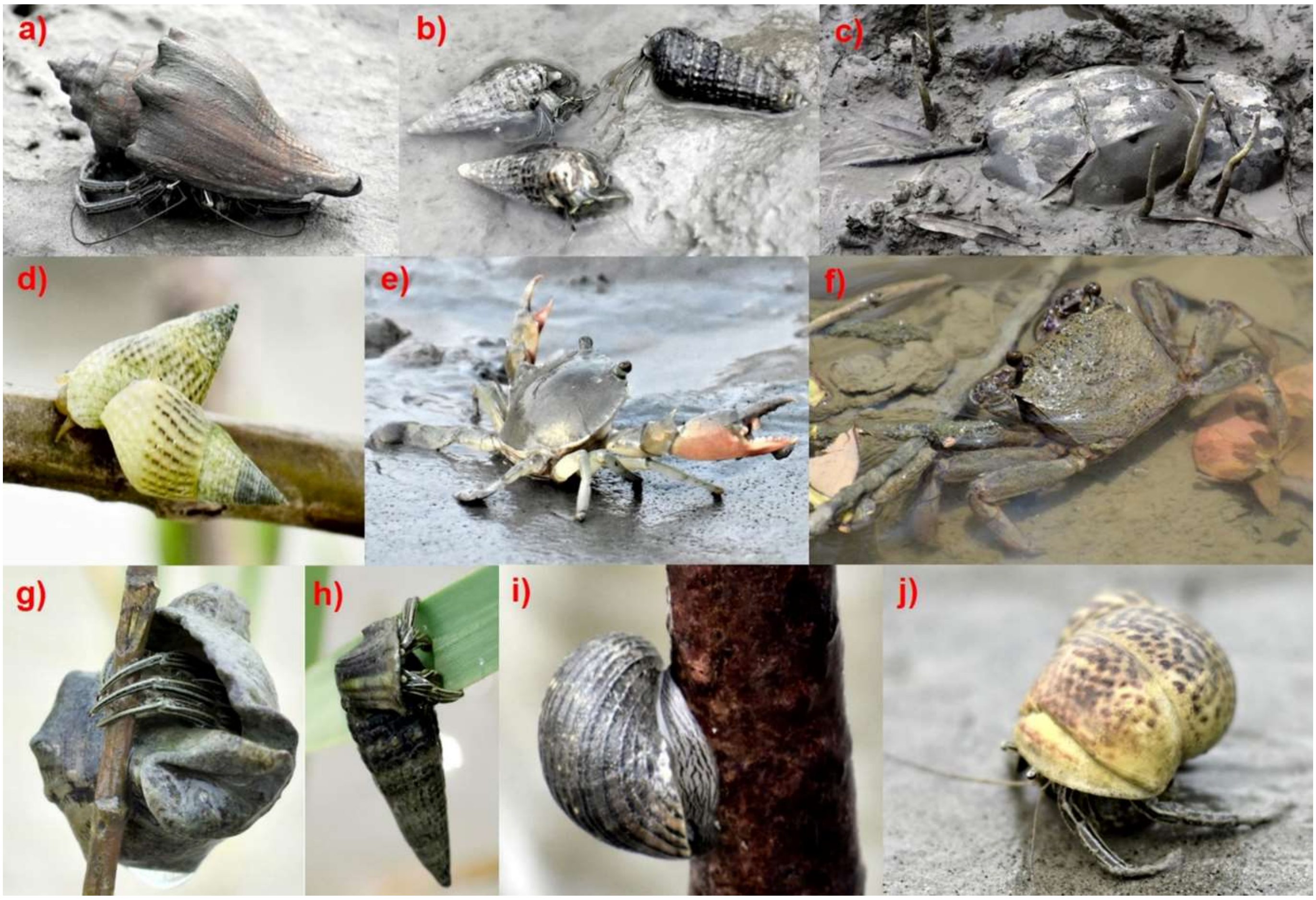

Supplementary Fig. 16 Images representing the epifaunal diversity of established experimental semi-restored site, Ramganga a) *Clibanarius vittatus* (Striped Hermit crab under *Pugilina cochlidium* shell), b) *Cerithidia cingulata* shell protecting thin striped hermit crab (Horn shell), c) Mangrove horseshoe crab *Carcinoscorpius rotundicauda*, d) *Littoraria melanostoma,* e) *Scylla serrata* (mud crab), f) *Episesarma mederi,* g) *Clibanarius* sp., h) *Clibanarius* sp. (within the shell of *Cerithedia cingulata),* i) *Nerita articulata,* j) *Clibanarius* sp. (within the shell of *Littoraria)*

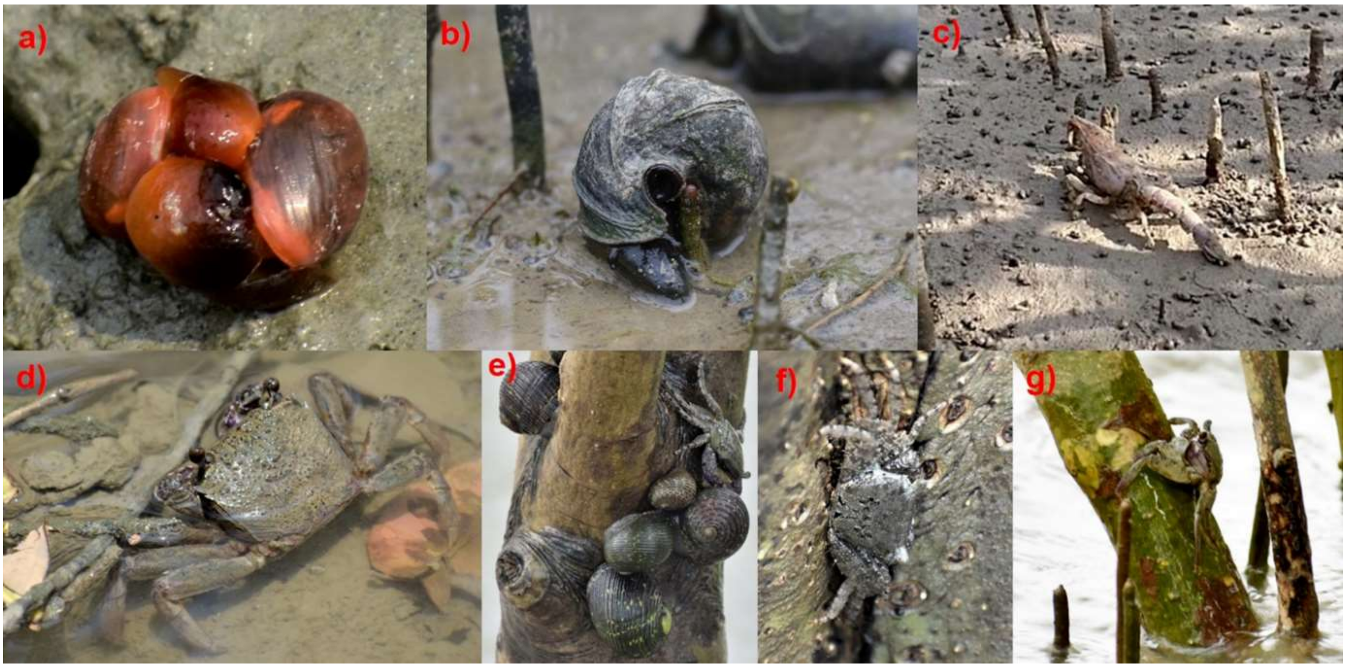

Supplementary Fig. 17 Images representing the epifaunal diversity of established experimental semi-restored site, Ramganga a) *Neripteron violaceum,* b) *Teliscopium* sp., c) *Thalassina anomala,* d) *Episesarma mederi*, e) A association of *Nerita articulata and Sesarma* sp., f) *Episesarma mederi,* g) *Episesarma mederi* on a tree trunk

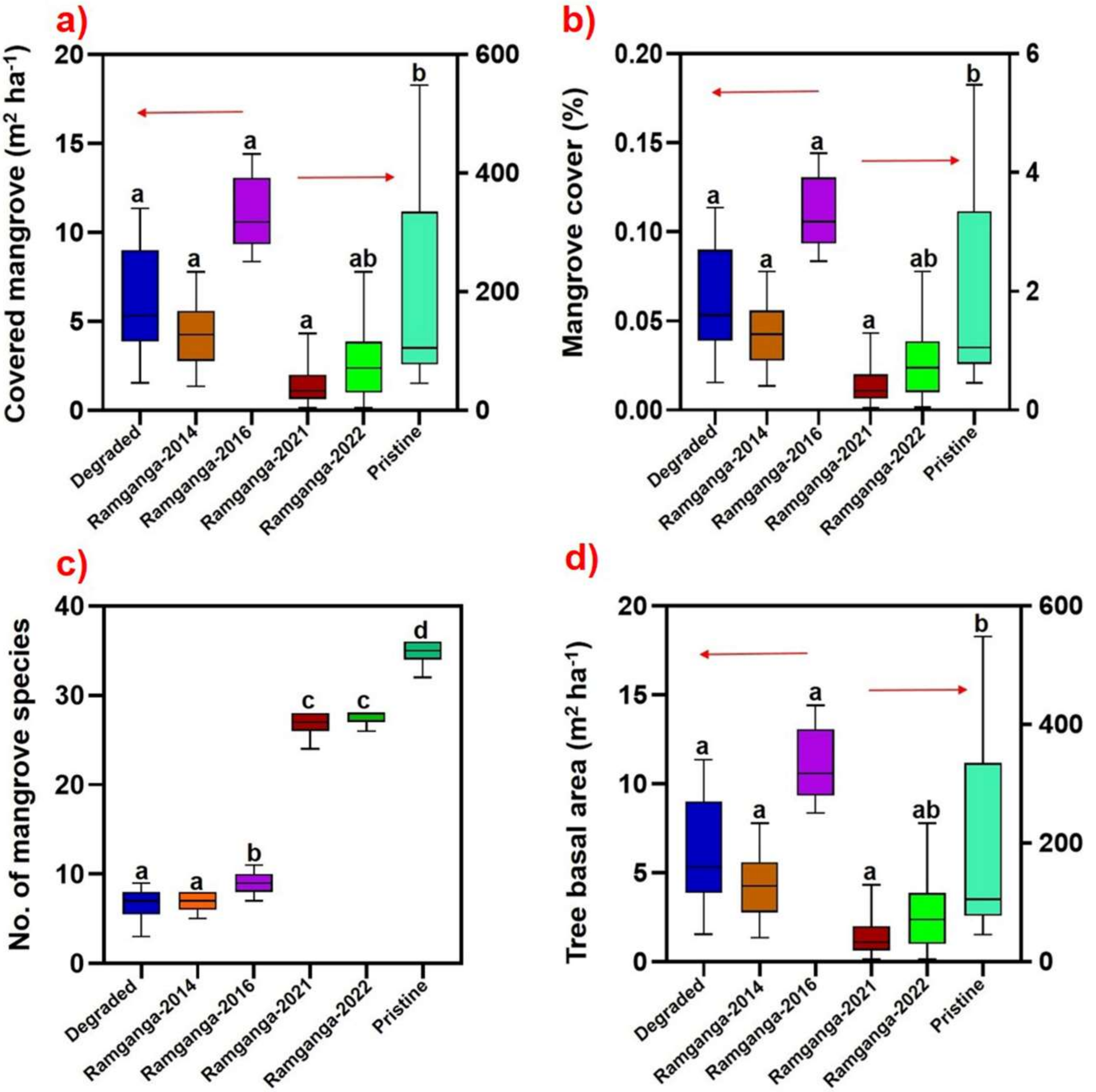

Supplementary Fig. 18 Box-whisker plot displaying the structural features of mangrove vegetation among the experimental sites: Degraded, Ramganga 2014, Ramganga 2016, Ramganga 2021, Ramganga 2022 and Pristine mangrove forest a) Covered mangrove area, b) Mangrove cover% c) No. of mangrove species, d) Tree basal area. Whiskers represent the range of data from highest to lowest with a median value. Here n=25. Values designated with different letters are significantly different at the 5% level.

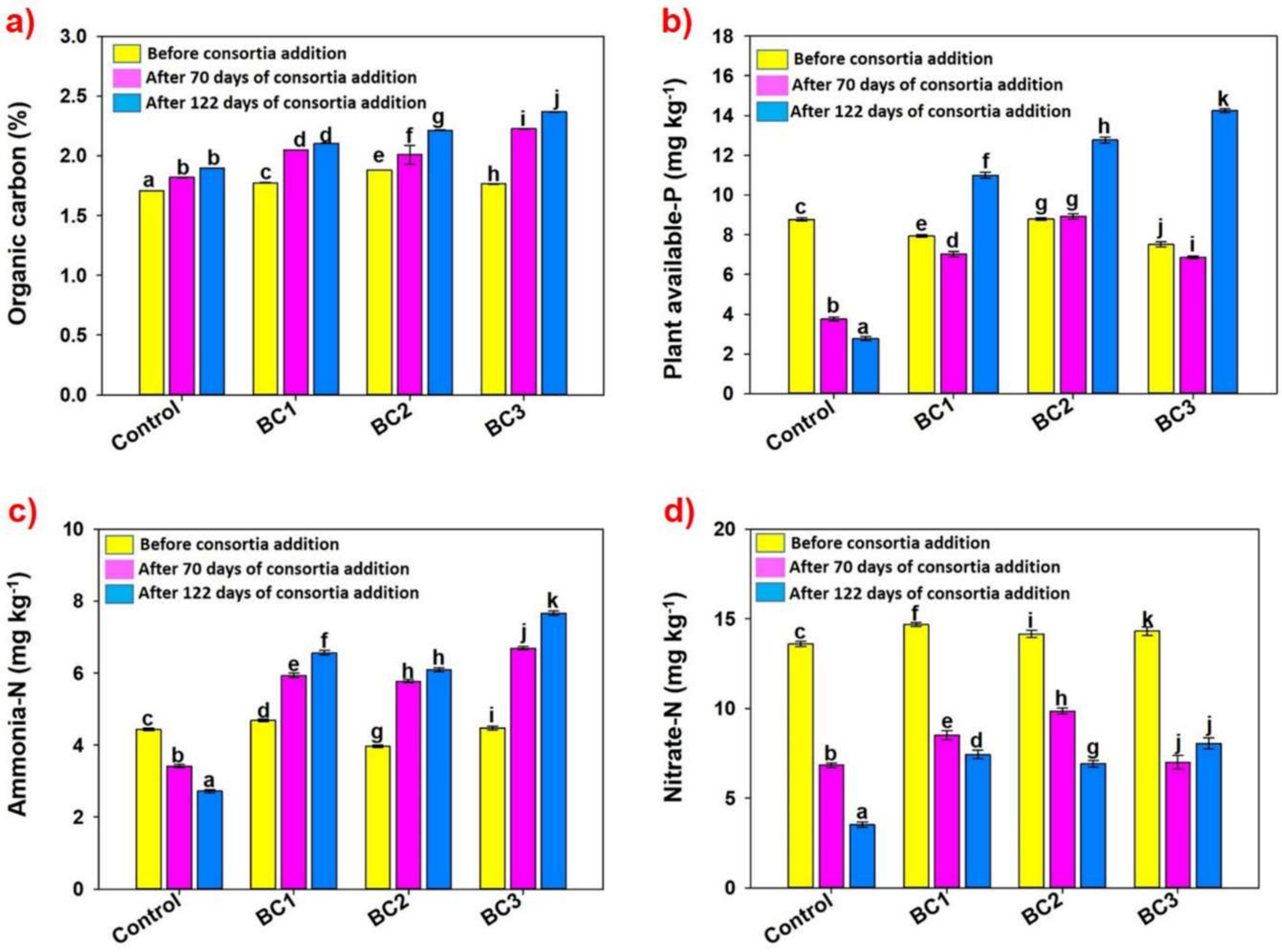

Supplementary Fig. 19 Bar diagram displaying the nutrient status (Y axis) of *Avicennia* spp. rhizospheric sediments before consortia addition (Yellow), after 70 days of consortia addition (Violet), after 122 days of consortia addition (Blue), grouped by treatments i.e. Control, BC1, BC2 and BC3 (X axis): a) Organic carbon, b) Plant available-P, c) Ammonia-N, d) Nitrate-N. Here n=10. Values designated with different letters are significantly different at the 5% level.

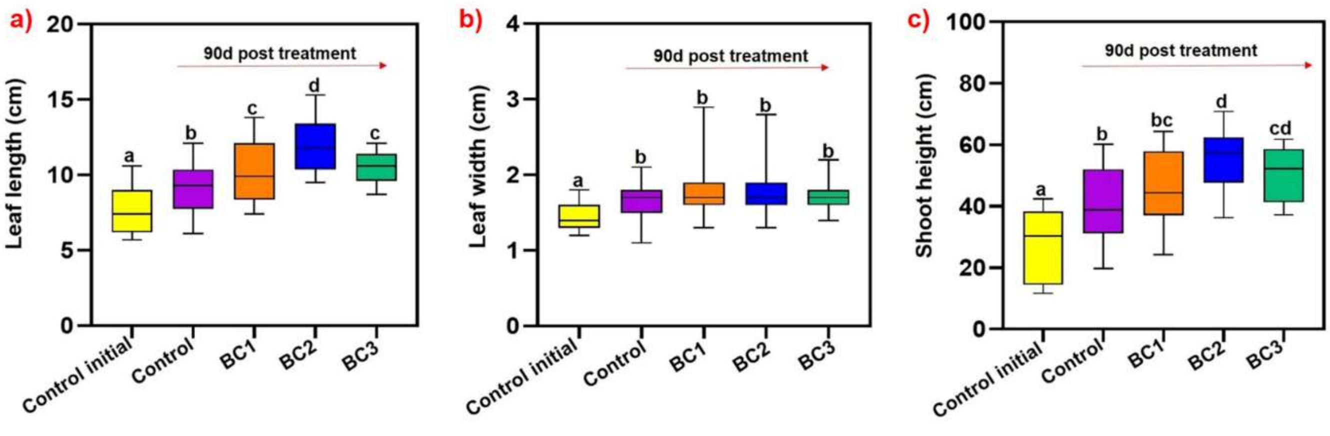

Supplementary Fig. 20 Box-whisker plots representing 3 morphological parameters (Y-axis) of *Avicennia* spp. Control initial and after 90 days of bacterial consortia (Control, BC1, BC2, BC3) addition (X-axis) in the

mangrove rhizosphere: a) Leaf length, b) Leaf width, c) Shoot height. Whiskers represent the range of data from highest to lowest with median value. Here n=25**.** Values designated with different letters are significantly different at the 5% level.

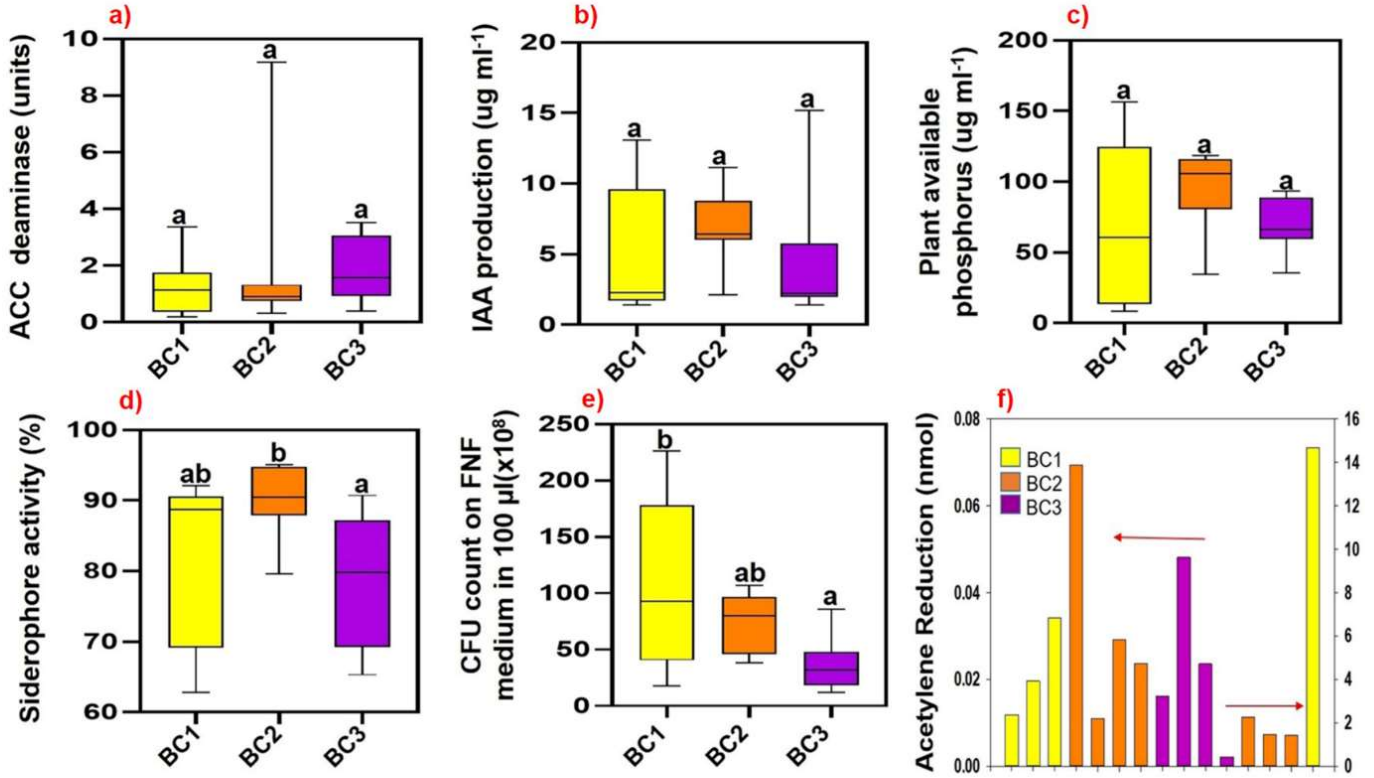

Supplementary Fig. 21 Box-whisker and Bar diagram plot displaying the PGPR activity between the three combinations BC1, BC2 and BC3 a) ACC deaminase, b) IAA production, c) Plant available-P, d) Siderophore activity, e) CFU count on FNF medium, f) Acetylene Reduction Assay (ARA). Whiskers represent the range of data from highest to lowest with median value. Here n=12. Values designated with different letters are significantly different at the 5% level.

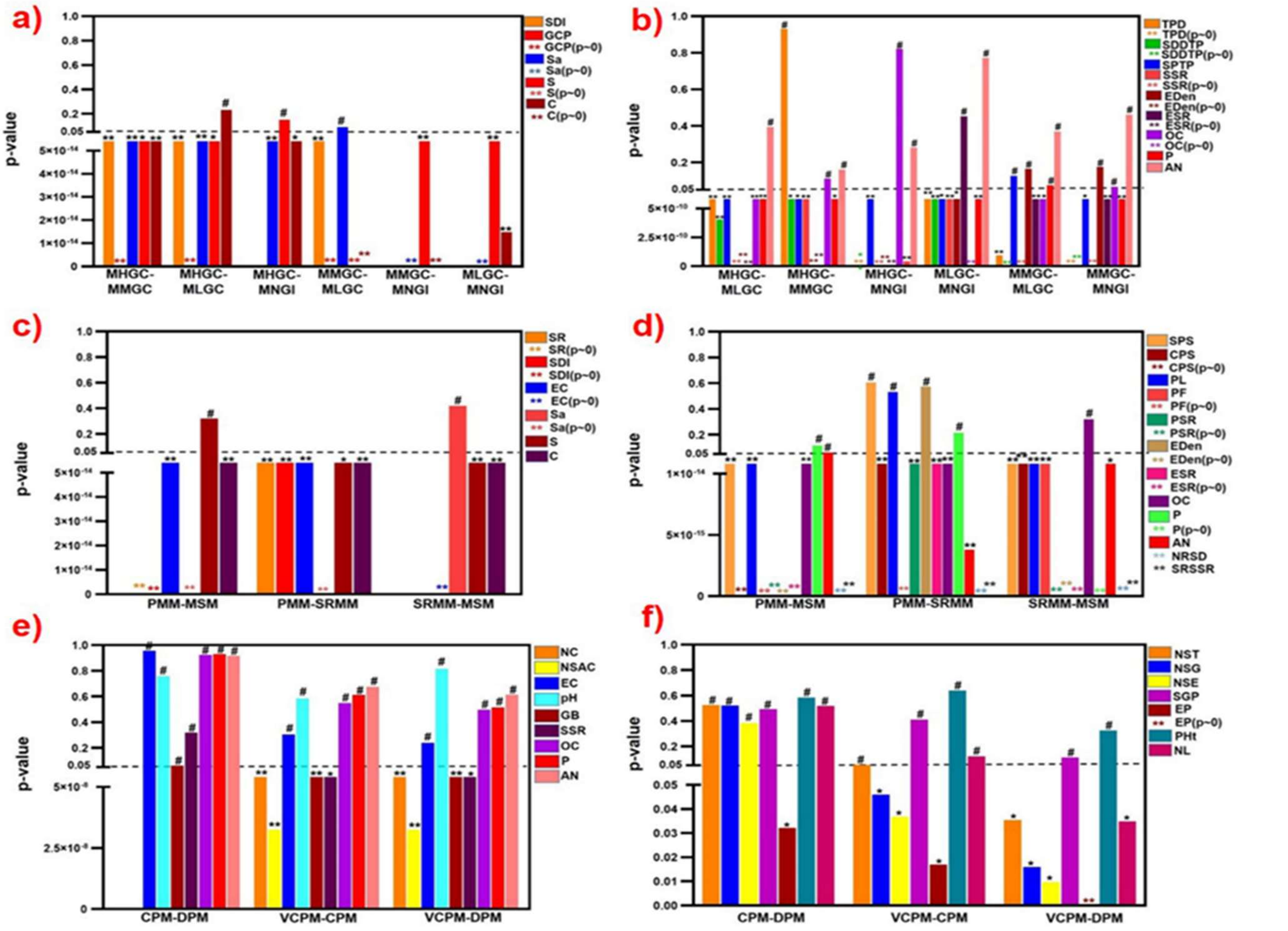

Supplementary Fig. 22A Bar diagram representing the *p* values based on Bayesian t-tests of equality of means with unequal variances a) Grass assisted stabilization with known variables, b) Grass assisted stabilization with unknown variables, c) Multispecies assemblage with known variables, d) Multispecies assemblage with unknown variables, e) Facilitative interaction with known variables and f) Facilitative interaction with unknown variables. (*) and (**) refer to the relevant null hypothesis that is not valid at 5% and 0.1% levels of significance, respectively. (#) refers to the relevant null hypothesis being valid above 5% level of significance. The horizontal dotted line represents the level of *p* value at 0.05 (5% level of significance) under consideration.

a) & b) TPD- Trapped propagules density, SDDTP- Seedling density developed from trapped propagule, SPTP- Survival % of trapped propagules, SSR- Seedlings species richness, Eden- Epifaunal density, ESR- Epifaunal species richness, OC- Organic carbon %, AN- Ammonia nitrogen, P- Phosphorus, MNGI- Monospecies with no grass at initial stage, MLGC- Multispecies with low grass coverage, MHGC- Multispecies with high grass coverage, MMGC- Multispecies with medium grass coverage, SDI- Shannon diversity index, GCP- Grass cover %, Sa- Sand %, S- Silt %, C- Clay %

c) & d) SPS- Self-pollination success, CPS- Cross-pollination success, PL- Pollen load, PF- Pollinator frequency, PSR- Pollinator species richness, Eden- Epifaunal Density, ESR- Epifaunal species richness, OC- Organic carbon %, P- Phosphorus, AN- Ammonia nitrogen, NRSD- Naturally regenerated seedling density, SRSSR- Naturally regenerated seedling species richness, MSM- Monospecies mangroves, SRMM- Semi-restored multispecies mangroves, PMM- Pristine multispecies mangroves, SR- Species richness, SDI- Shannon diversity index, EC- Electrical conductivity, Sa- Sand %, S- Silt %, C- Clay %

e) & f) NST- Number of seeds/seedlings transplanted, NSE- Number of seeds/seedlings established, NSG- Number of seeds germinated, SGP- Seeds germination %, SPES- Survival % of established seedlings, NL- Number of leaves, PHt- Present height, VCPM- Very closely planted mangrove seedlings (≤ 5cm.), CPM- Closely planted mangrove seedlings (≤ 40cm.), DPM- Distantly planted mangrove seedlings (≥100cm.), NC- Number of clumps, NSAC- No. of seeds/seedlings aggregated in a clump, EC- Electrical conductivity, GB- Glycine betaine, SSR- Soluble sugar-starch ratio, OC- Organic Carbon %, P- Phosphorus, AN- Ammonia-nitrogen

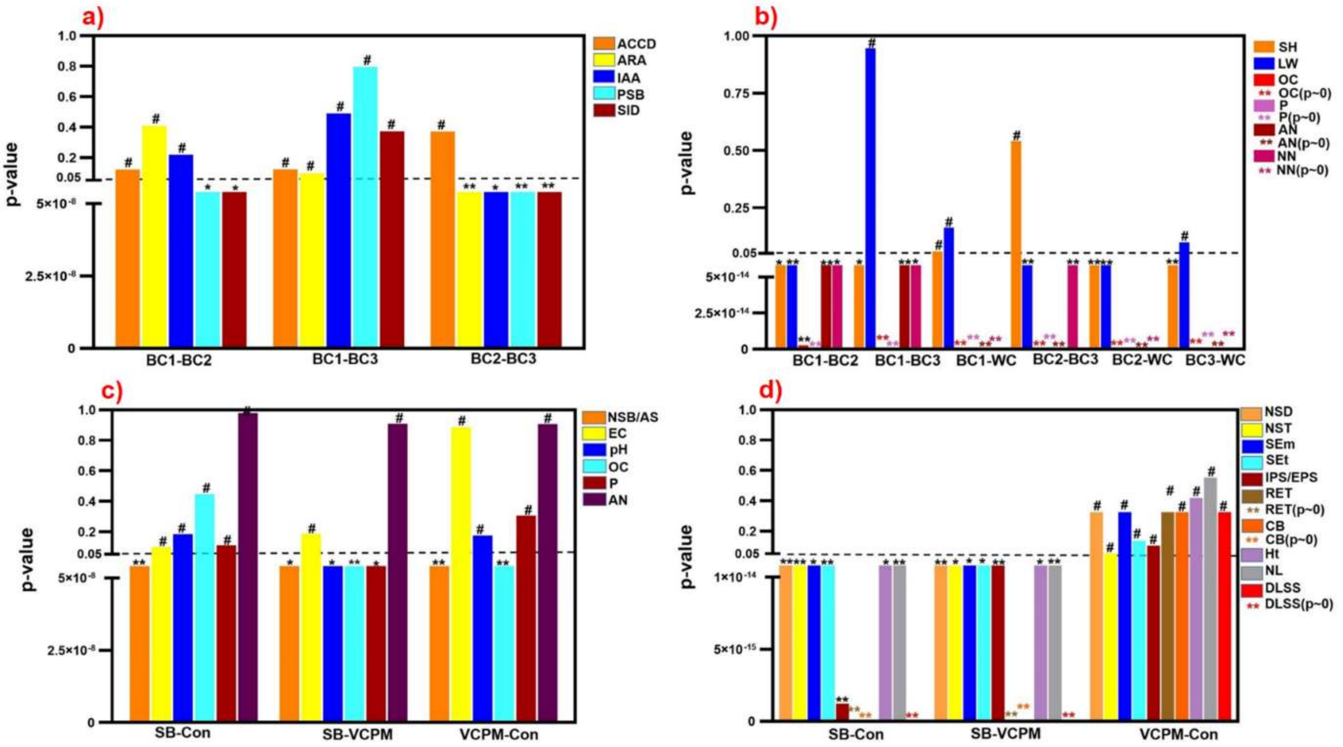

Supplementary Fig. 22 B Bar diagram representing the significance value (*p* value) using Bayesian test. a) Growth promotion by onsite PGPR consortia addition with known variables, b) Growth promotion by onsite PGPR consortia addition with unknown variables, c) Seed ball use with known variables, d) Seed ball use with unknown variables. (*) and (**) refers to the relevant null hypothesis that is not valid at 5% and 0.1% levels of significance, respectively. (#) refers to the relevant null hypothesis being valid at 5% level of significance. The horizontal dotted line represents the level of *p* value at 0.05 (5% level of significance) under consideration.

a) & b) PGPR-Plant growth promoting rhizobacteria, LW-Final leaf width, SH-Final shoot height, AN-Ammonia nitrogen, NN-Nitrate nitrogen, P-Phosphorus, OC-Organic carbon %, WC-Without consortium, BC1-BC1 consortium, BC2- BC2 consortium, BC3- BC3 consortium, SID- Siderophore %, IAA- Indole Acetic Acid Produced, PSB- P-solubilization, ACCD- ACC deaminase units, ARA- Acetylene Reduction assay

c) & d) NSD- Number of seed balls dispersed, NST- Number of seedlings transplanted, SEm- Number of seedlings emerged from seed balls, SEt- Number of seedlings established from transplanted seedlings, CB- Cost benefit, RET- Reduction in establishment time, Ht- Height of seedlings, NL- Number of leaves, DLSS- Decrease in use of less saline soil, IPS/EPS- Emergence % of seedlings from seed balls/ establishment % of transplanted seedlings, SB- seed ball technology, Con- Conventional technology, VCPM- Very closely planted mangrove

seedlings (≤ 5cm.), NSB- Number of seeds in a ball, EC- Electrical conductivity, OC-Organic Carbon %, AN-Ammonia-nitrogen, P- Phosphorus

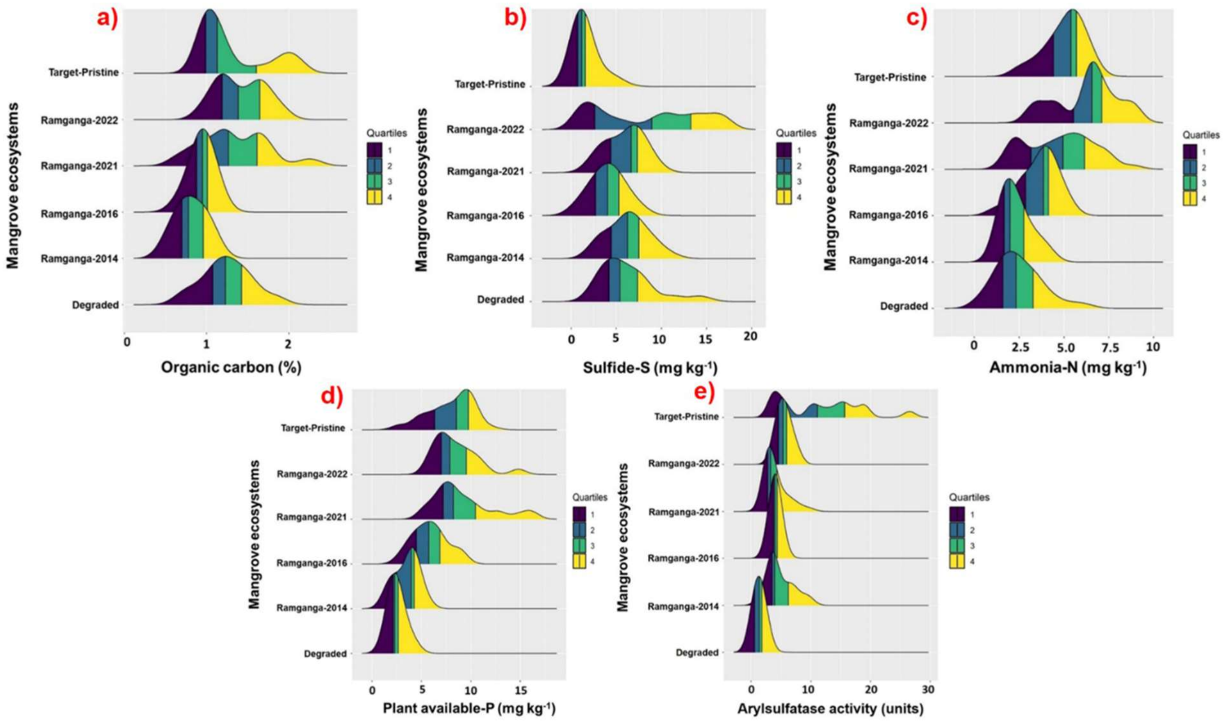

Supplementary Fig. 23A Ridgeline plots depicting the distributional changes of 5 major nutrient parameters of mangrove sediments across different states of mangroves viz. Degraded, Ramganga 2014, Ramganga 2016, Ramganga 2021, Ramganga 2022 and Target pristine reference mangrove forest (Y axis) a) Organic carbon%, b) Sulfide-S (mg Kg^{-1}), c) Ammonia-N (mg Kg^{-1}), d) Plant available-P (mg Kg^{-1}), e) Aryl sulfatase activity (units). Here Ramganga site is the semi-restored site that was under restoration efforts since 2014.

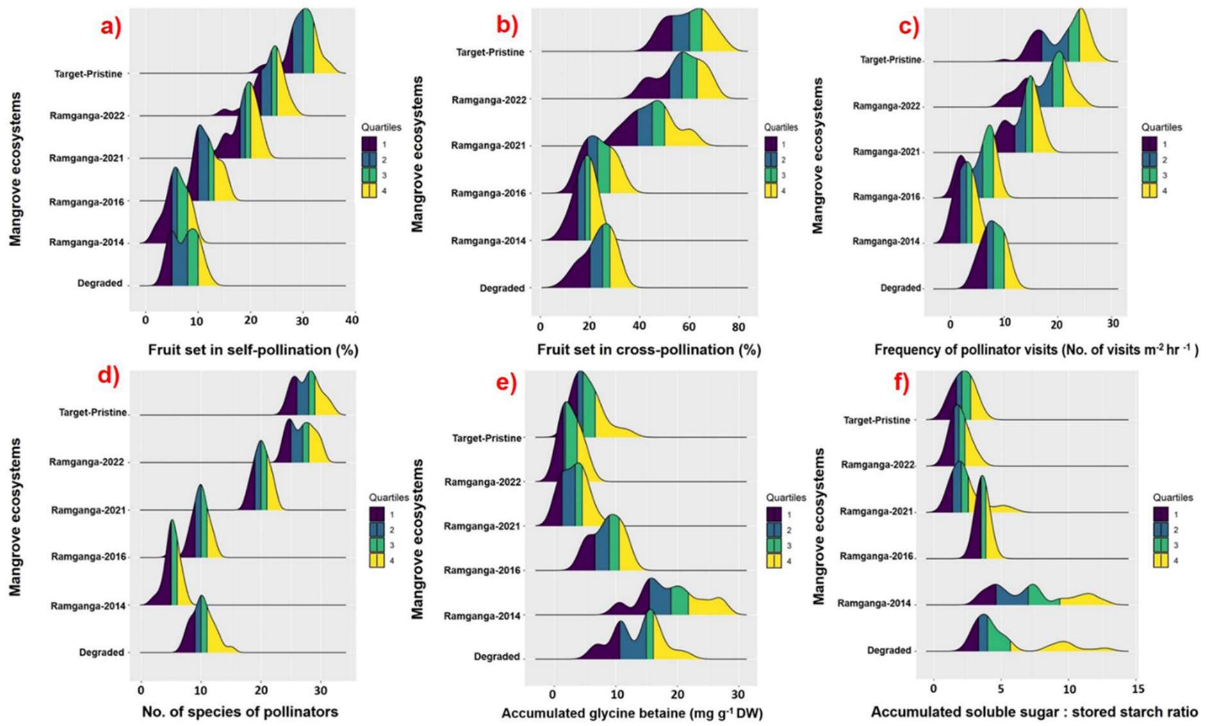

Supplementary Fig. 23B Ridgeline plots depicting the distributional changes of reproductive outcomes and osmotic resilience across different states of mangroves viz. Degraded, Ramganga 2014, Ramganga 2016, Ramganga 2021, Ramganga 2022 and Target pristine reference mangrove forest (Y axis) a) Successful fruit set in self-pollination%, b) Successful fruit set in cross-pollination%, c) Frequency of pollinator visits (no. of visits $m^{-2}hr^{-1}$), d) Number of species of pollinators, e) Accumulated glycine betaine (mg g^{-1} DW), f) Accumulated soluble:starch ratio. Here Ramganga site is the semi-restored site that was under restoration efforts since 2014.

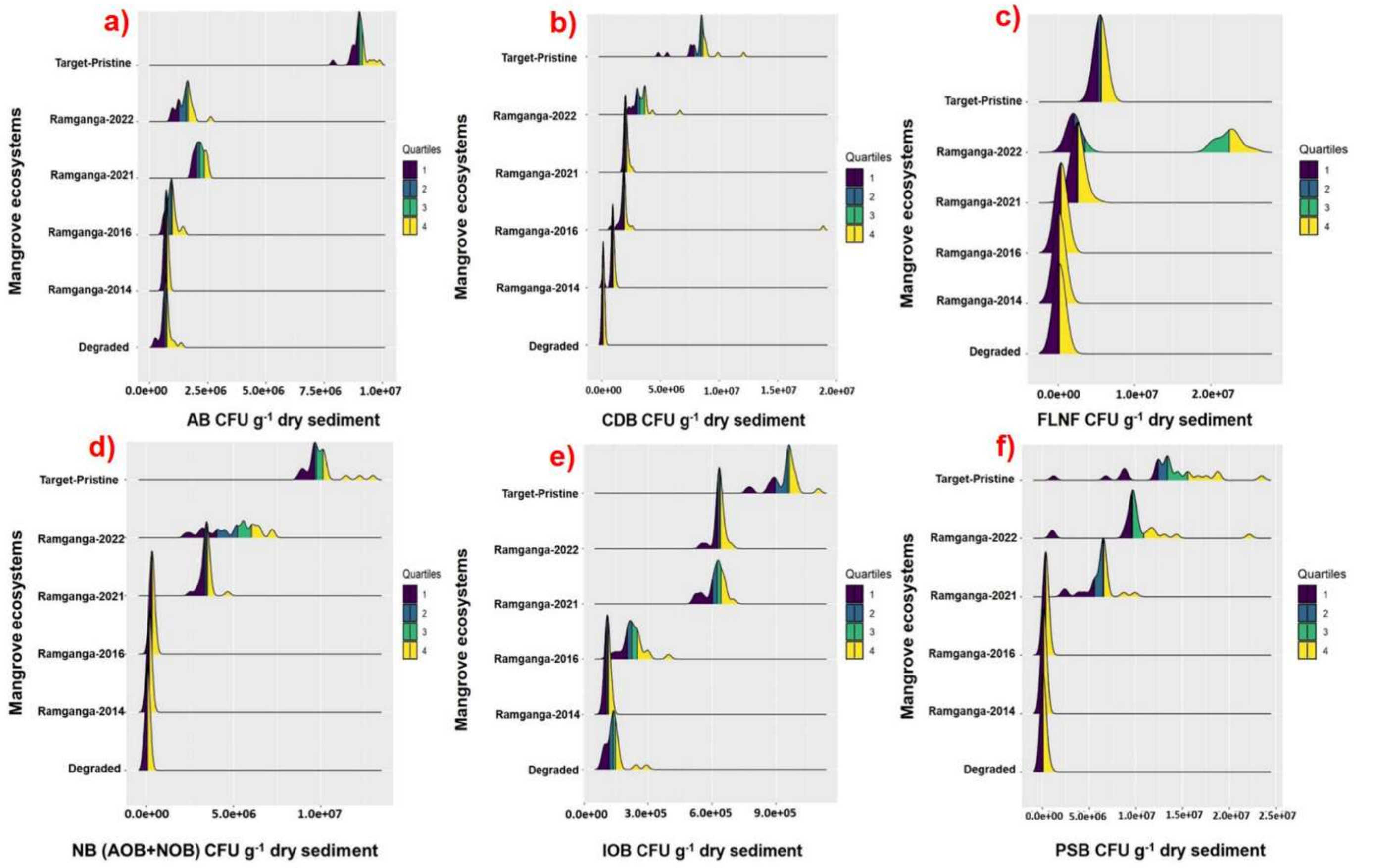

Supplementary Fig. 23C Ridgeline plots depicting the distributional changes of 5 different physical criteria of mangrove sediments across different states of mangroves viz. Degraded, Ramganga 2014, Ramganga 2016, Ramganga 2021, Ramganga 2022 and Target pristine reference mangrove forest (Y axis) a) Colony forming unit (CFU) of nutrient cycling AB bacteria, b) Colony forming unit (CFU) of nutrient cycling CDB bacteria, c) Colony forming unit (CFU) of nutrient cycling FLNF bacteria, d) Colony forming unit (CFU) of nutrient cycling NB bacteria, e) Colony forming unit (CFU) of nutrient cycling IOB bacteria, f) Colony forming unit (CFU) of nutrient cycling PSB bacteria. Here n=25.

AB=Ammonifying bacteria, CDB=Cellulose degrading bacteria, FLNF= Free living nitrogen fixing bacteria, NB= Nitrifying bacteria, IOB= Iron oxidizing bacteria, PSB= Phosphorous solubilizing bacteria

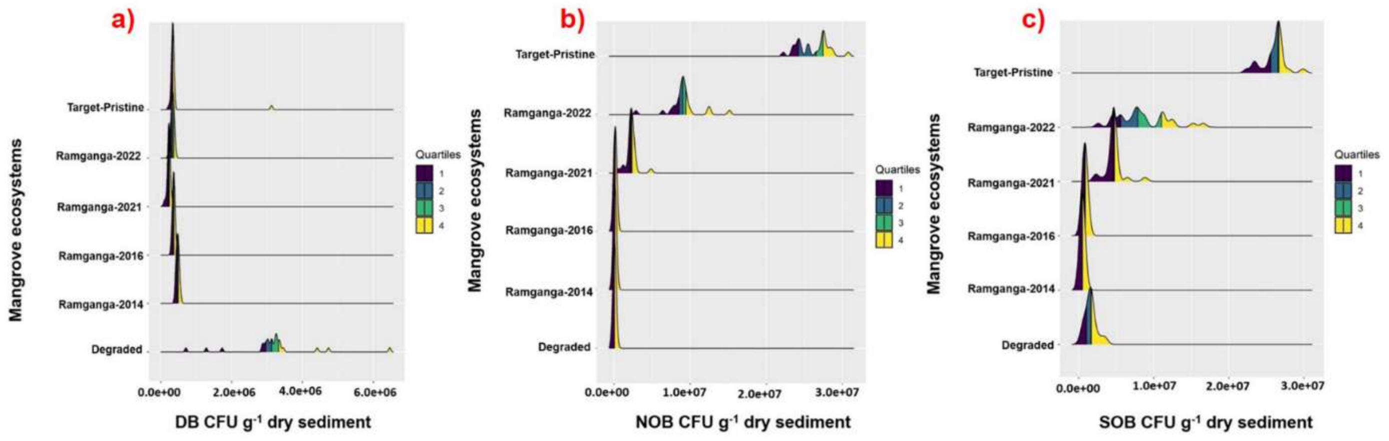

Supplementary Fig. 23D Ridgeline plots depicting the distributional changes of 5 different physical criteria of mangrove sediments across different states of mangroves viz. Degraded, Ramganga 2014, Ramganga 2016, Ramganga 2021, Ramganga 2022 and Target pristine reference mangrove forest (Y axis) a) Colony forming unit (CFU) of nutrient cycling DB bacteria, b) Colony forming unit (CFU) of nutrient cycling NOB bacteria, c) Colony forming unit (CFU) of nutrient cycling SOB bacteria. Here n=25.

DB= Denitrifying bacteria, NOB= Nitrite oxidizing bacteria, SOB= Sulphur oxidizing bacteria

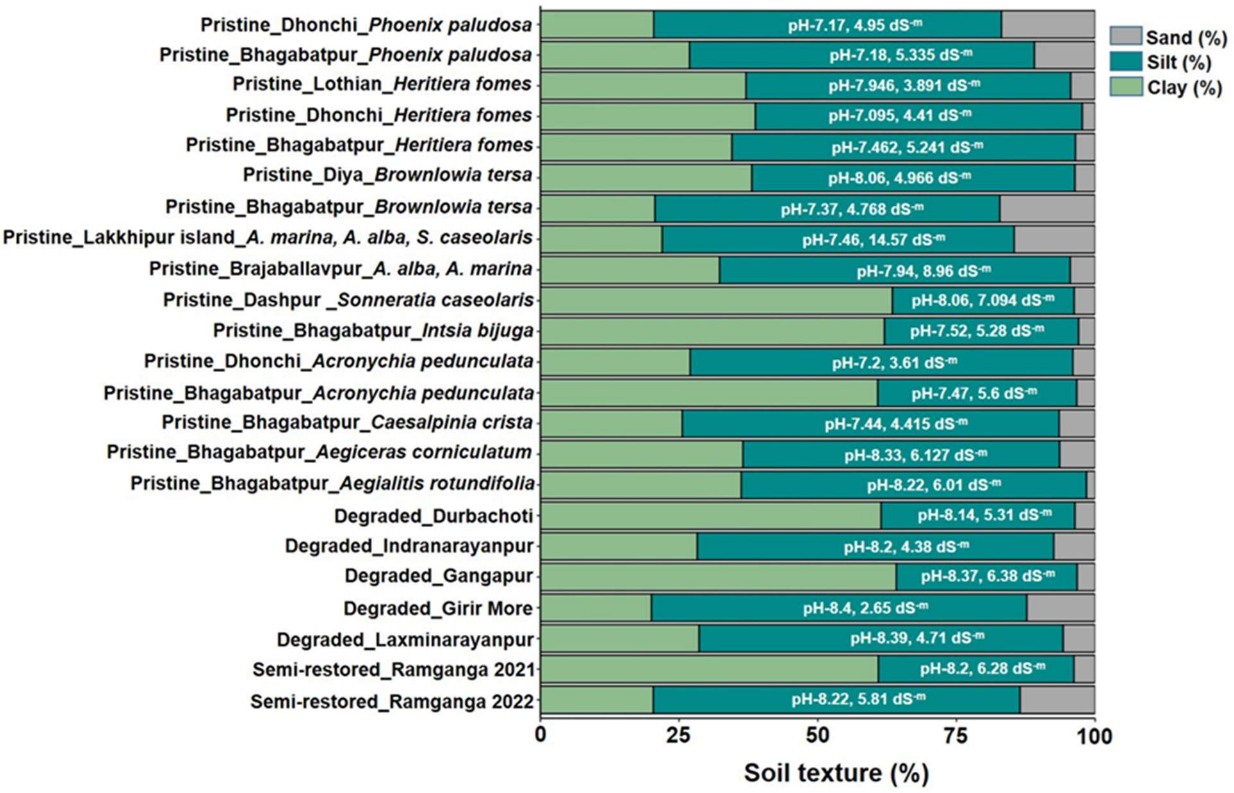

Supplementary Fig. 24 Species-specific physical features of rhizosphere sediments from naturally colonized stands in pristine mangroves at Indian Sundarbans vis-à-vis that of sediments of our ongoing sites of restoration. *Phoenix paludosa*, *Heritiera fomes*, *Brownlowia tersa*, *Intsia bijuga*, *Acronychia pedaunculata*, *Caesalpinia crista* and *Aegialitis rotundifolia* are rare and threatened species (IUCN declared), while *Avicennia marina*, *Avicennia alba*, *Sonneratia caseolaris* and *Aegiceras corniculatum* are some common mangrove species at Indian Sundarbans. Degraded Durbachoti, Degraded Indranarayanpur, Degraded Gangapur, Degraded Giri More, Degraded Laxminaraynpur, Semi-restored Ramganga 2021 and Semi-restored Ramganga 2022 are our ongoing sites of restoration. An average value of sand%, silt%, clay%, pH, and salinity from composite soil samples is represented for each horizontal bar with n=7.

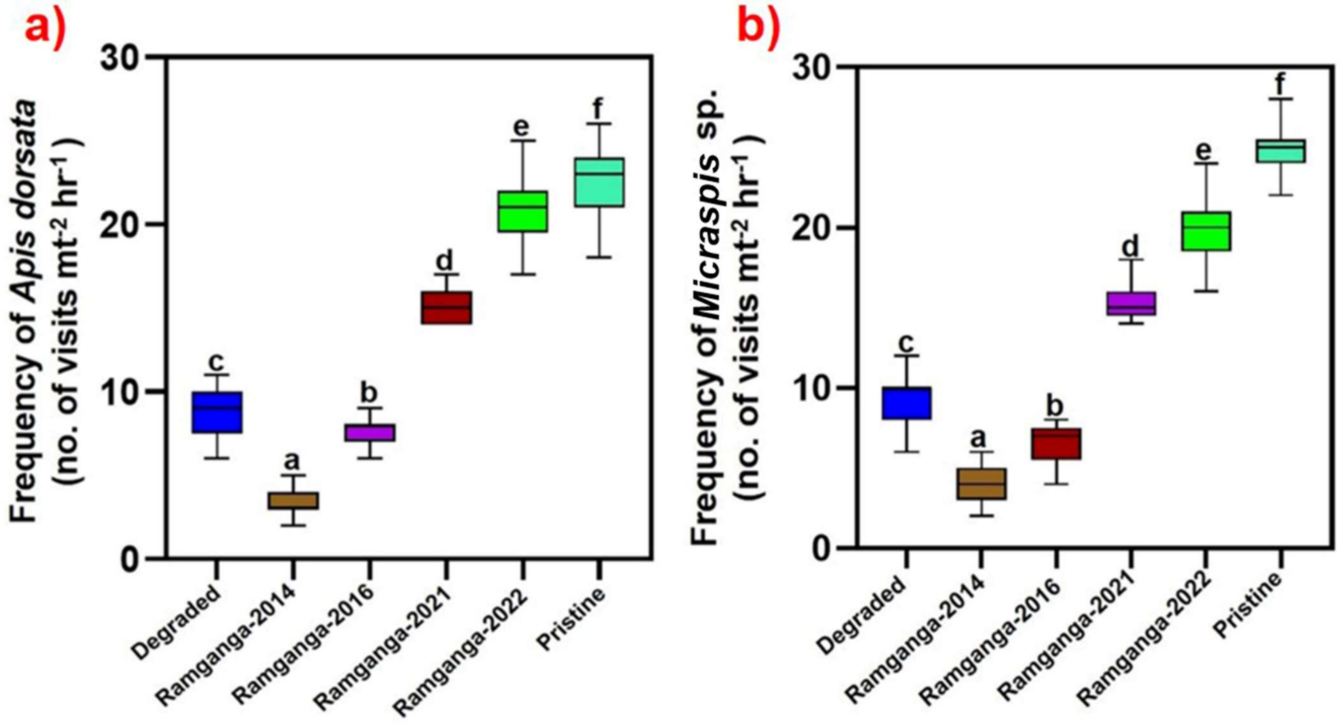

Supplementary Fig. 25 Box-whisker plot displaying the Frequency of visitation by two primary generalist pollinators between the experimental sites Degraded, Ramganga 2014, Ramganga 2016, Ramganga 2021, Ramganga 2022 and Pristine reference mangrove forest a) Frequency of *Apis dorsata* , b) Frequency of *Micraspis* sp. Whiskers represent the range of data from highest to lowest with a median value. Here n = 25. Values designated with different letters are significantly different at the 5% level.

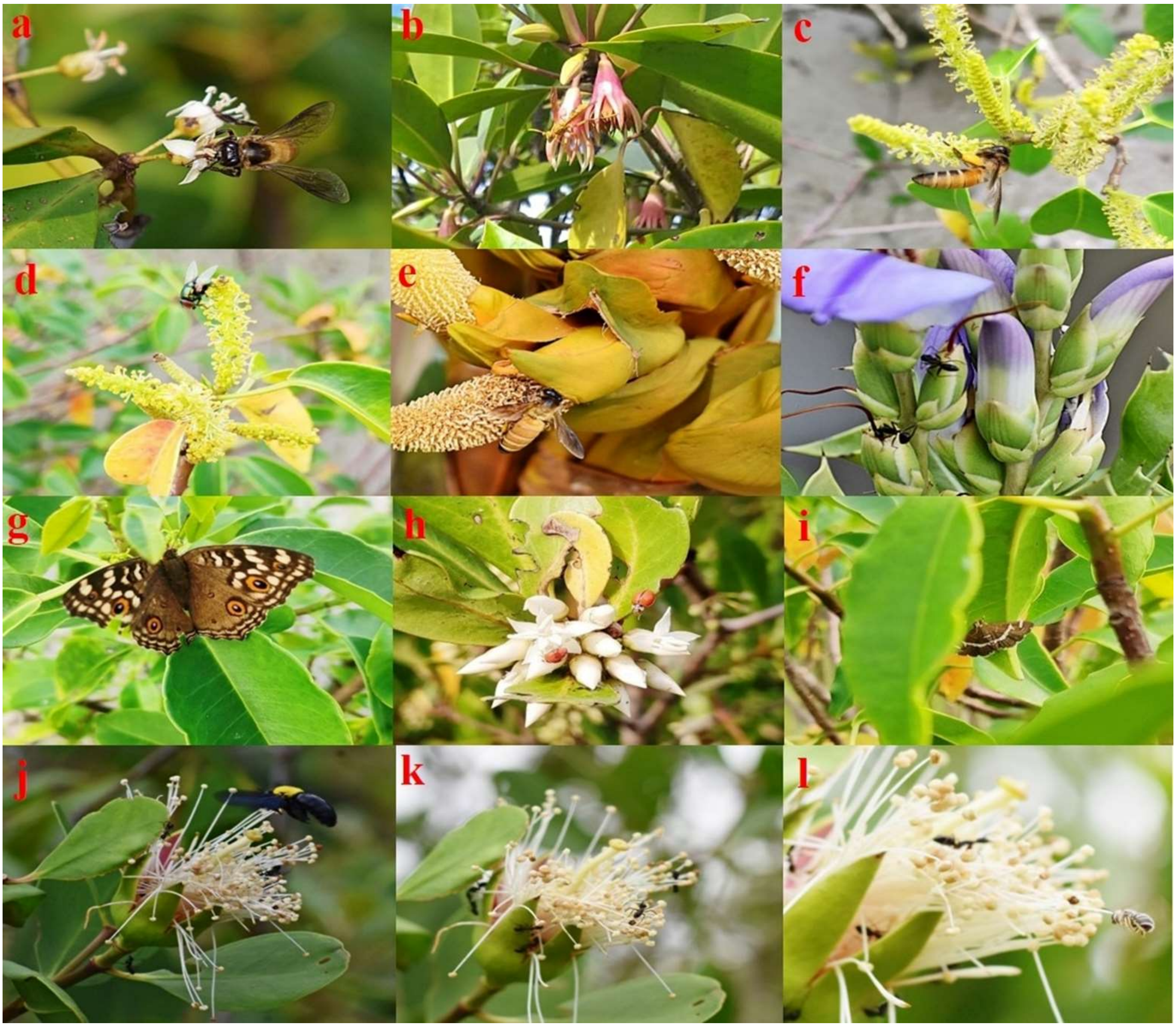

Supplementary Fig. 26 Diverse pollinators at present on different mangroves at the site of restoration (a) *Apis dorsata* on *Aegiceras corniculatum*, (b) *Polistes* sp. on *Bruguiera gymnorrhiza*, (c) *Apis dorsata* on *Excoecaria agallocha*, (d) *Chrysomya rufifacies/albiceps* on *Excoecaria agallocha*, (e) *Apis dorsata* on *Nypa fruticans*, (f) *Camponotus* sp. on *Acanthus ilicifolius*, (g) *Junonia lemonias* on *Excoecaria agallocha*, (h) *Micraspis* sp. on *Aegiceras corniculatum*, (i) *Spoladea recurvalis* on *Excoecaria agallocha,* (j) *Xylocopa* c.f. *aestuans* on *Sonneratia caseolaris,* (k) *Camponotus* sp. on *Sonneratia caseolaris,* (l) *Halictus* sp. */ Lasioglossum* sp. on *Sonneratia caseolaris*

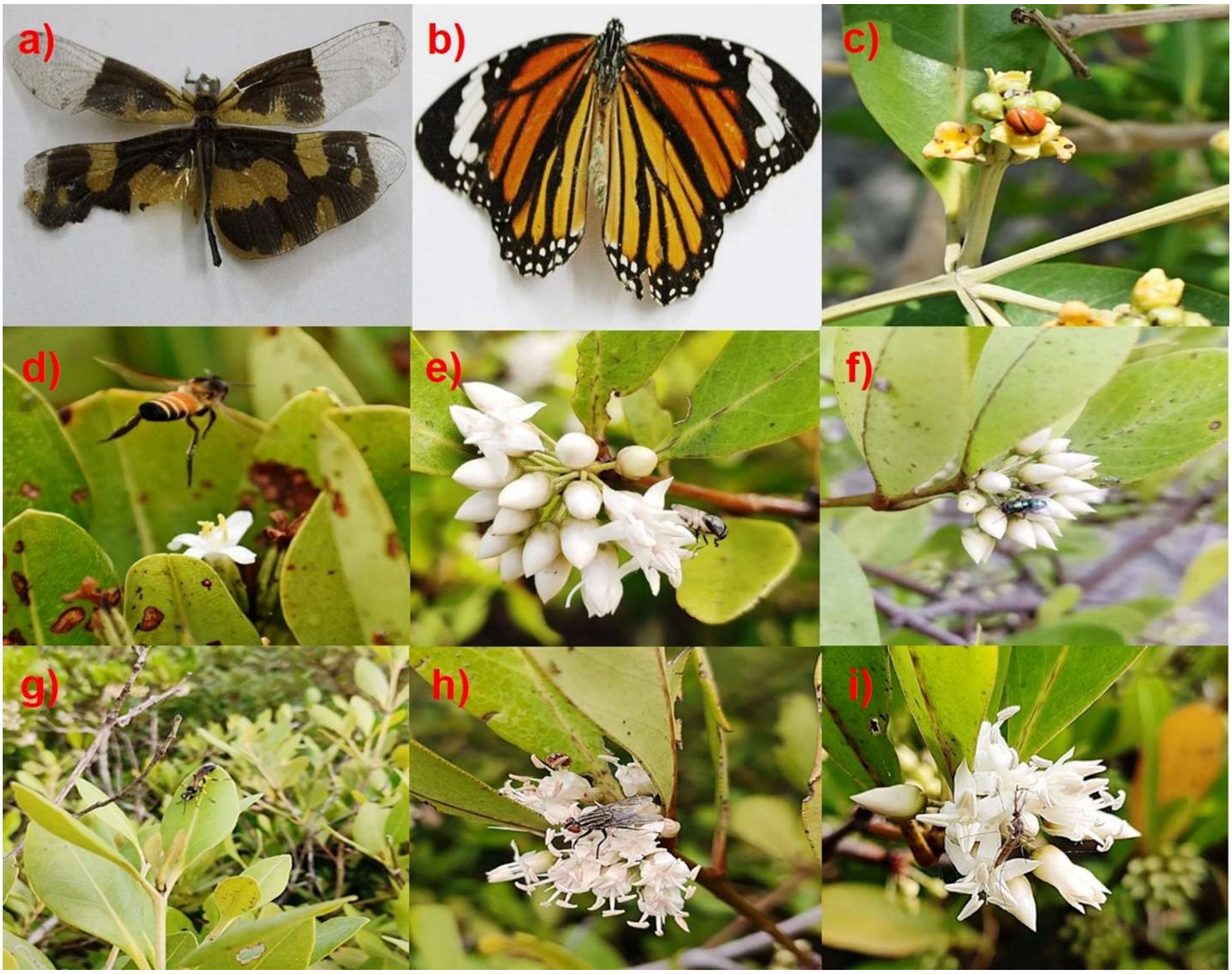

Supplementary Fig. 27 Diverse pollinators at present on different mangroves at the site of restoration (a) *Rhyothemis variegata* on *Excoecaria agallocha*, (b) *Danaus chrysippus* on *Excoecaria agallocha*, (c) *Micraspis discolor* on *Avicennia* spp., (d) *Apis dorsata* on *Aegialitis rotundifolia*, (e) *Stomorhina c.f. lunata* on *Aegiceras corniculatum*, (f) *Lucilia* sp. on *Aegiceras corniculatum*, (g) *Sceliphron* sp. on *Avicennia* spp., (h) *Sarcophaga* sp. on *Aegiceras corniculatum*, (i) *Tipulidae* (member of the family) on *Aegiceras corniculatum*

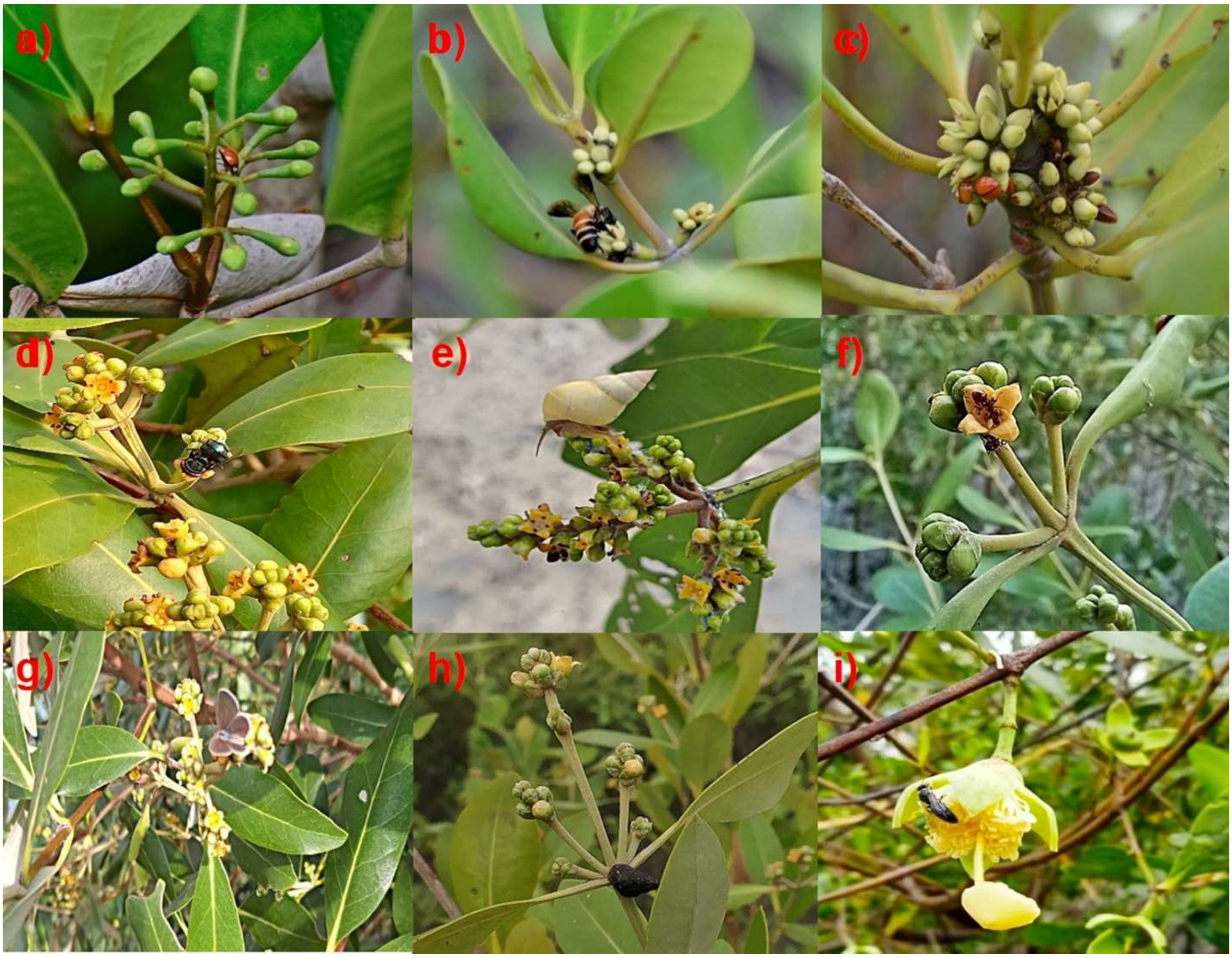

Supplementary Fig. 28 Diverse pollinators at present on different mangroves at the site of restoration (a) *Micraspis* sp. on *Xylocarpus mekongensis*, (b) *Apis dorsata* on *Ceriops decandra*, (c) *Micraspis* sp. on *Ceriops decandra*., (d) *Chrysomya megacephala* on *Avicennia* spp., (e) *Littoraria melanostoma* on *Avicennia* spp., (f) *Oecophylla* sp.on *Avicennia* spp., (g) *Euchrysops* sp. on *Avicennia* spp., (h) *Littoraria* sp. (Variant 2) on *Avicennia* spp., (i) *Nomia* sp. on *Sonneratia apetala*

Supplementary Table 5: Pollinator types and their frequency of visits at the site of restoration (2014-22)

Serial No.	Name of the mangrove species	Name of the pollinating agent	Common name	Systematic position	Frequency of pollinator visit (No. of visits mt^{-2} hr^{-1})	
					2014	2022
**1.**	***Aegiceras corniculatum***	*Apis dorsata*	Honey bee	Order: Hymenoptera<br>Family: Apidae	3	12
**2.**		*Micraspis* sp.	Lady Bird Beetle	Order: Coleoptera<br>Family: Coccinelidae	2	15
**3.**		*Stomorhina* c.f. *lunata*	Locust Blowfly	Order: Diptera<br>Family: Rhiniidae	0	6
**4.**		*Lucilia* sp.	Blow flies	Order: Diptera<br>Family: Calliphoridae	0	6
**5.**		*Sarcophaga* sp	Common flesh flies	Order: Diptera<br>Family:Sarcophagidae	0	3
**6.**		Tipulidae (member of the family)	Crane fly	Order: Diptera<br>Family:Tipulidae	0	7
**7.**	***Excoecaria agallocha***	*Apis dorsata*	Honey bee	Order: Hymenoptera<br>Family: Apidae	3	15
**8.**		*Chrysomya rufifacies/ albiceps*	Hairy Maggot Blow flies	Order: Diptera<br>Family: Calliphoridae	0	4
**9.**		*Junonia lemonias*	Nymphalid butterfly	Order: Lepidoptera<br>Family: Nymphalidae	0	6
**10.**		*Spoladea recurvalis*	Beet Webworm Moth	Order: Lepidoptera<br>Family: Crambidae	0	5
**11.**		*Micraspis discolor*	Lady Bird Beetle	Order: Coleoptera<br>Family: Coccinelidae	3	10
**12.**		*Rhyothemis variegata*	Dragon Fly (Flutterer)	Oder : Odonata<br>Family: Libellulidae	0	4

**13.**	***Excoecaria agallocha***	*Danaus chrysippus*	African Monarch Butterfly	Order: Lepidoptera<br>Family: Nymphalidae	0	2
**14.**		*Anoplognathus* sp.	Christmas beetle (yellow coloured)	Order: Coleoptera<br>Family: Scarabaeidae	0	6
**15.**	***Aegialitis rotundifolia***	*Apis dorsata*	Honey bee	Order: Hymenoptera<br>Family: Apidae	2	15
**16**		*Micraspis discolor*	Lady Bird Beetle	Order: Coleoptera<br>Family: Coccinelidae	4	10
**17.**	***Bruguiera gymnorrhiza***	*Polistes* sp.	Yellow wasp	Order: Hymenoptera<br>Family: Vespidae	0	6
**18.**		*Apis dorsata*	Honey bee	Order: Hymenoptera<br>Family: Apidae	0	12
**19.**	***Acanthus ilicifolius***	*Camponotus* sp.	Carpenter ants	Order: Hymenoptera<br>Family: Formicidae	3	11
**20.**	***Volkameria inermis***	*Chrysocoris* sp.	Jewel Bug	Order: Hemiptera<br>Family: Scutelleridae	0	4
**21.**	***Avicennia* spp.**	*Micraspis discolor*	Lady Bird Beetle	Order: Coleoptera<br>Family: Coccinelidae	5	12
**22.**		*Sceliphron* sp.	Mud dauber wasp	Order:Hymenoptera<br>Family:Sphecidae	0	4
**23.**		*Apis dorsata*	Honey bee	Order: Hymenoptera<br>Family: Apidae	3	12
**24.**		*Junonia lemonias*	Nymphalid butterfly	Order: Lepidoptera<br>Family: Nymphalidae	0	4
**25.**		*Chrysomya megacephala*	Oriental bluefly	Order: Diptera<br>Family: Calliphoridae	0	2

**26.**	**_Avicennia_ spp.**	*Euchrysops* sp.	Butterfly	Order: Lepidoptea<br>Family: Lycaenidae	0	1
**27.**		*Camponotus* sp.	Carpenter ants	Order: Hymenoptera<br>Family: Formicidae	0	2
**28.**		*Oecophylla* sp.	Weaver ant	Order: Hymenoptera<br>Family: Formicidae	0	3
**29.**		*Hylaeus c.f. strenuou*	Masked bees	Order: Hymenoptera<br>Family: Colletidae	0	4
**30.**		*Littoraria melanostoma*	Sea snail	Order: Littorinimorpha<br>Family: Littorinidae	0	2
**31.**		*Littoraria* sp.*(variant 2)*	Sea snail	Order: Littorinimorpha<br>Family: Littorinidae	0	3
**32.**		*Littoraria* sp.*(variant 3)*	Sea snail	Order: Littorinimorpha<br>Family: Littorinidae	0	2
**33.**	***Nypa fruticans***	*Apis dorsata*	Honey bee	Order: Hymenoptera<br>Family: Apidae	0	6
**34.**	***Ceriops decandra***	*Apis dorsata*	Honey bee	Order: Hymenoptera<br>Family: Apidae	0	10
**27.**		*Micraspis* sp.	Lady Bird Beetle	Order: Coleoptera<br>Family: Coccinelidae	0	12
**35.**	***Xylocarpus mekongensis***	*Micraspis* sp.	Lady Bird Beetle	Order: Coleoptera<br>Family: Coccinelidae	0	6
**36.**	***Bruguiera cylindrica***	*Micraspis discolor*	Lady Bird Beetle	Order: Coleoptera<br>Family: Coccinelidae	2	9
**37.**		*Apis dorsata*	Honey bee	Order: Hymenoptera<br>Family: Apidae	0	6

**38.**	***Sonneratia apetala***	*Apis dorsata*	Honey bee	Order: Hymenoptera<br>Family: Apidae	0	8
**39.**		*Hylaeus strenuous*	Bees	Order: Hymenoptera<br>Family: Colletidae	0	3
**40.**		*Micraspis discolor*	Lady Bird Beetle	Order: Coleoptera<br>Family: Coccinelidae	2	10
**41.**		*Nomia* sp.	Sweet bees	Order: Hymenoptera<br>Family: Halictidae	0	5
**42.**	***Sonneratia caseolaris***	*Xylocopa* c.f. *aestuans*	Carpenter bees	Order: Hymenoptera<br>Family: Apidae	0	4
**43.**		*Halictus* sp. / *Lasioglossum* sp.	Sweat bees	Order: Hymenoptera<br>Family: Halictidae	0	5
**44.**		*Camponotus* sp.	Carpenter ants	Order: Hymenoptera<br>Family: Formicidae	0	8

Supplementary Table 6: Dominant epifaunal diversity at the site of restoration

Crab species found at the site of restoration (Ramganga) in 2021-2022

Sl no.	Species name
1	*Episesarma mederi* (Mangrove tree climbing crab)
2	*Clibanarius vittatus* (Yellow thin striped Hermit Crab)
3	*Metaplax crenulata* (Mudflat varunid crab)
4	*Scylla serrata* (Mud crab)
5	*Uca (Tubuca) rosea* (Red Fiddler crab)
6	*Carcinoscorpius rotundicauda* (Mangrove horseshoe crab)

Molluscan members found at the site of restoration (Ramganga) in 2021-2022

Sl no.	Species name
1	*Anadara* spp. (Granular Ark Padma Jhinuk)
2	*Bufonaria* spp. (Common frog shell)
3	*Bursa spinosa* (Spiny frog shell)
4	*Cerithidia* spp.
5	*Cerithidia cingulata* (Horn shell)
6	*Littoraria* spp.
7	*Littoraria melanostoma* (Periwinkles)
8	*Enigmonia* spp.
9	*Telescopium* spp.
10	*Neripteron violaceum*
11	*Nerita articulata*
12	*Nerita* spp.
13	*Neritina natalensis* (Red spotted nerite snail)
14	*Pugilina cochlidium* (brown)
15	*Pugilina cochlidium* (spiral melongena)
16	*Thiara scabra*
17	*Littorina* sp.

Mudskipper varieties found at the site of restoration (Ramganga) in 2021-2022

Sl no.	Species name
1	*Periophthalmus kalolo* (common mudskipper)
2	*Periophthalmus* spp.
3	*Boleophthalmus boddarti*
4	*Periophthalmodon* spp.

Supplementary Table 7: Seed ball use data from four experimental sites of restoration

Name of the degraded site	Coordinates	Name of the seed balls	Number of seed balls	Number of seeds per ball	Number of survived seedlings	Survival percentage (%)
Indranarayanpur 2	21° 56.277'N/ 88° 20.878'E	*Heritiera fomes*	5000	1	1400	28.00
		Brownlowia tersa	3600	4-6	1300	36.11
		Phoenix paludosa	4500	6-7	1900	42.22
Durbachoti 1	21° 51.228'N/ 88° 18.735'E	*Heritiera fomes*	1000	1	460	46.00
		Phoenix paludosa	2000	6-7	1150	57.50
Daxinlaxminarayan pur 1	21° 45.743'N/ 88° 20.677'E	*Heritiera fomes*	1300	1	500	38.46
		Phoenix paludosa	11000	6-7	6400	58.18
		Brownlowia tersa	3000	1-3	600	20.00
Gangapur 3	21° 45.200'N /88° 24.115'E	*Heritiera fomes*	2300	1	1100	47.82
		Phoenix paludosa	12000	6-7	5500	45.83
		**Total**	**45700**		**20310**	**44.44**

Supplementary Table 8: Naturally recruited seedlings showing self-sustenance of ecosystem at Ramganga restored site at 2022, Daspur and Bhagabatpur pristine reference mangrove site, and *Ceriops tagal* monotypic plantation site at Atharogazi and *Bruguiera gymnorrhiza* monotypic plantation site at Durbachoti

**Naturally regenerated seedling in the begining of restoration at Ramganga 2014**	**Naturally regenerated seedling species present at Ramganga in 2022**	**Naturally regenerated seedling species present at Daspur pristine mangrove**	**Naturally regenerated seedling species present at Bhagabatpur pristine mangrove**	**Naturally regenerated seedling species present at Durbachoti monotypic plantation site**	**Naturally regenerated seedling species present at Atharogaji monotypic plantation site**
Acanthus ilicifolius	*Acanthus ilicifolius*	*Acanthus ilicifolius*	*Acanthus ilicifolius*	*Aegiceras corniculatum*	*Bruguiera cylindrica*
Aegialitis rotundifolia	*Aegialitis rotundifolia*	*Aegialitis rotundifolia*	*Aegialitis rotundifolia*	*Bruguiera gymnorrhiza*	*Ceriops decandra*
Aegiceras corniculatum	*Aegiceras corniculatum*	*Aegiceras corniculatum*	*Aegiceras corniculatum*	*Bruguiera parviflora*	*Ceriops tagal*
Avicennia spp.	*Avicennia spp.*	*Merope angulata*	*Avicennia spp.*		
Excoecaria agallocha	*Bruguiera cylindrica*	*Avicennia spp.*	*Bruguiera cylindrica*		
	Bruguiera gymnorrhiza	*Bruguiera cylindrica*	*Bruguiera gymnorrhiza*		
	Bruguiera parviflora	*Bruguiera gymnorrhiza*	*Ceriops decandra*		
	Ceriops decandra	*Bruguiera parviflora*	*Ceriops tagal*		
	Dalbergia spinosa	*Ceriops decandra*	*Dalbergia spinosa*		
	Derris trifoliata	*Ceriops tagal*	*Derris trifoliata*		
	Excoecaria agallocha	*Dalbergia spinosa*	*Excoecaria agallocha*		
	Sonneratia sp	*Derris trifoliata*	*Finlaysonia obovata*		
		Excoecaria agallocha	*Heritiera fomes*		
		Finlaysonia obovata	*Phoenix paludosa*		
		Heritiera fomes	*Sonneratia sp*		
		Sonneratia sp	*Xylocarpus sp*		
		Xylocarpus sp			

Supplementary Table 9: Results of redundancy analysis including F-statistics and *p*-values derived from permutation tests

9.1 Grass assisted stabilization

Response variables (Y)	Explanatory Variables (Qualitative)	Explanatory Variables (Quantitative)	Inertia			Results of the permutation test		
				Value	%	Permutations	Pseudo F	*p*-value
SDI, GCP, Sa, S, C	MHGC, MMGC, MLGC MNGI	TPD, SDDTP, SPTP, SSR, EDen, ESR, OC, AN, P	Total	5.000	100.000	500	5.036	**< 0.0001**
			Constrained	4.172	83.433			
			Unconstrained	0.828	16.567			

TPD- Trapped propagules density, SDDTP- Seedling density developed from trapped propagule, SPTP- Survival % of trapped propagules, SSR- Seedlings species richness, Eden- Epifaunal density, ESR- Epifaunal species richness, OC- Organic carbon %, AN- Ammonia nitrogen, P- Phosphorus, MNGI- Monospecies with no grass at initial stage, MLGC- Multispecies with low grass coverage, MHGC- Multispecies with high grass coverage, MMGC- Multispecies with medium grass coverage, SDI- Shannon diversity index, GCP- Grass cover %, Sa- Sand %, S- Silt %, C- Clay %

9.2 Multispecies assemblage

Response variables (Y)	Explanatory Variables (Qualitative)	Explanatory Variables (Quantitative)	Inertia			Results of the permutation test		
				Value	%	Permutations	Pseudo F	*p*-value
SR,SDI,EC, Sa,S,C	MSM SRMM PMM	SPS, CPS, PL, PF, PSR, EDen, ESR, OC, P, AN, NRSD, SRSSR	Total	6.000	100.000	500	9.815	**< 0.0001**
			Constrained	5.445	90.753			
			Unconstrained	0.555	9.247			

SPS- Self-pollination success, CPS- Cross-pollination success, PL- Pollen load, PF- Pollinator frequency, PSR- Pollinator species richness, Eden- Epifaunal Density, ESR- Epifaunal species richness, OC- Organic carbon %, P- Phosphorus, AN- Ammonia nitrogen, NRSD- Naturally regenerated seedling density, SRSSR- Naturally regenerated seedling species richness, MSM- Monospecies mangroves, SRMM- Semi-restored multispecies mangroves, PMM- Pristine multispecies mangroves, SR- Species richness, SDI- Shannon diversity index, EC- Electrical conductivity, Sa- Sand %, S- Silt %, C- Clay %

9.3 Facilitative interaction

**Response variables (Y)**	**Explanatory Variables (Qualitative)**	**Explanatory Variables (Quantitative)**	**Inertia**			**Results of the permutation test**		
				Value	%	Permutations	Pseudo F	*p*-value
NC, NSAC, EC, pH, GB, SSR, OC, P, AN	VCPM CPM DPM	NST, NSE, NSG, SGP, SPES, PHt, NL	Total	9.000	100.000	500	0.595	**< 0.0001**
			Constrained	3.356	37.288			
			Unconstrained	5.644	62.712			

NST- Number of seeds/seedlings transplanted, NSE- Number of seeds/seedlings established, NSG- Number of seeds germinated, SGP- Seeds germination %, SPES- Survival % of established seedlings, NL- Number of leaves, PHt- Present height, VCPM- Very closely planted mangrove seedlings (≤ 5cm.), CPM- Closely planted mangrove seedlings (≤ 40cm.), DPM- Distantly planted mangrove seedlings (≥100cm.), NC- Number of clumps, NSAC- No. of seeds/seedlings aggregated in a clump, EC- Electrical conductivity, GB- Glycine betaine, SSR- Soluble sugar-starch ratio, OC-Organic Carbon %, P- Phosphorus, AN- Ammonia nitrogen

9.4 Growth promotion by onsite PGPR consortia addition

Response variables (Y)	Explanatory Variables (Qualitative)	Explanatory Variables (Quantitative)	Inertia			Results of the permutation test		
IAA, PSB, ACCD, SID, ARA	BC1, BC2, BC3, WC	LW, SH, AN, NN, P, OC		Value	%	Permutations	Pseudo F	*p*-value
			Total	5.000	100.000	500	0.897	**< 0.0001**
			Constrained	2.365	47.293			
			Unconstrained	2.635	52.707			

LW-Final leaf width, SH-Final shoot height, AN-Ammonia nitrogen, NN-Nitrate nitrogen, P-Phosphorus, OC-Organic carbon %, WC-Without consortium, BC1- BC1 consortium, BC2- BC2 consortium, BC3- BC3 consortium, SID- Siderophore %, IAA- Indole Acetic Acid Produced, PSB- P-solubilization, ACCD- ACC deaminase units, ARA- Acetylene Reduction assay

9.5 Seed ball use

Response variables (Y)	Explanatory Variables (Qualitative)	Explanatory Variables (Quantitative)	Inertia			Results of the permutation test		
NSB, EC, pH, OC, AN, P	CON<br>SB<br>VCPM	NSD, NST, SEm, SEt, CB, RET, Ht, NL, DLSS, IPS/EPS		Value	%	Permutations	Pseudo F	*p*-value
			Total	6.000	100.000	500	0.369	**< 0.0001**
			Constrained	1.618	26.963			
			Unconstrained	4.382	73.037			

NSD- Number of seed ball dispersed, NST- Number of seedlings transplanted, SEm- Number of seedlings emerged from seed balls, SEt- Number of seedlings established from transplanted seedlings, CB- Cost benefit, RET- Reduction in establishment time, Ht- Height of seedlings, NL- Number of leaves, DLSS- Decrease in use of less saline soil, IPS/EPS- Emergence % of seedlings from seed balls/ establishment % of transplanted seedlings, SB- seed ball technology, Con- Conventional technology, VCPM- Very closely planted mangrove seedlings (≤ 5cm.), NSB- Number of seeds in a ball, EC- Electrical conductivity, OC-Organic Carbon %, AN- Ammonia nitrogen, P- Phosphorus

Supplementary Table 10:

Table 10.1: Results of Statistical Hypothesis Testing of null hypotheses of the equality of average known variable values between various pairs of groups formed under Grass assisted stabilization

Group 1	Group 2	*t*-statistic value	*p*-value
**Variable -SDI**			
MHGC	MMGC	5.855	4.461e-06(**)
MHGC	MLGC	8.8285	5.283e-09(**)
MMGC	MLGC	-4.7835	7.201e-05(**)
**Variable -GCP**			
MHGC	MMGC	58.584	< 2.2e-16(**)
MHGC	MLGC	176.17	< 2.2e-16(**)
MMGC	MLGC	30.266	< 2.2e-16(**)
**Variable -Sa**			
MHGC	MMGC	5.4269	1.127e-05(**)
MHGC	MLGC	4.949	3.917e-05(**)
MHGC	MNGI	-5.5991	4.097e-06(**)
MMGC	MLGC	-1.7323	0.08964(#)
MMGC	MNGI	-27.485	< 2.2e-16(**)
MLGC	MNGI	-26.273	< 2.2e-16(**)
**Variable -S**			
MHGC	MMGC	4.0726	0.0002939(**)
MHGC	MLGC	-4.5593	7.142e-05(**)
MHGC	MNGI	1.466	0.1508(#)
MMGC	MLGC	-16.296	< 2.2e-16(**)
MMGC	MNGI	-3.8813	0.0003547(**)
MLGC	MNGI	9.3798	4.923e-12(**)
**Variable -C**			
MHGC	MMGC	-5.9667	2.603e-06(**)
MHGC	MLGC	-1.2252	0.2314(#)
MHGC	MNGI	3.1415	0.003849(*)
MMGC	MLGC	15.794	< 2.2e-16(**)
MMGC	MNGI	23.796	< 2.2e-16(**)
MLGC	MNGI	11.585	1.481e-14(**)

(*) and (**) refers to the relevant null hypothesis that is not valid at 5% and 0.1% levels of significance, respectively. (#) refers to the relevant null hypothesis being valid at 5% level of significance

MNGI- Monospecies with no grass at initial stage, MLGC- Multispecies with low grass coverage, MHGC- Multispecies with high grass coverage, MMGC- Multispecies with medium grass coverage, GCP- Grass cover %, SDI- Shannon diversity index, Sa- Sand %, S- Silt %, C- Clay %

Table 10.2: Results of Statistical Hypothesis Testing of null hypotheses of the equality of average unknown variable values between various pairs of groups formed under Grass assisted stabilization

Group 1	Group 2	*t*-statistic value	*p*-value
**Variable -TPD**			
MHGC	MMGC	-0.087094	0.931(#)
MHGC	MLGC	8.3079	1.716e-09(**)
MHGC	MNGI	11.757	9.085e-12(**)
MMGC	MLGC	9.219	9.554e-11(**)
MMGC	MNGI	13.166	6.217e-13(**)
MLGC	MNGI	6.4176	3.313e-07(**)
**Variable -SDDTP**			
MHGC	MMGC	2.9449	0.005484(*)
MHGC	MLGC	9.6012	4.055e-10(**)
MHGC	MNGI	11.672	1.421e-11(**)
MMGC	MLGC	10.432	9.613e-12(**)
MMGC	MNGI	14.289	5.643e-14(**)
MLGC	MNGI	7.172	1.425e-08(**)
**Variable -SPTP**			
MHGC	MMGC	3.1727	0.002734(*)
MHGC	MLGC	5.739	6.388e-07(**)
MHGC	MNGI	6.5982	4.742e-08(**)
MMGC	MLGC	1.5625	0.1255(#)
MMGC	MNGI	3.1565	0.002762(*)
MLGC	MNGI	2.1335	0.03885(*)
**Variable -SSR**			
MHGC	MMGC	3.546	0.0008861(**)
MHGC	MLGC	14.597	< 2.2e-16(**)
MHGC	MNGI	20.6	< 2.2e-16(**)
MMGC	MLGC	11.081	1.345e-14(**)
MMGC	MNGI	17.093	< 2.2e-16(**)
MLGC	MNGI	6.078	2.037e-07(**)
**Variable -EDen**			
MHGC	MMGC	25.838	< 2.2e-16(**)
MHGC	MLGC	22.864	< 2.2e-16(**)
MHGC	MNGI	25.163	< 2.2e-16(**)
MMGC	MLGC	-1.4216	0.1643(#)
MMGC	MNGI	1.3811	0.1753(#)
MLGC	MNGI	2.22	0.03139(*)
**Variable -ESR**			
MHGC	MMGC	18.887	< 2.2e-16(**)
MHGC	MLGC	31.602	< 2.2e-16(**)
MHGC	MNGI	35.489	< 2.2e-16(**)
MMGC	MLGC	4.0401	0.0002602(**)
MMGC	MNGI	3.8693	0.0005509(**)
MLGC	MNGI	-0.75972	0.4517(#)
**Variable -OC**			
MHGC	MMGC	1.6292	0.1113(#)
MHGC	MLGC	7.8063	6.228e-09(**)
MHGC	MNGI	-0.22741	0.8212(#)
MMGC	MLGC	3.0273	0.005371(*)
MMGC	MNGI	-1.9002	0.06628(#)

MLGC	MNGI	-10.394	9.529e-13(**)
**Variable -P**			
MHGC	MMGC	3.4401	0.001268(*)
MHGC	MLGC	7.8392	1.498e-08(**)
MHGC	MNGI	10.468	4.323e-11(**)
MMGC	MLGC	1.8533	0.07505(#)
MMGC	MNGI	3.8327	0.0007215(**)
MLGC	MNGI	6.6397	2.703e-08(**)
**Variable -AN**			
MHGC	MMGC	1.429	0.1599(#)
MHGC	MLGC	0.86269	0.394(#)
MHGC	MNGI	1.0966	0.2814(#)
MMGC	MLGC	-0.90932	0.3683(#)
MMGC	MNGI	-0.74567	0.4608(#)
MLGC	MNGI	0.29039	0.7729(#)

(*) and (**) refers to the relevant null hypothesis that is not valid at 5% and 0.1% levels of significance, respectively. (#) refers to the relevant null hypothesis being valid at 5% level of significance

MNGI- Monospecies with no grass at initial stage, MLGC- Multispecies with low grass coverage, MHGC- Multispecies with high grass coverage, MMGC- Multispecies with medium grass coverage, OC-Organic Carbon %, AN- Ammonia nitrogen, EDen- Epifaunal Density per unit area, ESR- Epifaunal species richness, P- Phosphorus, TPD- Trapped propagules density, SDDTP- Seedling density developed from trapped propagule, SPTP- Survival % of trapped propagules, SSR- Seedlings species richness

Table 10.3: Results of Statistical Hypothesis Testing of null hypotheses of the equality of average known variable values between various pairs of groups formed under Multispecies assemblage

Group 1	Group 2	*t*-statistic value	*p*-value
**Variable -SR**			
PMM	MSM	147.87	< 2.2e-16(**)
PMM	SRMM	10.198	1.464e-12(**)
**Variable -SDI**			
PMM	MSM	92.637	< 2.2e-16(**)
PMM	SRMM	9.1893	2.652e-11(**)
**Variable -EC**			
PMM	MSM	8.1701	2.675e-11(**)
PMM	SRMM	-8.8336	2.969e-13(**)
SRMM	MSM	17.587	< 2.2e-16(**)
**Variable -Sa**			
PMM	MSM	-13.673	< 2.2e-16(**)
PMM	SRMM	-17.337	< 2.2e-16(**)
SRMM	MSM	0.8132	0.4195(#)
**Variable -S**			
PMM	MSM	-1.0058	0.3204(#)
PMM	SRMM	-2.0639	0.04543(*)
SRMM	MSM	4.8342	6.608e-06(**)
**Variable -C**			
PMM	MSM	5.0824	8.625e-06(**)
PMM	SRMM	6.1067	3.327e-07(**)

SRMM	MSM	-5.0083	3.811e-06(**)

(*) and (**) refers to the relevant null hypothesis that is not valid at 5% and 0.1% levels of significance, respectively. (#) refers to the relevant null hypothesis being valid at 5% level of significance

MSM- Monospecies mangroves, SRMM- Semi-restored multispecies mangroves, PMM- Pristine multispecies mangroves, EC- Electrical conductivity, SDI- Shannon diversity index, SR- Species richness, Sa- Sand %, S- Silt %, C- Clay %

Table 10.4: Results of Statistical Hypothesis Testing of null hypotheses of the equality of average variable values between various pairs of groups formed under Multispecies assemblage

Group 1	Group 2	*t*-statistic value	*p*-value
**Variable -SPS**			
PMM	MSM	6.3617	1.63e-07(**)
PMM	SRMM	0.51792	0.606(#)
SRMM	MSM	5.5222	2.385e-06(**)
**Variable -CPS**			
PMM	MSM	26.685	< 2.2e-16(**)
PMM	SRMM	6.4642	2.059e-08(**)
SRMM	MSM	8.7835	2.158e-11(**)
**Variable -PL**			
PMM	MSM	6.5266	6.175e-09(**)
PMM	SRMM	-0.62773	0.532(#)
SRMM	MSM	6.719	2.842e-09(**)
**Variable -PF**			
PMM	MSM	28.697	< 2.2e-16(**)
PMM	SRMM	15.202	< 2.2e-16(**)
SRMM	MSM	9.7579	2.879e-14(**)
**Variable -PSR**			
PMM	MSM	63.278	< 2.2e-16(**)
PMM	SRMM	5.3489	1.815e-06(**)
SRMM	MSM	24.759	< 2.2e-16(**)
**Variable -EDen**			
PMM	MSM	19.37	< 2.2e-16(**)
PMM	SRMM	0.56711	0.5723(#)
SRMM	MSM	19.808	< 2.2e-16(**)
**Variable -ESR**			
PMM	MSM	27.764	< 2.2e-16(**)
PMM	SRMM	3.6809	0.0004766(**)
SRMM	MSM	35.519	< 2.2e-16(**)
**Variable -OC**			
PMM	MSM	7.2359	2.947e-10(**)
PMM	SRMM	5.9376	7.587e-08(**)
SRMM	MSM	1.0035	0.3188(#)
**Variable -P**			
PMM	MSM	1.6042	0.1167(#)
PMM	SRMM	1.2605	0.215(#)
SRMM	MSM	11.523	< 2.2e-16(**)
**Variable -AN**			
PMM	MSM	1.9182	0.06088(#)

PMM	SRMM	9.7532	3.792e-15(**)
SRMM	MSM	-2.809	0.007128(*)
**Variable -NRSD**			
PMM	MSM	22.687	< 2.2e-16(**)
PMM	SRMM	17.505	< 2.2e-16(**)
SRMM	MSM	65.012	< 2.2e-16(**)
**Variable -SRSSR**			
PMM	MSM	70.805	< 2.2e-16(**)
PMM	SRMM	30.721	< 2.2e-16(**)
SRMM	MSM	107.08	< 2.2e-16(**)

(*) and (**) refers to the relevant null hypothesis that is not valid at 5% and 0.1% levels of significance, respectively. (#) refers to the relevant null hypothesis being valid at 5% level of significance

MSM- Monospecies mangroves, SRMM- Semi-restored multispecies mangroves, PMM- Pristine multispecies mangroves, OC- Organic Carbon %, AN- Ammonia nitrogen, EDen- Epifaunal Density, ESR- Epifaunal species richness, P- Phosphorus, CPS- Cross-pollination success, SPS- Self-pollination success, PF- Pollinator frequency, PL- Pollen load, NRSD- Naturally regenerated seedling density, SRSSR- Naturally regenerated seedling species richness, PSR- Pollinator's species richness

Table 10.5: Results of Statistical Hypothesis Testing of null hypotheses of the equality of average known variable values between various pairs of groups formed under Facilitative Interaction

Group 1	Group 2	*t*-statistic value	*p*-value
**Variable -NC**			
VCPM	DPM	4.3426	0.0001667(**)
VCPM	CPM	4.3426	0.0001667(**)
**Variable -NSAC**			
VCPM	DPM	7.5422	3.245e-08(**)
VCPM	CPM	7.5422	3.245e-08(**)
**Variable -EC**			
VCPM	DPM	1.192	0.2383(#)
VCPM	CPM	1.0386	0.3035(#)
CPM	DPM	0.05422	0.957(#)
**Variable -pH**			
VCPM	DPM	-0.23415	0.8157(#)
VCPM	CPM	-0.54896	0.5852(#)
CPM	DPM	0.30981	0.7579(#)
**Variable -GB**			
VCPM	DPM	-6.9305	1.259e-07(**)
VCPM	CPM	-5.3879	8.089e-06(**)
CPM	DPM	-1.8943	0.06353(#)
**Variable -SSR**			
VCPM	DPM	-3.3247	0.001581(*)
VCPM	CPM	-2.093	0.04109(*)
CPM	DPM	-1.0042	0.3196(#)
**Variable - OC**			
VCPM	DPM	-0.68396	0.4969(#)
VCPM	CPM	-0.60182	0.5498(#)
CPM	DPM	-0.095014	0.9246(#)
**Variable -P**			
VCPM	DPM	-0.65589	0.5147(#)

VCPM	CPM	-0.50928	0.6127(#)
CPM	DPM	-0.088297	0.93(#)
**Variable -AN**			
VCPM	DPM	-0.50766	0.6137(#)
VCPM	CPM	-0.42046	0.6758(#)
CPM	DPM	-0.10414	0.9174(#)

(*) and (**) refers to the relevant null hypothesis that is not valid at 5% and 0.1% levels of significance, respectively. (#) refers to the relevant null hypothesis being valid at 5% level of significance

VCPM- Very closely planted mangroves (≤5cm.), CPM- Closely planted mangroves (≤ 40cm.), DPM- Distantly planted mangroves (≥100cm.), OC-Organic Carbon %, AN- Ammonia nitrogen, P- Phosphorus, EC- Electrical conductivity, GB- Glycine betaine, SSR- Soluble sugar-starch ratio, NSAC- Number of seeds/seedlings aggregated in a clump, NC- Number of clumps

Table 10.6: Results of Statistical Hypothesis Testing of null hypotheses of the equality of average unknown variable values between various pairs of groups formed under Facilitative Interaction

Group 1	Group 2	*t*-statistic value	*p*-value
**Variable -NST**			
VCPM	DPM	2.2002	0.03549(*)
VCPM	CPM	1.9985	0.05438(#)
CPM	DPM	0.63947	0.5252(#)
**Variable -NSG**			
VCPM	DPM	2.5285	0.01601(*)
VCPM	CPM	2.0584	0.046(*)
CPM	DPM	0.64434	0.5221(#)
**Variable -NSE**			
VCPM	DPM	2.745	0.009648(*)
VCPM	CPM	2.1617	0.03678(*)
CPM	DPM	0.87979	0.3832(#)
**Variable -SGP**			
VCPM	DPM	1.6152	0.1119(#)
VCPM	CPM	0.82873	0.4108(#)
CPM	DPM	0.68977	0.4932(#)
**Variable -EP**			
VCPM	DPM	4.0835	0.0001462(**)
VCPM	CPM	2.4636	0.01701(*)
CPM	DPM	2.2042	0.0322(*)
**Variable -PHt**			
VCPM	DPM	0.99212	0.3254(#)
VCPM	CPM	0.4711	0.6394(#)
CPM	DPM	0.54914	0.5851(#)
**Variable -NL**			
VCPM	DPM	2.1624	0.0349(*)
VCPM	CPM	1.5759	0.1207(#)
CPM	DPM	0.64988	0.5185(#)

(*) and (**) refers to the relevant null hypothesis that is not valid at 5% and 0.1% levels of significance, respectively. (#) refers to the relevant null hypothesis being valid at 5% level of significance

VCPM- Very closely planted mangroves (≤5cm.), CPM- Closely planted mangroves (≤ 40cm.), DPM- Distantly planted mangroves (≥100cm.), NSG- Number of seeds germinated, NST- Number of seeds/seedlings transplanted, NSE- Number of seeds/seedlings established, NL- Number of leaves, EP- Establishment percentage, PHt- Present height, SGP- Seed germination %

Table 10.7: Results of Statistical Hypothesis Testing of null hypotheses of the equality of average known variable values between various pairs of groups formed under Growth promotion by onsite PGPR consortia addition

Group 1	Group 2	*t*-statistic value	*p*-value
**Variable -IAA**			
BC1	BC2	-1.2467	0.2185(#)
BC1	BC3	0.6975	0.4888(#)
BC2	BC3	2.0969	0.0413(*)
**Variable -PSB**			
BC1	BC2	-2.1274	0.03856(*)
BC1	BC3	0.25999	0.796(#)
BC2	BC3	4.6065	3.035e-05(**)
**Variable -ACCD**			
BC1	BC2	-1.5778	0.1212(#)
BC1	BC3	-1.5717	0.1226(#)
BC2	BC3	0.9019	0.3716(#)
**Variable -SID**			
BC1	BC2	-3.0911	0.003316(*)
BC1	BC3	0.90093	0.3721(#)
BC2	BC3	5.1025	5.681e-06(**)
**Variable -ARA**			
BC1	BC2	0.83409	0.4084(#)
BC1	BC3	1.6957	0.09641(#)
BC2	BC3	4.5676	3.455e-05(**)

(*) and (**) refers to the relevant null hypothesis that is not valid at 5% and 0.1% levels of significance, respectively. (#) refers to the relevant null hypothesis being valid at 5% level of significance

WC-Without consortium, BC1- BC1 consortium, BC2- BC2 consortium, BC3- BC3 consortium, SID- Siderophore %, IAA- Indole Acetic Acid Produced, PSB- P-solubilization, ACCD- ACC deaminase units, ARA- Acetylene Reduction assay

Table 10.8: Results of Statistical Hypothesis Testing of null hypotheses of the equality of average unknown variable values between various pairs of groups formed under Growth promotion by onsite PGPR consortia addition

Group 1	Group 2	*t*-statistic value	*p*-value
**Variable - SH**			
BC1	BC2	-2.6212	0.01171(*)
BC1	BC3	-2.3803	0.02175(*)
BC1	WC	1.9291	0.05987(#)
BC2	BC3	0.61558	0.5413(#)
BC2	WC	4.3239	8.352e-05(**)
BC3	WC	4.2105	0.0001466(**)
**Variable - LW**			
BC1	BC2	-3.6898	0.0005739(**)

BC1	BC3	0.067778	0.9463(#)
BC1	WC	1.416	0.1633(#)
BC2	BC3	4.7575	2.685e-05(**)
BC2	WC	5.3237	2.659e-06(**)
BC3	WC	1.6884	0.09922(#)
**Variable - OC**			
BC1	BC2	-14.351	2.741e-15(**)
BC1	BC3	-30.385	< 2.2e-16(**)
BC1	WC	21.451	< 2.2e-16(**)
BC2	BC3	-31.506	< 2.2e-16(**)
BC2	WC	52.725	< 2.2e-16(**)
BC3	WC	76.069	< 2.2e-16(**)
**Variable - P**			
BC1	BC2	-15.988	< 2.2e-16(**)
BC1	BC3	-40.271	< 2.2e-16(**)
BC1	WC	91.357	< 2.2e-16(**)
BC2	BC3	-14.702	< 2.2e-16(**)
BC2	WC	91.882	< 2.2e-16(**)
BC3	WC	151.19	< 2.2e-16(**)
**Variable - AN**			
BC1	BC2	9.3769	2.664e-12(**)
BC1	BC3	-22.043	2.664e-12(**)
BC1	WC	84.202	< 2.2e-16(**)
BC2	BC3	-34.892	< 2.2e-16(**)
BC2	WC	83.165	< 2.2e-16(**)
BC3	WC	125.82	< 2.2e-16(**)
**Variable - NN**			
BC1	BC2	2.5107	0.0158(*)
BC1	BC3	-2.9949	0.004441(*)
BC1	WC	21.827	< 2.2e-16(**)
BC2	BC3	-5.394	3.876e-06(**)
BC2	WC	23.49	< 2.2e-16(**)
BC3	WC	21.42	< 2.2e-16(**)

(*) and (**) refers to the relevant null hypothesis that is not valid at 5% and 0.1% levels of significance, respectively. (#) refers to the relevant null hypothesis being valid at 5% level of significance

WC-Without consortium, BC1- BC1 consortium, BC2- BC2 consortium, BC3- BC3 consortium, LW-Leaf width, SH-Shoot height, AN-Ammonia nitrogen, NN-Nitrate nitrogen, P-Phosphorus, OC-Organic carbon %

Table 10.9: Results of Statistical Hypothesis Testing of null hypotheses of the equality of average known variable values between various pairs of groups formed under Seed ball use

Group 1	Group 2	*t*-statistic value	*p*-value
**Variable -NSB/AS**			
SB	Con	6.9955	1.083e-07(**)
SB	VCPM	-3.1885	0.002696(*)
VCPM	Con	7.1277	7.635e-08(**)
**Variable -EC**			
SB	Con	-1.6799	0.09837(#)
SB	VCPM	-1.3389	0.1862(#)

VCPM	Con	-0.14355	0.8864(#)
**Variable -pH**			
SB	Con	1.3445	0.1842(#)
SB	VCPM	2.2695	0.02889(*)
VCPM	Con	-1.3818	0.1741(#)
**Variable -OC**			
SB	Con	0.76717	0.4465(#)
SB	VCPM	4.7486	1.386e-05(**)
VCPM	Con	-4.7689	1.571e-05(**)
**Variable -P**			
SB	Con	1.6196	0.1112(#)
SB	VCPM	2.1578	0.03546(*)
VCPM	Con	-1.0363	0.3055(#)
**Variable -AN**			
SB	Con	0.027445	0.9782(#)
SB	VCPM	0.11528	0.9087(#)
VCPM	Con	-0.11738	0.907(#)

(*) and (**) refers to the relevant null hypothesis that is not valid at 5% and 0.1% levels of significance, respectively. (#) refers to the relevant null hypothesis being valid at 5% level of significance

SB- seed ball technology, Con- Conventional technology, VCPM- Very closely planted mangroves (≤5cm.), OC-Organic Carbon %, AN- Ammonia nitrogen, P- Phosphorus, EC- Electrical conductivity, NSB/AS- Number of seeds in a ball/Aggregated seedlings

Table 10.10: Results of Statistical Hypothesis Testing of null hypotheses of the equality of average unknown values between various pairs of groups formed under Seed ball use

Group 1	Group 2	*t*-statistic value	*p*-value
**Variable -NSD**			
SB	Con	4.4184	0.0001357(**)
SB	VCPM	3.896	0.0004613(**)
VCPM	Con	1	0.3253(#)
**Variable -NST**			
SB	Con	-5.0392	2.275e-05(**)
SB	VCPM	-2.9196	0.006591(*)
VCPM	Con	2.0171	0.05225(#)
**Variable -SEm**			
SB	Con	3.5205	0.001495(*)
SB	VCPM	3.1407	0.003698(*)
VCPM	Con	1	0.3253(#)
**Variable -SEt**			
SB	Con	-4.6421	6.853e-05(**)
SB	VCPM	-3.5433	0.001316(*)
VCPM	Con	1.5176	0.1371(#)
**Variable -IPS/EPS**			
SB	Con	-11.204	1.232e-15(**)
SB	VCPM	-7.369	2.259e-09(**)
VCPM	Con	-1.6542	0.1038(#)
**Variable -RET**			
SB	Con	73.11	< 2.2e-16(**)
SB	VCPM	27.899	< 2.2e-16(**)
VCPM	Con	1	0.3253(#)
**Variable -CB**			

SB	Con	32.996	< 2.2e-16(**)
SB	VCPM	23.02	< 2.2e-16(**)
VCPM	Con	1	0.3253(#)
**Variable -HT**			
SB	Con	-2.3458	0.02404(*)
SB	VCPM	-3.2288	0.002504(*)
VCPM	Con	0.81409	0.4189(#)
**Variable -NL**			
SB	Con	-5.9382	1.91e-07(**)
SB	VCPM	-5.1248	3.586e-06(**)
VCPM	Con	-0.59815	0.552(#)
**Variable -DLSS**			
SB	Con	28.833	< 2.2e-16(**)
SB	VCPM	20.245	< 2.2e-16(**)
VCPM	Con	1	0.3253(#)

(*) and (**) refers to the relevant null hypothesis that is not valid at 5% and 0.1% levels of significance, respectively. (#) refers to the relevant null hypothesis being valid at 5% level of significance

SB- seed ball technology, Con- Conventional technology, VCPM- Very closely planted mangroves (≤5cm.), NL- No. of leaves, NSD- No. of seed balls dispersed, NST- No. of seedlings transplanted, SEm- No. of seedlings emerged from seed balls, SEt- No. of seedlings established, CB- Cost benefit of transplantation, RET- Reduction in establishment time, DLSS- Decrease in use of less-saline soil, IPS/EPS- Emergence % of seedlings from seed balls/ establishment % of transplanted seedlings, Ht- Height

Supplementary Table 11: ANOVA table for all graphical representations

No. of epifaunal species (n=10)

Forest type		*p* value
Degraded	Ramganga 2014	.000
	Ramganga 2016	.787
	Ramganga 2021	.000
	Ramganga 2022	.000
	Pristine	.000
	Monospecies	1.000
Ramganga 2014	Degraded	.000
	Ramganga 2016	.000
	Ramganga 2021	.000
	Ramganga 2022	.000
	Pristine	.000
	Monospecies	.000
Ramganga 2016	Degraded	.787
	Ramganga 2014	.000
	Ramganga 2021	.000
	Ramganga 2022	.000
	Pristine	.000
	Monospecies	.897
Ramganga 2021	Degraded	.000
	Ramganga 2014	.000
	Ramganga 2016	.000
	Ramganga 2022	.643
	Pristine	.042
	Monospecies	.000
Ramganga 2022	Degraded	.000
	Ramganga 2014	.000
	Ramganga 2016	.000
	Ramganga 2021	.643
	Pristine	.001
	Monospecies	.000
Pristine	Degraded	.000
	Ramganga 2014	.000
	Ramganga 2016	.000
	Ramganga 2021	.042
	Ramganga 2022	.001
	Monospecies	.000
Monospecies	Degraded	1.000

	Ramganga 2014	.000
	Ramganga 2016	.897
	Ramganga 2021	.000
	Ramganga 2022	.000
	Pristine	.000

No. of epifaunal individuals*10^5 ha^{-1} (n=10)

Forest type		*p* value
Degraded	Ramganga 2014	.426
	Ramganga 2016	.212
	Ramganga 2021	.000
	Ramganga 2022	.000
	Pristine	.000
	Monospecies	.000
Ramganga 2014	Degraded	.426
	Ramganga 2016	1.000
	Ramganga 2021	.000
	Ramganga 2022	.000
	Pristine	.000
	Monospecies	.000
Ramganga 2016	Degraded	.212
	Ramganga 2014	1.000
	Ramganga 2021	.000
	Ramganga 2022	.000
	Pristine	.000
	Monospecies	.000
Ramganga 2021	Degraded	.000
	Ramganga 2014	.000
	Ramganga 2016	.000
	Ramganga 2022	.418
	Pristine	.043
	Monospecies	.000
Ramganga 2022	Degraded	.000
	Ramganga 2014	.000
	Ramganga 2016	.000
	Ramganga 2021	.418
	Pristine	.927
	Monospecies	.000
Pristine	Degraded	.000
	Ramganga 2014	.000

	Ramganga 2016	.000
	Ramganga 2021	.043
	Ramganga 2022	.927
	Monospecies	.000
Monospecies	Degraded	.000
	Ramganga 2014	.000
	Ramganga 2016	.000
	Ramganga 2021	.000
	Ramganga 2022	.000
	Pristine	.000

No. of species of regenerated seedlings (n=10)

Forest type		*p* value
Degraded	Ramganga 2014	1.000
	Ramganga 2016	.005
	Ramganga 2021	.000
	Ramganga 2022	.000
	Pristine	.000
	Monospecies	.000
Ramganga 2014	Degraded	1.000
	Ramganga 2016	.002
	Ramganga 2021	.000
	Ramganga 2022	.000
	Pristine	.000
	Monospecies	.000
Ramganga 2016	Degraded	.005
	Ramganga 2014	.002
	Ramganga 2021	.000
	Ramganga 2022	.000
	Pristine	.000
	Monospecies	.000
Ramganga 2021	Degraded	.000
	Ramganga 2014	.000
	Ramganga 2016	.000
	Ramganga 2022	.000
	Pristine	.000
	Monospecies	.000
Ramganga 2022	Degraded	.000
	Ramganga 2014	.000
	Ramganga 2016	.000

	Ramganga 2021	.000
	Pristine	.000
	Monospecies	.000
Pristine	Degraded	.000
	Ramganga 2014	.000
	Ramganga 2016	.000
	Ramganga 2021	.000
	Ramganga 2022	.000
	Monospecies	.000
Monospecies	Degraded	.000
	Ramganga 2014	.000
	Ramganga 2016	.000
	Ramganga 2021	.000
	Ramganga 2022	.000
	Pristine	.000

No. of regenerated seedlings*10^4 ha^{-1} (n=10)

**Forest type**		***p* value**
Degraded	Ramganga 2014	1.000
	Ramganga 2016	1.000
	Ramganga 2021	.063
	Ramganga 2022	.000
	Pristine	.000
	Monospecies	1.000
Ramganga 2014	Degraded	1.000
	Ramganga 2016	1.000
	Ramganga 2021	.022
	Ramganga 2022	.000
	Pristine	.000
	Monospecies	1.000
Ramganga 2016	Degraded	1.000
	Ramganga 2014	1.000
	Ramganga 2021	.041
	Ramganga 2022	.000
	Pristine	.000
	Monospecies	1.000
Ramganga 2021	Degraded	.063
	Ramganga 2014	.022
	Ramganga 2016	.041
	Ramganga 2022	.153

	Pristine	.000
	Monospecies	.028
Ramganga 2022	Degraded	.000
	Ramganga 2014	.000
	Ramganga 2016	.000
	Ramganga 2021	.153
	Pristine	.000
	Monospecies	.000
Pristine	Degraded	.000
	Ramganga 2014	.000
	Ramganga 2016	.000
	Ramganga 2021	.000
	Ramganga 2022	.000
	Monospecies	.000
Monospecies	Degraded	1.000
	Ramganga 2014	1.000
	Ramganga 2016	1.000
	Ramganga 2021	.028
	Ramganga 2022	.000
	Pristine	.000

Mangrove vegetation covered (m^2 ha^{-1}) (n=25)

**Forest type**		***p* value**
Degraded	Ramganga 2014	1.000
	Ramganga 2016	1.000
	Ramganga 2021	.430
	Ramganga 2022	.055
	Pristine	.000
Ramganga 2014	Degraded	1.000
	Ramganga 2016	.999
	Ramganga 2021	.379
	Ramganga 2022	.053
	Pristine	.000
Ramganga 2016	Degraded	1.000
	Ramganga 2014	.999
	Ramganga 2021	.589
	Ramganga 2022	.060
	Pristine	.000
Ramganga 2021	Degraded	.430
	Ramganga 2014	.379

	Ramganga 2016	.589
	Ramganga 2022	.467
	Pristine	.000
Ramganga 2022	Degraded	.065
	Ramganga 2014	.053
	Ramganga 2016	.060
	Ramganga 2021	.077
	Pristine	.379
Pristine	Degraded	.000
	Ramganga 2014	.000
	Ramganga 2016	.000
	Ramganga 2021	.000
	Ramganga 2022	.055

Mangrove cover (%) (n=25)

**Forest type**		***p* value**
Degraded	Ramganga 2014	1.000
	Ramganga 2016	1.000
	Ramganga 2021	.430
	Ramganga 2022	.055
	Pristine	.000
Ramganga 2014	Degraded	1.000
	Ramganga 2016	.999
	Ramganga 2021	.379
	Ramganga 2022	.053
	Pristine	.000
Ramganga 2016	Degraded	1.000
	Ramganga 2014	.999
	Ramganga 2021	.589
	Ramganga 2022	.060
	Pristine	.000
Ramganga 2021	Degraded	.430
	Ramganga 2014	.379
	Ramganga 2016	.589
	Ramganga 2022	.467
	Pristine	.000
Ramganga 2022	Degraded	.065
	Ramganga 2014	.053
	Ramganga 2016	.060
	Ramganga 2021	.077

	Pristine	.379
Pristine	Degraded	.000
	Ramganga 2014	.000
	Ramganga 2016	.000
	Ramganga 2021	.000
	Ramganga 2022	.055

No. of mangrove species (n=25)

Forest type		*p* value
Degraded	Ramganga 2014	.279
	Ramganga 2016	.000
	Ramganga 2021	.000
	Ramganga 2022	.000
	Pristine	.000
Ramganga 2014	Degraded	.279
	Ramganga 2016	.000
	Ramganga 2021	.000
	Ramganga 2022	.000
	Pristine	.000
Ramganga 2016	Degraded	.000
	Ramganga 2014	.000
	Ramganga 2021	.000
	Ramganga 2022	.000
	Pristine	.000
Ramganga 2021	Degraded	.000
	Ramganga 2014	.000
	Ramganga 2016	.000
	Ramganga 2022	.279
	Pristine	.000
Ramganga 2022	Degraded	.000
	Ramganga 2014	.000
	Ramganga 2016	.000
	Ramganga 2021	.279
	Pristine	.000
Pristine	Degraded	.000
	Ramganga 2014	.000
	Ramganga 2016	.000
	Ramganga 2021	.000
	Ramganga 2022	.000

Total basal area (m^2 ha^{-1}) (n=25)

Forest type		*p* value
Degraded	Ramganga 2014	1.000
	Ramganga 2016	1.000
	Ramganga 2021	.430
	Ramganga 2022	.055
	Pristine	.000
Ramganga 2014	Degraded	1.000
	Ramganga 2016	.999
	Ramganga 2021	.379
	Ramganga 2022	.053
	Pristine	.000
Ramganga 2016	Degraded	1.000
	Ramganga 2014	.999
	Ramganga 2021	.589
	Ramganga 2022	.060
	Pristine	.000
Ramganga 2021	Degraded	.430
	Ramganga 2014	.379
	Ramganga 2016	.589
	Ramganga 2022	.467
	Pristine	.000
Ramganga 2022	Degraded	.065
	Ramganga 2014	.053
	Ramganga 2016	.060
	Ramganga 2021	.077
	Pristine	.379
Pristine	Degraded	.000
	Ramganga 2014	.000
	Ramganga 2016	.000
	Ramganga 2021	.000
	Ramganga 2022	.055

Frequency of *Apis dorsata* (no. of visits mt^{-2} hr^{-1}) (n=25)

Forest type		*p* value
Degraded	Ramganga 2014	.000
	Ramganga 2016	.001
	Ramganga 2021	.000
	Ramganga 2022	.000
	Pristine	.000
Ramganga 2014	Degraded	.000
	Ramganga 2016	.000
	Ramganga 2021	.000
	Ramganga 2022	.000
	Pristine	.000
Ramganga 2016	Degraded	.001
	Ramganga 2014	.000
	Ramganga 2021	.000
	Ramganga 2022	.000
	Pristine	.000
Ramganga 2021	Degraded	.000
	Ramganga 2014	.000
	Ramganga 2016	.000
	Ramganga 2022	.000
	Pristine	.000
Ramganga 2022	Degraded	.000
	Ramganga 2014	.000
	Ramganga 2016	.000
	Ramganga 2021	.000
	Pristine	.001
Pristine	Degraded	.000
	Ramganga 2014	.000
	Ramganga 2016	.000
	Ramganga 2021	.000
	Ramganga 2022	.001

Frequency of *Micrapsis* sp. (no. of visits mt^{-2} hr^{-1}) (n=25)

**Forest type**		***p* value**
Degraded	Ramganga 2014	.000
	Ramganga 2016	.000
	Ramganga 2021	.000
	Ramganga 2022	.000
	Pristine	.000
Ramganga 2014	Degraded	.000
	Ramganga 2016	.000
	Ramganga 2021	.000
	Ramganga 2022	.000
	Pristine	.000
Ramganga 2016	Degraded	.000
	Ramganga 2014	.000
	Ramganga 2021	.000
	Ramganga 2022	.000
	Pristine	.000
Ramganga 2021	Degraded	.000
	Ramganga 2014	.000
	Ramganga 2016	.000
	Ramganga 2022	.000
	Pristine	.000
Ramganga 2022	Degraded	.000
	Ramganga 2014	.000
	Ramganga 2016	.000
	Ramganga 2021	.000
	Pristine	.000
Pristine	Degraded	.000
	Ramganga 2014	.000
	Ramganga 2016	.000
	Ramganga 2021	.000
	Ramganga 2022	.000

Shoot height (cm) (n=10)

Dosage		*p* value
Control initial	Control after experiment	.000
	BC1 after consortia addition	.000
	BC2 after consortia addition	.000
	BC3 after consortia addition	.000
Control after experiment	Control initial	.000
	BC1 after consortia addition	.512
	BC2 after consortia addition	.000
	BC3 after consortia addition	.000
BC1 after consortia addition	Control initial	.000
	Control after experiment	.512
	BC2 after consortia addition	.011
	BC3 after consortia addition	.128
BC2 after consortia addition	Control initial	.000
	Control after experiment	.000
	BC1 after consortia addition	.011
	BC3 after consortia addition	.849
BC3 after consortia addition	Control initial	.000
	Control after experiment	.000
	BC1 after consortia addition	.128
	BC2 after consortia addition	.849

Leaf length (cm) (n=10)

Dosage		*p* value
Control initial	Control after experiment	.003
	BC1 after consortia addition	.000
	BC2 after consortia addition	.000
	BC3 after consortia addition	.000
Control after experiment	Control initial	.003
	BC1 after consortia addition	.009
	BC2 after consortia addition	.000
	BC3 after consortia addition	.001
BC1 after consortia addition	Control initial	.000
	Control after experiment	.009
	BC2 after consortia addition	.000
	BC3 after consortia addition	.960
BC2 after consortia addition	Control initial	.000
	Control after experiment	.000
	BC1 after consortia addition	.000
	BC3 after consortia addition	.000
BC3 after consortia addition	Control initial	.000
	Control after experiment	.001
	BC1 after consortia addition	.960
	BC2 after consortia addition	.000

Leaf width (cm) (n=10)

Dosage		*p* value
Control initial	Control after experiment	.005
	BC1 after consortia addition	.000
	BC2 after consortia addition	.000
	BC3 after consortia addition	.001
Control after experiment	Control initial	.005
	BC1 after consortia addition	.333
	BC2 after consortia addition	.137
	BC3 after consortia addition	.989
BC1 after consortia addition	Control initial	.000
	Control after experiment	.333
	BC2 after consortia addition	.991
	BC3 after consortia addition	.629
BC2 after consortia addition	Control initial	.000
	Control after experiment	.137
	BC1 after consortia addition	.991
	BC3 after consortia addition	.343
BC3 after consortia addition	Control initial	.001
	Control after experiment	.989
	BC1 after consortia addition	.629
	BC2 after consortia addition	.343

Organic carbon (%) (n=10)

Treatment	Dosage		*p* value
Control	Before consortia addition	After 70 days of consortia addition	.000
		After 122 days of consortia addition	.000
	After 70 days of consortia addition	Before consortia addition	.000
		After 122 days of consortia addition	.058
	After 122 days of consortia addition	Before consortia addition	.000
		After 70 days of consortia addition	.058
BC1	Before consortia addition	After 70 days of consortia addition	.000
		After 122 days of consortia addition	.000
	After 70 days of consortia addition	Before consortia addition	.000
		After 122 days of consortia addition	.125
	After 122 days of consortia addition	Before consortia addition	.000
		After 70 days of consortia addition	.125
BC2	Before consortia addition	After 70 days of consortia addition	.000
		After 122 days of consortia addition	.000
	After 70 days of consortia addition	Before consortia addition	.000
		After 122 days of consortia addition	.010
	After 122 days of consortia addition	Before consortia addition	.000
		After 70 days of consortia addition	.010
BC3	Before consortia addition	After 70 days of consortia addition	.000
		After 122 days of consortia addition	.000
	After 70 days of consortia addition	Before consortia addition	.000
		After 122 days of consortia addition	.000
	After 122 days of consortia addition	Before consortia addition	.000

		After 70 days of consortia addition	.000

Plant available phosphorus-P (mg kg^{-1}) (n=10)

Treatment	Dosage		*p* value
Control	Before consortia addition	After 70 days of consortia addition	.000
		After 122 days of consortia addition	.000
	After 70 days of consortia addition	Before consortia addition	.000
		After 122 days of consortia addition	.000
	After 122 days of consortia addition	Before consortia addition	.000
		After 70 days of consortia addition	.000
BC1	Before consortia addition	After 70 days of consortia addition	.000
		After 122 days of consortia addition	.000
	After 70 days of consortia addition	Before consortia addition	.000
		After 122 days of consortia addition	.000
	After 122 days of consortia addition	Before consortia addition	.000
		After 70 days of consortia addition	.000
BC2	Before consortia addition	After 70 days of consortia addition	.744
		After 122 days of consortia addition	.000
	After 70 days of consortia addition	Before consortia addition	.744
		After 122 days of consortia addition	.000
	After 122 days of consortia addition	Before consortia addition	.000
		After 70 days of consortia addition	.000
BC3	Before consortia addition	After 70 days of consortia addition	.000
		After 122 days of consortia addition	.000
	After 70 days of consortia addition	Before consortia addition	.000
		After 122 days of consortia addition	.001

	After 122 days of consortia addition	Before consortia addition	.000
		After 70 days of consortia addition	.001

Ammonia-N (mg kg^{-1}) (n=10)

Treatment	Dosage		*p* value
Control	Before consortia addition	After 70 days of consortia addition	.000
		After 122 days of consortia addition	.000
	After 70 days of consortia addition	Before consortia addition	.000
		After 122 days of consortia addition	.000
	After 122 days of consortia addition	Before consortia addition	.000
		After 70 days of consortia addition	.000
BC1	Before consortia addition	After 70 days of consortia addition	.000
		After 122 days of consortia addition	.000
	After 70 days of consortia addition	Before consortia addition	.000
		After 122 days of consortia addition	.000
	After 122 days of consortia addition	Before consortia addition	.000
		After 70 days of consortia addition	.000
BC2	Before consortia addition	After 70 days of consortia addition	.000
		After 122 days of consortia addition	.000
	After 70 days of consortia addition	Before consortia addition	.000
		After 122 days of consortia addition	.298
	After 122 days of consortia addition	Before consortia addition	.000
		After 70 days of consortia addition	.298
BC3	Before consortia addition	After 70 days of consortia addition	.000
		After 122 days of consortia addition	.000
	After 70 days of consortia addition	Before consortia addition	.000

		After 122 days of consortia addition	.000
	After 122 days of consortia addition	Before consortia addition	.000
		After 70 days of consortia addition	.000

Nitrate-N (mg kg^{-1}) (n=10)

Treatment	Dosage		*p* value
Control	Before consortia addition	After 70 days of consortia addition	.000
		After 122 days of consortia addition	.000
	After 70 days of consortia addition	Before consortia addition	.000
		After 122 days of consortia addition	.000
	After 122 days of consortia addition	Before consortia addition	.000
		After 70 days of consortia addition	.000
BC1	Before consortia addition	After 70 days of consortia addition	.000
		After 122 days of consortia addition	.000
	After 70 days of consortia addition	Before consortia addition	.000
		After 122 days of consortia addition	.003
	After 122 days of consortia addition	Before consortia addition	.000
		After 70 days of consortia addition	.003
BC2	Before consortia addition	After 70 days of consortia addition	.000
		After 122 days of consortia addition	.000
	After 70 days of consortia addition	Before consortia addition	.000
		After 122 days of consortia addition	.000
	After 122 days of consortia addition	Before consortia addition	.000
		After 70 days of consortia addition	.000
BC3	Before consortia addition	After 70 days of consortia addition	.000

		After 122 days of consortia addition	.000
	After 70 days of consortia addition	Before consortia addition	.000
		After 122 days of consortia addition	.058
	After 122 days of consortia addition	Before consortia addition	.000
		After 70 days of consortia addition	.058

IAA production (µg ml^{-1}) (n=12)

**Dosage**		***p* value**
BC1	BC2	.570
	BC3	.985
BC2	BC1	.570
	BC3	.470
BC3	BC1	.985
	BC2	.470

ACC deaminase (Units) (n=12)

**Dosage**		***p* value**
BC1	BC2	.480
	BC3	.717
BC2	BC1	.480
	BC3	.921
BC3	BC1	.717
	BC2	.921

Plant available phosphorus-P (µg ml^{-1}) (n=12)

**Dosage**		***p* value**
BC1	BC2	.251
	BC3	.987
BC2	BC1	.251
	BC3	.318
BC3	BC1	.987
	BC2	.318

Siderophore activity (%) (n=12)

Dosage		*p* value
BC1	BC2	.108
	BC3	.634
BC2	BC1	.108
	BC3	.014
BC3	BC1	.634
	BC2	.014

CFU count on FNF medium (n=12)

Dosage		*p* value
BC1	BC2	.275
	BC3	.006
BC2	BC1	.275
	BC3	.195
BC3	BC1	.006
	BC2	.195

Supplementary Table 12:

12.1: NGS metadata table of grass rhizosphere bacterial abundance

Sample	Status	Bioproject	Biosample	SRA
Ramganga 2014	Degraded Ramganga 2014	PRJNA836387	SAMN28159341	SRS12954358
Myriostachya wightiana	From established grass rhizosphere Ramganga 2021-22	PRJNA809777	SAMN26202177	SRS12101062
Paspalum vaginatum	From established grass rhizosphere Ramganga 2021-22	PRJNA809772	SAMN26202111	SRS12114902
Porteresia coarctata	From established grass rhizosphere Ramganga 2021-22	PRJNA809778	SAMN26202868	SRS12101103
Sporobolus virginicus	From established grass rhizosphere Ramganga 2021-22	PRJNA809773	SAMN26202168	SRS12091782
Ramganga 2016	Planted with grasses Ramganga 2016	PRJNA836387	SAMN30506754	SRS14840945

12.2: NGS metadata table of different Mangrove sediments

Sample	Status	Bioproject	Biosample	SRA
Ramganga 2014	Degraded	PRJNA836387	SAMN28159341	SRS12954358
Ramganga 2016	Degraded	PRJNA836387	SAMN30506754	SRS14840945
Ramganga 2021	Ramgana 2021-22 Semi-restored	PRJNA801402	SAMN25342833	SRS12077795
Ramganga 2022	Ramgana 2021-22 Semi-restored	PRJNA801402	SAMN26255136	SRS12140422
Bhagbatpur	Pristine	PRJNA809522	SAMN26183780	SRS12086291
Dashpur	Pristine	PRJNA809522	SAMN26183779	SRS12082328
Durbachoti	Degraded	PRJNA809569	SAMN26197017	SRS12087452
Gangapur	Degraded	PRJNA809569	SAMN26197018	SRS12091723
Harendranagar	Degraded	PRJNA809569	SAMN28159690	SRS12954429
Ramganga 2022_2	Ramgana 2021-22 Semi-restored	PRJNA801402	SAMN30504958	SRS14826460
Ramganga 2022_3	Ramgana 2021-22 Semi-restored	PRJNA801402	SAMN30504960	SRS14840947
Atharogazi mono-sp plantation	Monospecies Plantation	PRJNA809754	SAMN26200892	SRS12140406
Atharogazi mono-sp plantation _2	Monospecies Plantation	PRJNA809754	SAMN28159710	SRS12947643
Durbachoti mono-sp plantation	Monospecies Plantation	PRJNA809754	SAMN26200891	SRS12140377
Durbachoti mono-sp plantation_2	Monospecies Plantation	PRJNA809754	SAMN28159709	SRS12947644

References used:

1. Arifanti, V. B. *et al*. Challenges and Strategies for Sustainable Mangrove Management in Indonesia: A Review. Forests. 13, 695. https://doi.org/10.3390/f13050695 (2022).

2. Etigale, E. B., Ajayi, S., Udofia, S. I., Moses, M. U. Assessment of stand density and growth rate of three tree species in an arboretum within the University of Uyo, Nigeria. Journal of research in Forestry, Wildlife and Environment. 6(1). (2014).
